# Automation of systematic reviews of biomedical literature: a scoping review of studies indexed in PubMed

**DOI:** 10.1186/s13643-024-02592-3

**Published:** 2024-07-08

**Authors:** Barbara Tóth, László Berek, László Gulácsi, Márta Péntek, Zsombor Zrubka

**Affiliations:** 1 Doctoral School of Innovation Management, Óbuda University, Bécsi út 96/B, Budapest, 1034 Hungary; 2 Doctoral School for Safety and Security, Óbuda University, Bécsi út 96/B, Budapest, 1034 Hungary; 3 University Library, Óbuda University, Bécsi út 96/B, Budapest, 1034 Hungary; 4 HECON Health Economics Research Center, University Research, and Innovation Center, Óbuda University, Bécsi út 96/B, Budapest, 1034 Hungary

**Keywords:** Systematic literature review, Evidence synthesis, Automation, Artificial intelligence, Machine learning, Natural language processing, Text mining

## Abstract

**Background:**

The demand for high-quality systematic literature reviews (SRs) for evidence-based medical decision-making is growing. SRs are costly and require the scarce resource of highly skilled reviewers. Automation technology has been proposed to save workload and expedite the SR workflow. We aimed to provide a comprehensive overview of SR automation studies indexed in PubMed, focusing on the applicability of these technologies in real world practice.

**Methods:**

In November 2022, we extracted, combined, and ran an integrated PubMed search for SRs on SR automation. Full-text English peer-reviewed articles were included if they reported studies on SR automation methods (SSAM), or automated SRs (ASR). Bibliographic analyses and knowledge-discovery studies were excluded. Record screening was performed by single reviewers, and the selection of full text papers was performed in duplicate. We summarized the publication details, automated review stages, automation goals, applied tools, data sources, methods, results, and Google Scholar citations of SR automation studies.

**Results:**

From 5321 records screened by title and abstract, we included 123 full text articles, of which 108 were SSAM and 15 ASR. Automation was applied for search (19/123, 15.4%), record screening (89/123, 72.4%), full-text selection (6/123, 4.9%), data extraction (13/123, 10.6%), risk of bias assessment (9/123, 7.3%), evidence synthesis (2/123, 1.6%), assessment of evidence quality (2/123, 1.6%), and reporting (2/123, 1.6%). Multiple SR stages were automated by 11 (8.9%) studies. The performance of automated record screening varied largely across SR topics. In published ASR, we found examples of automated search, record screening, full-text selection, and data extraction. In some ASRs, automation fully complemented manual reviews to increase sensitivity rather than to save workload. Reporting of automation details was often incomplete in ASRs.

**Conclusions:**

Automation techniques are being developed for all SR stages, but with limited real-world adoption. Most SR automation tools target single SR stages, with modest time savings for the entire SR process and varying sensitivity and specificity across studies. Therefore, the real-world benefits of SR automation remain uncertain. Standardizing the terminology, reporting, and metrics of study reports could enhance the adoption of SR automation techniques in real-world practice.

**Supplementary Information:**

The online version contains supplementary material available at 10.1186/s13643-024-02592-3.

## Background

High-quality systematic literature reviews (SRs) and meta-analyses represent the highest level of evidence in evidence-based medicine, providing essential input to medical decision-making [1, 2]. While the number of published SRs in PubMed was 80 per day in 2019 [3], this number increased to 135 by 2021 [4]. The accelerated development of novel medical technologies such as software and digital devices [5, 6], virtual reality [7], and chatbots [8] will push further the demand for high-quality SRs [3, 9, 10]. Beyond medicine, systematic reviews are often performed in disciplines including engineering [11–13] or the social sciences [14, 15].

As the demand for SRs grows, keeping them up-to date is becoming increasingly challenging. The preparation of a SR is labor-intensive and time-consuming process requiring the scarce resources of highly skilled researchers. The typical lag for primary studies to be included in SRs is 2.5–6.5 years, delaying the translation of results to medical decision-making. Although the Cochrane Handbook recommends that SRs are updated biannually [16], 23% of SRs can become outdated within 2 years due to the omission of new evidence that could impact their conclusions [17].

SR automation using artificial intelligence (AI) and advanced computing technologies has the potential to speed up the review process, reduce the workload of researchers, prevent human errors, and facilitate reproducibility by diminishing the role of human judgement [18–20]. The feasibility of automation differs by stages of the SR workflow [21, 22], with search, record screening, full-text selection, data extraction, risk of bias assessment, evidence synthesis, and reporting being the most prominent examples [16, 21]. Automated assessment of evidence quality is also under investigation [23, 24]. Hence, recent SR methodological guidelines have addressed the use of automation tools. The Cochrane Handbook acknowledges the use of AI tools when updating SRs or using AI as a second reviewer alongside a human reviewer [16]. While the Handbook mentions active learning, it does not recommend its use on its own, and considers data extraction mainly as a manual process, despite citing some examples for automated data extraction. The latest PRISMA reporting standard also acknowledges the use of automation tools in record screening or priority ranking. It also sets out how to report the use of AI tools in the screening or risk of bias assessment stages of SR reports, including the training of the tool and the method used to measure its validity [25]. Automated risk of bias assessment is also a promising field for methodological innovation, but results are not yet convincing [3].

Despite some positive experiences, the uptake of SR automation tools is still limited [26, 27]. Trust in automated SRs is based on the availability of high-quality summary studies of their results. Accordingly, several authors have systematically reviewed automation technologies in various stages of the SR workflow. While aiming for a comprehensive summary, these studies differed in their focus, search strategies, and number of included reports. The topics covered text mining for screening [22], data extraction [28], any automated SR stage [29], or identifying high-quality studies [30]. Previous SRs on SR automation illustrated the challenge of developing search strategies to identify relevant research articles in the field. The large number of SRs published on various information retrieval, text mining, and AI applications makes it challenging to identify automated SRs, due to the large overlap in the terminology of these articles.

Due to the lack of specific search terms for articles on SR automation, the use of general terms such as “automated SR” carries the risk of low sensitivity, illustrated by the study of Dinter et al. [29], which, despite including automation studies in all stages of the SR workflow and extending the electronic search with a manual snowball technique, yielded fewer reports than earlier reviews focusing on a more specific aspect of SR automation [28]. On the other hand, the risk of low specificity was demonstrated by the review of Adbelkader et al., which aimed to identify a special, yet clinically relevant subset of review automation use-cases [30]. Altogether, the growing interest in automated SRs in medicine, and the somewhat diverse coverage of the field by SRs, warranted a scoping review of automated SRs.

By combining the search strategies of previous reviews, the objective of this study was to provide a comprehensive overview of the scope of SR automation across various stages of the SR workflow, as well as the adoption of automation techniques in published SRs among studies indexed in PubMed. Hence, we included both studies on SR automation methods (SSAM), and automated SRs (ASRs) (i.e., studies that used automation techniques when answering a primary research question unrelated to SR automation). Our research question referred to what SR stages were automated and what were the goals, the applied tools and methods, the data sources, and the key results of SR automation. We also performed a citation analysis to assess the research impact of SR automation studies (i.e., the extent to which their results were referenced by academic researchers).

## Methods

We followed the PRISMA-Scr reporting standard for scoping reviews [25]. The protocol for this study was not registered in advance.

### Automated systematic reviews

To define SRs, we used the general criteria proposed by Krnic-Martinic et al. [31]. As such, SRs feature a well-defined research question, a reproducible search strategy, clear inclusion, and exclusion criteria for relevant publications, reproducible selection and screening methods, critical appraisal of the quality or risk of bias for included studies, and reproducible data analysis or synthesis methods [31]. Throughout the review process, we considered as an SR automation tool any method that aims to speed up, assist, or replace manual reviewer tasks that require human judgement with an algorithm-based solution, while aiming to yield comparable results achievable by human reviewers. Papers reporting on tools that can potentially assist the SR workflow but are not developed or applied specifically for this purpose were excluded.

### Inclusion and exclusion criteria

Using the definitions above, we included full-text English peer-reviewed articles of both SSAMs and ASRs with no limit on publication date.

We excluded bibliographic analyses, or text-based knowledge discovery studies or information retrieval studies from large corpora. These studies employ advanced analytical methods to generate new results, rather than reducing the workload for tasks that humans can achieve. Furthermore, we excluded narrative reviews and nonautomated SRs on SR automation or SR automation tools or methods.

### Search strategy

We focused on published research in the medical field, so we limited our search on PubMed. The search was run on November 12, 2022. We extracted the search strategies of four published SRs on SR automation [22, 28–30], identified during the planning of this review (Additional file 1). The four strategies were combined into a single search syntax using the Boolean “OR” operator. We also run the four searches individually to count duplicate records. Abdelkader et al. narrowed down their general search strategy by using terms that refer to the quality of the articles [30]. For our search, these terms were removed to achieve higher sensitivity. Furthermore, we replaced the “mp (multipurpose)” Ovid Medline field with “Title/Abstract” in our PubMed search. The search syntax is provided in Additional file 2.

### Screening and selection of studies

Screening of titles and abstracts was completed independently by three single researchers (BT, LB, ZZ) on the combined record set. Uncertain items were discussed. Full-text papers were then evaluated by two independent reviewers against the inclusion and exclusion criteria (BT, ZZ). In case of disagreement or if reviewers were not sure whether an article was suitable for inclusion, they discussed its eligibility, and a joint decision was made.

### Data extraction

Two reviewers (BT, ZZ) extracted data from each eligible article using a predesigned spreadsheet. A senior reviewer (ZZ) compared and consolidated the extracted items. These encompassed publication meta-data, including details such as the first author’s name, publication year, article title, and the PubMed ID (PMID) for each article. Additionally, we collected information about the article type, categorizing them as either SSAMs or ASRs. Furthermore, we identified the SR stage where automation was applied, such as search, record screening, full-text selection, data extraction, risk of bias assessment, evidence synthesis, assessment of evidence quality, and reporting. Assigning automation methods to the appropriate SR stages was challenging due to the diversity of approaches. In Table 1, we provide positive examples illustrating our decisions to categorize automation methods within specific automated SR stages, as well as negative examples showing instances when a method was excluded or categorized elsewhere among the automated SR stages. We considered only the laborious execution parts of the SR workflow, omitting the steps of the review planning phase [16]. We also extracted details about the input text used, including the title, abstract, full text, or metadata. In addition, we gathered information about the text representation methods employed, which ranged from basic techniques such as bag-of-words or term frequency to more advanced methods such as vector representation and large language models. Moreover, if reported, we recorded the best performing machine learning models or algorithms used for text classification and task learning. We took note of the accessible corpora used for learning or testing, along with their weblinks if provided in the studies. Additionally, we recorded information about off-the-shelf or freeware automation software utilized in the studies, including any available weblinks. We noted if multiple packages were used from a single software environment (i.e., R, Python) without detailing the individual tools. Furthermore, we documented notable methodological details that had potential impact on results, such as experimentation with different feature sets or addressing feature imbalance. Finally, we noted key results related to performance metrics, including recall (sensitivity), precision (positive predictive value), workload-saving, time-saving, or any other significant metrics as reported by the authors.

**Table 1:** Categorization of the SR stages, where automation was applied with positive and negative examples

**Automated SR stage**	**Positive examples (i.e., automation methods categorized within the corresponding SR stage)**	**Negative examples (i.e., methods excluded or categorized elsewhere)**
Search	• Improving a search strategy (e.g., identifying relevant keywords to improve sensitivity and specificity) • Automating the construction of a search syntax	• Converting of a search strategy from one database to another • The setting up of search notifications for a living review • Deduplication of records
Record screening	• Deciding on the potential eligibility of an article based on its title, abstract, or keywords (e.g., ranking, classification, matching text against inclusion criteria) • Updating the SR with new potential articles using machine learning on annotated records from previous versions of the SR	• Tools that only support the transparency of the screening process or facilitate the communication between reviewers (e.g., review management software)
Full-text selection	• Using full-text information to decide on the eligibility of an article based on any method	• Methods that predict full-text eligibility without using information from the full-text report
Data extraction	• Identifying/extracting data in an eligible article that is relevant to answering the research question (e.g., images, tables, effect sizes)	• Extracting certain elements from the abstract/article to facilitate record screening, full-text selection, or the assessment of risk-of bias or reporting quality
Risk of bias assessment	• Assessment of methodological adequacy, the presence/absence of methodological safeguards	• Manual completion of risk of bias questionnaires
Evidence synthesis	• Automating the selection and/or application of quantitative evidence synthesis methods (e.g., meta-analysis)	• Standard statistical procedures, that replace manual computations • Novel descriptive or explorative or knowledge discovery methods (e.g., mapping, topic modeling, networking, visualization, trend analysis) • Bibliographic analyses
Assessment of evidence quality	• Automating the assessment about the certainty/confidence in the evidence supporting the findings	• Risk of bias or reporting quality assessments
Reporting	• E.g., textual summarization of results	• Graphical representation of novel evidence synthesis methods (e.g., topic models, networks) • Graphs or tables from traditional evidence summaries (e.g., meta-analysis, funnel plot)

As a proxy of potential research impact, we added the number of Google Scholar (GS) citations of the included studies, collected on 16th July 2023. Finally, from ASRs, we extracted the research aims, the number of records and included studies, key results, the automated SR stage, and the applied SR automation tools and their reported performance.

### Data synthesis

We analyzed data via descriptive methods. We counted the number of eligible papers on automation methods and automated systematic reviews by publication year, and by the SR stage, and reported time savings by each automated SR stage. We also tabulated the key characteristics of ASRs, and if reported, we calculated workload saved on screening from the proportion of records screened using automated tools, assuming that screening replaced manual work. If not reported otherwise, we assumed that manual tasks were performed by a single reviewer.

## Results

### Results of the literature search

**Fig. 1:**
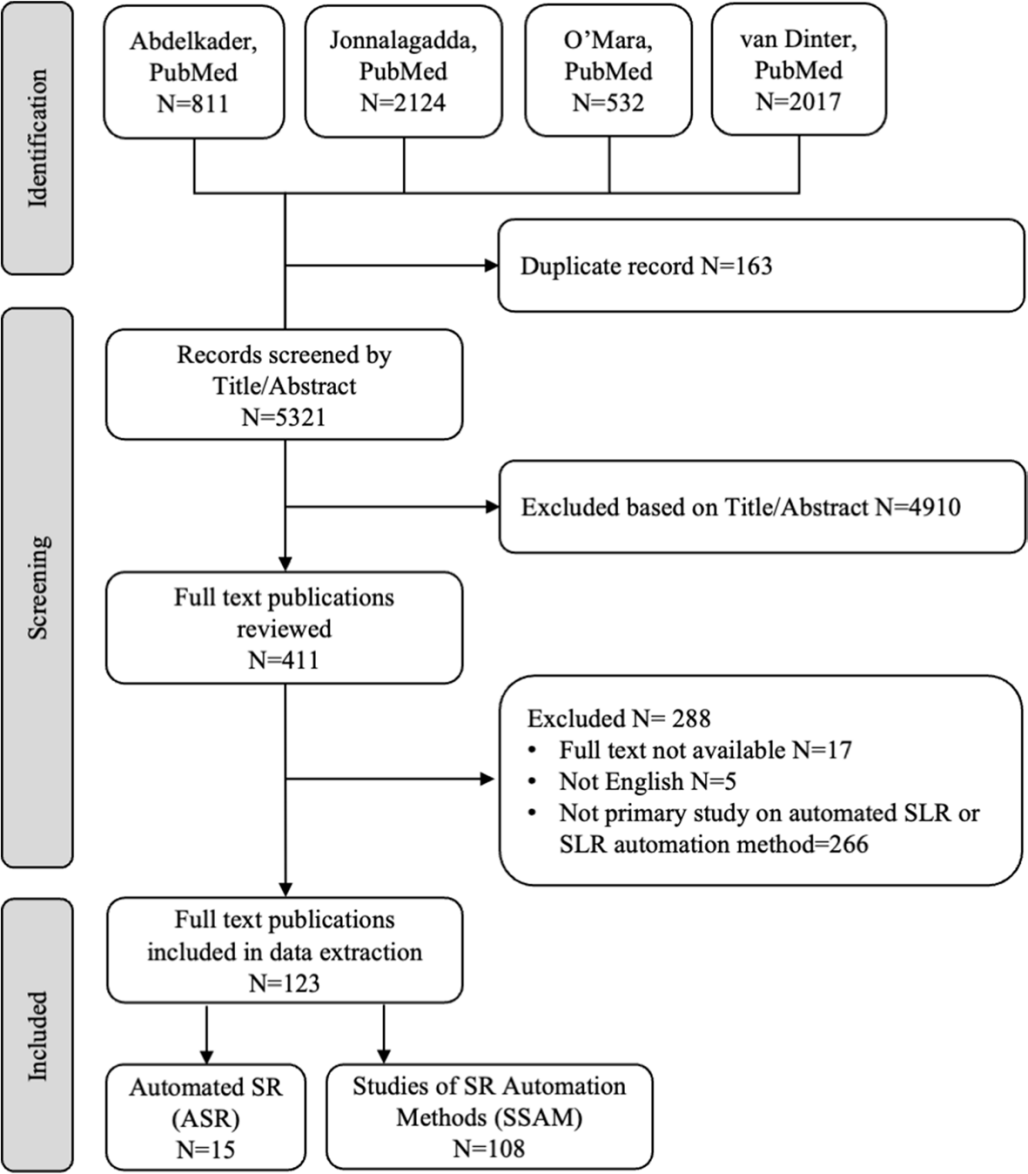
PRISMA flowchart of selected reviews

**Fig. 2:**
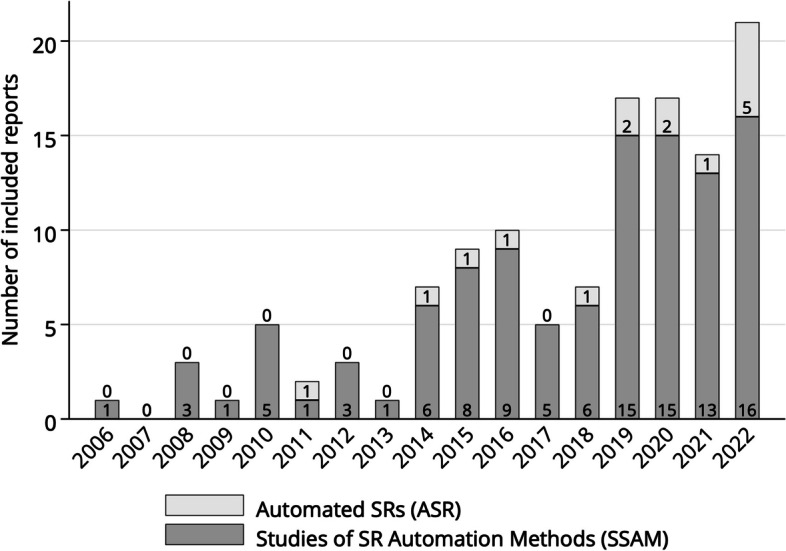
Distribution of articles by publication year

The four search strategies yielded 5484 hits, with only 163 duplicate records (3.0%), suggesting minimal overlap between previous SRs on SR automation. The combined search yielded 5321 results, out of which 411 potential eligible records were sent to full text screening. A further 288 articles were excluded during full text screening for various reasons (Additional file 3). Finally, 123 articles were included (Figs. 1 and 2). We found 15 ASR studies (12.2%), and 108 papers reporting SSAMs (87.8%). The extracted data from all included studies are summarized in Additional file 4.

### Characteristics of the included studies

#### Date of publication

**Fig. 3:**
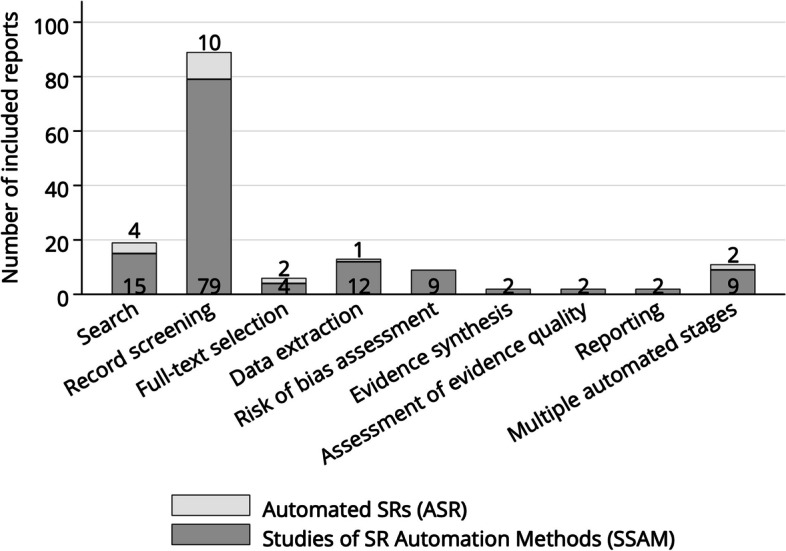
Number of articles by automated stage of the systematic literature review (SR) process. *Articles with automation of multiple SR stages were counted at each stage

The first included paper was published in 2006. It investigated whether automation could reduce the SR workload. The study suggested that 20–50% time could be saved with a 95% recall level during abstract screening by using a bag of words model and a voting perceptron machine learning classifier [32]. Since 2014, the number of studies increased rapidly with 56.1% (69/123) of included papers published from 2019 onwards. We found automation examples for all stages of the SR workflow (Fig. 3).

#### Search

Nineteen included papers (15.4%) aimed to automate or improve database searches [18, 33–50]. The first included paper from 2011 applied text-mining to construct a search syntax for PubMed, using the Apache Lucene platform [33]. Eleven papers used a plethora of text-mining tools to aid search syntax building, such as Anne O’Tate, AntConc, Apache Lucene, BiblioShiny, Carrot2, CitNetExplorer, EndNote, Keyword‐Analyzer, Leximancer, Lingo3G, Lingo4G, MeSH on Demand, MetaMap, Microsoft Academic, PubReMiner, Systematic Review Accelerator, TerMine, Text Analyzer, Tm for R, VOSviewer, Voyant, Yale MeSH Analyzer, and in-house solutions [18, 33–35, 37, 41, 46, 47, 49–51]. Two papers introduced curated article collections, such as Cochrane CENTRAL [44], and the Realtime Data Synthesis and Analysis (REDASA) COVID-19 dataset [48], which were assembled using various automation techniques. Other tools included an automated extension of PubMed searches to the ClinicalTrials.gov database [40], a Boolean query refiner [42], a support vector machine (SVM) classifier as alternative to PubMed search filters for review updating [38], a strategy using the Patient, Intervention, Comparator, and Outcome framework (PICO) terms in the title field only [39], an automated full-text retrieval and targeted search replacing database screening [45], and a Microsoft Excel-based convenience tool to build Boolean queries [43].

#### Record screening

The most popular SR automation approach was record screening based on titles and abstracts (*N* = 89, 72.4%). Within this approach, automated classification (*N* = 32/89, 36.0%) was the most frequently reported strategy [32, 52–82]. In automatic classification, a subset of manually screened records is used to train a machine learning classifier, which proposes records that should undergo manual full-text selection. The second most prevalent strategy was active learning (*N* = 24/89, 27.0%) [83–106]. In active learning, a small seed group of relevant records is used for initial training. Records are manually screened by the order of relevance predicted by the model. Using the results, the model is periodically retrained until finding relevant records becomes unlikely. In the third most used strategy, review updates, all included papers and excluded records of a published review are used for training, and the aim is to predict the inclusion of a record from new search results in the updated review (*N* = 12/89, 13.5%) [107–118]. The priority ranking strategy (*N* = 10/89, 11.2%) [119–128] was used least often. This strategy predicts the priority of records after single training round. By screening relevant records early, subsequent phases of the SR can advance faster. Other studies applied a combination of strategies [41, 129], used alternative methods such as filtering [18], or similarity of Medline elements [130], reported the automation software without detailing the strategy [131–133], used convenience tools to speed up screening [134, 135], or omitted record screening and applied topic modeling directly to full-text selection [45].

SVM was by far the most prevalent machine learning method, usually used in ensemble models (*N* = 24/89, 27.0%) [52, 53, 59, 60, 66, 68, 70, 72, 83–87, 89, 91, 92, 96, 106, 108, 116, 119–121, 129], followed by naïve Bayes (*N* = 7, 7.9%) [54–57, 74, 116, 133], and logistic regression (*N* = 7, 7.9%) [58, 61, 70, 93, 95, 112, 114]. More recent developments included the use of similarity-based metrics [88, 109, 115, 130], and advanced neural networks, including a feed-forward neural network [69], bidirectional long-short-term memory network (BiLSTM) [93, 100], deep learning [102], and networks integrated in large language models (e.g., bidirectional encoder representations for transformers, BERT) [71, 79]. Studies in which the machine learning model was not specified (*N* = 30/89, 33.7%) often reported the use of off-the shelf automation software (*N* = 27/89, 30.3%).

As an input to machine learning models, most often bag-of-words (BOW) text representations were applied (*N* = 30/89, 33.7%) [32, 41, 52, 54–56, 59, 61, 68, 72, 82, 84, 85, 87, 89, 92, 93, 95, 96, 100, 106, 108, 110, 112, 114, 115, 119–122], followed by term-frequency/inverse document frequency (TF-IDF) (*N* = 16/89, 18.0%) [45, 53, 57, 60, 63, 66, 68, 73, 76, 83, 91, 109, 115, 116, 122, 123], topic models (*N* = 10/89, 11.2%) [45, 60, 84, 86, 91, 93, 104, 107, 109, 115, 123], keywords (*N* = 9, 10.1%) [52, 75, 76, 91, 98, 100, 117, 123, 127], standardized terms such as Medical Subject Headings (MeSH) (*N* = 6/89, 6.7%) [59, 61, 76, 88, 119, 123], or semantic annotation to the Unified Medical Language System (UMLS) (*N* = 6 /89, 6.7%) [55, 63, 83, 88, 104, 119], named entity recognition [74, 79, 93], various word or document vector representations (*N* = 10, 11.2%) [41, 68, 70, 75, 86, 100–102, 104, 115], or various BERT models (*N* = 5, 5.6%) [69, 71, 79, 81, 118]. As raw input, most studies used PubMed records including title, abstract, MeSH terms, and in a few instances, bibliographic details. Few studies used full-text input (*N* = 5, 5.6%) [45, 63, 98, 125, 127] and database records from ClinicalTrials.gov or Cochrane (*N* = 4/89, 4.5%) [78, 79, 109, 115]. We note that some studies were conducted on published SR databases, such as the EPPI Centre database [60, 83, 84, 86, 89] or those from the Oregon Drug Effectiveness Review Project (DERP) [32, 41, 54, 68, 77, 87, 88, 93, 107, 120–122, 130]. Links to public SR resources were extracted and provided in Additional file 4.

The off-the shelf or freeware screening automation software were Abstrackr [90, 94, 97, 98, 105, 113, 132], EPPI Reviewer [80, 128, 132, 136], RobotAnalyst [91, 94, 113, 131], Distiller SR [94, 99, 126], Rayyan [103, 131], Systematic Review Accelerator [18, 135], RCT Tagger [77, 78], SWIFT Review [125, 126], SyRF [92, 111], ASR (automated systematic review) [67], ASReview [133], Aggregator [58], ATCER [63], Cochrane RCT Classifier [72], Covidence [131], Curious Snake [83], DoCTER [65], GAP Screener [52], MetaPreg [74], Research Screener [102], revtools [134], RobotAnalyst, and TeMMPo [124]. The detailed description of these tools is beyond the scope of this study. The weblinks to these tools were extracted from the references and are provided in Additional file 4.

The great variety of applied automation strategies, reported performance metrics, and applied datasets prevented a level performance comparison of automated record screening tools. A key observation is that, although the mean performance of automation tools improved over time, their performance varied greatly across different research topics covered by SRs. On 15 SRLSs of the Oregon DERP dataset, the mean workload saved over sampling at 95% recall (WSS@95) of automation tools increased from 23.4% in 2006 (range 0.31–70.5%) [32] through 33.5% in 2010 (range 8.5–62.5%) [54], 37.1% in 2015 (range 9.0–74.3%) [130], to 48.4% in 2016 (range 13.7–82.6%) [122] and 41.0% in 2017 (range 5.8–81.6%) [88]. On the same dataset, the WSS@95 of Rayyan was 49 ± 18% [87].

The variability of performance was illustrated by the post hoc analysis of results using a PICO-based term recognition strategy in study titles. The single keyword “Parkinson’s”, appearing in most records of a SR, deteriorated the specificity of the automated screener leading to only 11% workload savings. When omitting terms related to participants, the workload savings increased to 57% in the same dataset. In contrast, the original strategy yielded 78% workload savings in an SR focused on phenytoin use for seizure prophylaxis in brain injury [39].

The time saving achieved by automated record screening also varied. Based on the averted screenings and mean screening time per record, the median estimated time saving was 29.8 h per review (range 11.7–198 h) across 10 SRs, with a mean time saving of 32.5 s per record (range 18.1–43.5 s) [99]. Using a similar approach, another study reported median estimated time saving of 26 h across 16 SRs (range 9–42 h), with a mean time saving of 22.6 s per record in a subset of 10 SRs (range 9.6–27.0 s) [97]. Other studies reported 23.5 [67], 44.7 [98], and 61-, 64-, and 92-h [94] time savings per SR. In the study of Hamel et al., the estimated median time saving increased from 29.8 to 36 h when the averted workload of full-text selection was also considered [99]. Time savings were also affected by the learning curve of reviewers. In a SR involving 10,599 records, manual screening of all records took 61 h (20.7 s per record), while screening the first 1809 records to train the automation tool took 16.3 h (32.4 s per record). Measured by activity logging, the time savings per record were 15.2 s [98].

#### Full-text selection

Six papers (4.9%) focused on automated full-text selection. Most studies searched keywords using text-mining tools. The first paper, an ASR from 2016 [137], used Linux bash to search keywords in full-text PDF files. Another study comparing automation with duplicate human reviewers used QDA Miner [98]. An environmental health SR used the segmenteR R package to extract terms from specified article sections [127]. A large environmental health ASR used Distiller SR [126]. Two studies aiming to dramatically speed up the SR process applied a convenience tool for navigation and full-text management in a reference management software (Systematic Review Accelerator) [18, 135].

Time saving was reported in one study: 30.5 h were saved on the automated full-text selection of 555 articles (198 s per article) [98].

#### Data extraction

Thirteen studies (10.6%) involved an automated data extraction tool. The first paper published in 2010 introduced ExaCT, a rule-based tool to extract clinical trial characteristics [138]. The efficiency of ExaCT was prospectively compared with that of human reviewers, and showed modest time savings [139]. Further four papers applied text mining to create structured summaries of relevant pieces of information from full text documents. Out of these, three studies used in-house packages including UMLS semantic annotation [51], keyword search [127], and PICO entity recognition using BERT [81]. The fourth tool, developed for public health purposes, Dextr [140] combined vector embedding text representation and deep learning. Further approaches included PECO tagging in a rapid evidence mapping study using SWIFT Review [125], extraction of geographic locations from the manuscript [141], extraction of endpoints as comparative claim sentences [142], data extraction from ClinicalTrials.gov for meta-analyses [143], and convenience tools to highlight relevant sentences [74], or extract data from graphs [144]. Finally, development of the REDASA COVID-19 dataset involved human experts in the loop, web-crawling, and a natural language processing search engine to provide a real-time curated open dataset for evidence syntheses to aid pandemic response [48].

Using automated data extraction, the mean time savings per included study were 454 [140], 691 [139], and 1440 [143] s. The synthesized outcomes per study ranged between 5 [140] and 24 [143]. The time savings depended on the applied automation strategy. In a study by Gates et al. [139], when automated data extraction was used to expedite a second reviewer, the time savings were 3.7 h on a SR involving 75 studies. However, when automation replaced the second reviewer, the time saving increased to 14.4 h. The mean time savings were 352 s per graph when using a convenience data extraction tool [144].

#### Risk of bias assessment

Nine (7.3%) studies looked into the automation of risk of bias assessment. The first studies were published in early 2016 introducing RobotReviewer [145] and an alternative prototype tool, Systematic Review Assistant [146]. Both tools were trained on the Cochrane Database for Systematic Reviews. Following the Cochrane Risk of Bias (RoB1) tool for randomized controlled trials (RCTs), RobotReviewer provides an overall assessment of risk of bias, and extracts supporting sentences from PDF files of full-text reports [145]. RobotReviewer was used in an additional five studies [18, 135, 147–149]. One paper assessed the risk of bias in preclinical animal studies, comparing various techniques including recurrent neural networks with attention, convolutional neural networks, and BERT [150]. Tangentially related to risk of bias assessment, an environmental health study automatically ranked papers based on their data quality [127].

Using RobotReviewer, the mean time saving on automated risk of bias assessment per study was 69 s in 52 RCTs (755 vs 824 s) [147]. In another SRs, risk of bias assessment using seven domains of the Cochrane Collaboration’s RoB1 tool needed 23 h and 40 min for 16 studies (5340 s per study), while RobotReviewer finished in 2 h and 12 min assessing four risk of bias domains (495 s per study), saving 4845 s per study [135].

#### Evidence synthesis

We identified two papers on automated evidence synthesis, both published in 2022. One of them applied a full SR automation workbench involving automated data extraction followed by combined script for effect size calculation and meta-analysis (MetaPreg) [74]. The other paper introduced the DIAeT tool for generating qualitative evidence summary sentences from clinical trials [151].

#### Assessment of evidence quality

We identified two papers focusing on the automated assessment of evidence quality using a semi-automated quality assessment tool (SAQAT). SAQAT is based on a Bayesian network classifier that assigns probabilities to overall GRADE (Grades of Recommendation, Assessment, Development, and Evaluation) categories using a set of standardized questions. Both papers were published in 2015 [23, 24].

#### Reporting

We identified one study from 2022, where automated report generation was part of an integrated SR automation workflow using MetaPreg, an integrated SR automation platform focusing on medicines during pregnancy [74].

#### Automating multiple stages of the SR workflow

While most papers focused on a single SR stage, eleven studies (8.9%) automated multiple stages. Using the Systematic Review Accelerator, a team a team was able to complete the SR process within a 2-week timeframe by automating multiple SR stages including search, record screening, full-text selection, and risk of bias assessment [18, 135]. In one of these studies, time savings were documented versus a manual work. The SR involved 586 records and 16 studies. The full manual review took 126 h (out of which 25 h was spent on task learning), and automation was applied on SR stages taking 41 h and 33 min to complete (out of which learning time was 6 h 5 min). For the same SR stages, automation took 11 h and 48 min (including 1 h and 18 min for learning the tasks), saving 30 h, which amounted to 23.8% of the total completion time. Another team also automated multiple steps of the SR using MetaPreg and finished a SR in 14 days, saving 10.7 workdays compared to a conventional SR approach [74]. Others combined multiple open-access tools including SWIFT Review, R, and Python packages to automate the record screening, full-text selection, and data extraction of a SR on the toxic effects of nanomaterials [127]. Some studies combined two stages from either search, screening, full-text selection, or data extraction. These studies included two ASRs [45, 126], studies on alternative SR approaches, such as Rapid Evidence Mapping [125] and Potential Technologies Review [41], and the REDASA COVID-19 dataset [48]. A study used automated record screening before evaluating a text mining algorithm for full text selection [98], and another automated record screening in connection with PICO named entry recognition for data extraction [81].

#### Google Scholar citations

The average number of citations per article was 122.3 (range 0–9015, median 22). The most cited paper (published in 2016) introduced Rayyan, a leading SR platform (*N* = 9015) [87], followed by an ASR on mindfulness for smoking cessation (*N* = 526) [49], a study introducing Curious snake, a freeware active learning-based screening automation tool (*N* = 323) [83], the seminal study from Cohen et al., introducing an automated classifier tool and WSS@95, a key performance metric for screening automation (*N* = 320) [32], and an ASR on leptospirosis transmission (*N* = 304) [37]. Further nine SSAMs [18, 54, 84, 86, 122, 129, 134, 138, 145] and two ASRs [80, 136] received over 100 citations. From the nine highly cited SSAMs, four introduced automation tools, such as the revtools R package for screening [134], the SWIFT Review text mining tool [122], ExaCT for automatic extraction of clinical trial data [138], and RobotReviewer for automated assessment of risk of bias in clinical trials [145], and five reported methodological innovation, such as completing a SR in 2 weeks [18], reducing workload in extreme reviews with 1 million records [129], certainty-based screening in active learning [84], topic detection based on paragraph vectors in active learning [86], and an improved automated classification algorithm [54].

### Summary of automated systematic reviews

The topics of ASRs were usually broad, with on average 17,952 records (range 962–52,219) and 691 included studies (range 13–6305). From the 15 ASRs, four (26.7%) reviews automated the search [33, 37, 45, 49], eleven (73.3%) the screening [45, 67, 80, 100, 103, 110, 111, 126, 133, 136], two (13.3%) the full text selection [126, 137], and one (6.7%) the data extraction phase [141]. One study did not report the software [100], six used open source software [33, 37, 45, 110, 137, 141], and eight studies used off-the shelf tools [49, 67, 80, 103, 111, 126, 133, 136]. Three studies (20.0%) reported recall with values between 96% and 100% [67, 111, 126]. Workload saved on screening could be obtained from eight (53.3%) studies [45, 67, 100, 110, 111, 126, 136, 137] with values ranging between 31.7% and 100%. Some studies used automated screening to extend manual searches, thereby increasing the sensitivity of the reviews at the cost of minimal extra screening effort [67, 103]. Details of the ASR are provided in Table 2.

**Table 2:** Characteristics of automated SRs (*N* = 15)

**Author, year**	**Aim**	***N* of records**^**a**^**/included full text**	**Automation stage**	**Automation process**	**Automation Tool**	**Automation results (recall/WLS**^**b**^**)**
Oertelt-Prigione, 2011 [33]	Compare gender-related aspects of studies in stroke and myocardial infarction	962/405	Search	Text-mining was used to aid PubMed search. No further details were reported	Apache Lucene	Recall: na/WLS: na
Mytton, 2014 [136]	To identify qualitative studies on facilitators and barriers of engagement in parenting programs	12,249/26	Screening	Automatic term recognition was trained on 7246 citations screened by a single reviewer, and then applied on all records (*N* = 12,249, i/e: 444/11,805). After confirming eligibility, 37 citations were selected via automatic term recognition for full-text assessment.	EPPI-Reviewer 4	Recall: na/WLS: 37.2%
Mwachui, 2015 [37]	To synthesize quantitative evidence about environmental risk factors of leptospirosis transmission.	Original review 12,025/53 Updated review 229/13	Search	A review covering 1970–2008 was updated for 2008–2015. A Markov-Chain algorithm interactively built a search query by replacing common words and optimizing precision/recall.	[R]	Recall: na/WLS: na
Trypsteen, 2016 [137]	Map the use of droplet digital PCR (ddPCR) in HIV virus quantification.	2565/19	Full text selection	After database search, 2206 full text PDF files were collected, and searched for the presence of relevant keywords. The resulting 42 papers were manually examined for eligibility.	Linux Bash	Recall: na/WLS: 100% (manual screening was omitted)
Xiong, 2018 [110]	Meta-analysis on the relative risk of atrial fibrillation in diabetes mellitus.	4177/29	Screening	Search in title (*N* = 139, i/e: 26/113), manual selection of relevant seed studies. Then search in all fields (*N* = 4177), followed by K-means clustering and maximum entropy classification on similarity to seed studies. Records in most similar cluster (*N* = 416, i/e: 38/378) were manually screened. Studies for meta-analysis (*N* = 29) were selected manually. Manual screening in pairs (*N* = 4177, i/e: 45/4132) also yielded 29 studies for meta-analysis.	[R]	Recall: na/WLS: 87% Automation found 100% of included papers but recall of automated screening was not reported.
Currie, 2019 [111]	Systematic review and meta-analysis of chemotherapy-induced peripheral neuropathy (CIPN).	Original review 33,814/180 Updated review 11,880/157	Screening	Using the original review’s duplicate manual screening results as training set (*N* = 33,814, i/e: 6506/27,308), a machine learning (ML) classifier was run on records from updated search (*N* = 11,880). Model selection/evaluation was performed on randomly selected 10%/10% records screened manually in duplicate. The classifier with best precision at cut-off for 0.95 recall was selected. Then, relevant chemotherapy terms for CIPN were sought by text-mining in titles/abstracts (*N* = 6108, i/e: 928/5180) to select included records for full-text selection.	SyRF (retrieved from reference)	Recall: 97%/WLS: 80% Further 85% workload saving on full text selection due to text-mining:
Odintsova, 2019 [67]	A comprehensive overview of reviews on the genetics of human aggression, and primary genome-wide association studies (GWASs).	Reviews: 1686 + 13,572/18 + 4 GWASs: 356 + 13,572/17 + 3	Screening	Using a manually annotated dataset (*N* = 2955, i/e: 152/2803) the ASR software was trained on samples with different i/e ratios (*N* = 500). The model with greatest precision at recall ≤ 0.03 was applied to classify the retrieved records for reviews (*N* = 1713, i/e: 1081/695), GWAS studies (*N* = 356, i/e: 243/113), and records from a broad search (*N* = 13,572 after removing duplicates, i/e: 6469/7103).	ASR (automated systematic review)	Reviews: Recall: 100%/WLS: 31.7% GWASs: Recall: 96%/WLS: 31.7% Broad search: Recall: na/WLS: 31.7% Due to the common training set in the three searches, we calculate a single workload saving value for the entire study: The automated broad search additionally yielded 4 reviews and 3 GWAS studies not identified by manual searches. The authors reported 39.1% (23.5 h) time saving on screening.
Li, 2020 [100]	Review of satellite Earth observation (EO) or geographic information system (GIS) data in identifying landscape factors that affect dengue fever transmission.	7696/101	Screening	Records (*N* = 7696) were filtered using text scoring manual weights on pre-selected keywords to select initial training set (*N* = 2034), followed by active learning in 5 cycles, using an initial training dataset from text scoring (*N* = 45, i/e: 15/30). A word2vec continuous bag of words (CBOW) model with BiLSTM algorithm was used (deep active learning). All records designated as potentially relevant were screened manually (*N* = 1056, i/e: 131/925). In consecutive training cycles, relevant records were combined with randomly selected irrelevant records from text scoring in 1:2 ratio, until all records were classified.	na	Recall: na/WLS: 85.7% Recall of the entire automation process was not evaluated. However, recall of active learning was 100% in 1056 manually screened records. No relevant records were found manually among the 925 records classified as irrelevant by the algorithm.
Thiabaud, 2020 [45]	To review the sociobehavioral factors influencing HIV prevalence and incidence in Malawi.	16,942/27	Full text selection	Pdf files were automatically retrieved after search (*N* = 22,709, i/e: 16,942/5767), pre-processed, and analyzed via topic modeling (625 topics). Titles and abstracts of full-text papers in the 14 relevant topics were screened manually (*N* = 519, i/e: 119/400). From 119 selected full-text papers, 20 were eligible. Additional 7 papers were identified among the references of included papers.	[Python]	Recall: na/WLS: 93.2% Topic review was added to reviewer-burden: The 519 potentially relevant papers were identified in 5 days. Recall was not evaluated.
Gaskins, 2021 [103]	To review from professional (healthcare, exercise and fitness) staff perspective the factors affecting the implementation of aerobic exercise after stroke.	11,449/20	Screening	Screening was completed manually by pairs of reviewers (*N* = 11,449, i/e: 331/11,118). Rayyan was trained on manual results, and 200 most relevant records were screened manually by a single reviewer (i/e: 162/38). Records were re-screened manually (*N* = 493, i/e: 63/434). 63 full-text papers were assessed for eligibility (i/e: 20/43).	Rayyan	Recall: na/WLS: na Automation improved the credibility at the cost of extra work in this study. The proportion of studies identified solely by automation in the final set of included studies was not reported. The number of full text records checked for eligibility increased by 6.8% (4/59) at the cost of increasing screening burden by 3%.
Carlson, 2022 [126]	Prepare a systematic evidence map for per- and polyfluoroalkyl substances (PFAS).	52,219/339	Search Screening Full text selection	(Two reviews’ results are combined: Nafion + 150 PFAS) Database search yielded 52,219 records after deduplication (Nafion PubMed syntax was automatically created by SWIFT Review “Find Chemical Synonyms”). 16,378 records were de-prioritized and removed. Records relevant to human health were selected by SWIFT-Review Evidence Streams (*N* = 35,841, i/e: 15,414/20,427). Relevant PFAS records were screened by title/abstract via active learning to meet population, exposure, comparator, outcome (PECO) criteria using SWIFT-Review Active Screener, with cut-off set at 0.95 recall (*N* = 13,161, i/e: 1483/11,678). In active screening, 5390 records were screened manually. Additional records from grey sources were added, and records were screened using Distiller SR via keyword tagging in title / abstract (*n* = 5267, i/e: 981/4286) followed by full text selection (*n* = 981, i/e: 339 / 642).	SWIFT review, Distiller SR	For SWIFT-review active screener: Recall: 96%/WLS: 59.0% When excluding the de-prioritized records, for Nafion + PFAS, the estimated screening workload saving with SWIFT-Review Evidence Streams, Active Screener, and Distiller SR was 84.9% The title/abstract screening was completed in 94 working hours.
De Menezes, 2022 [141]	Geographical distribution of gender-related topics in arboviral vector control literature.	7367/2812	Data extraction	After manual search, geographic locations were extracted from 2812 records (title, abstract).	[R]	Recall: na/WLS: na
Jackson, 2022 [49]	To evaluate the efficacy of mindfulness-based interventions for smoking cessation among smokers.	2900/55	Search	Conventional database search yielded 3557 records. 112 records were added from an automated search in Microsoft Academic using a search strategy from the Human Behaviour Change Database. After deduplication, 2900 records were processed in a manual review.	Microsoft Academic	Recall: na/WLS: na Automation increased the credibility of the review by increasing the number of records by 3.1%. The number of included papers identified solely by the automated search was not reported.
van Lissa, 2022 [133]	A text-mining systematic review of phenomena relevant to adolescent emotion regulation.	6584/6305	Screening	A search string was manually constructed from keywords to retrieve relevant seed records (*N* = 29, retrieved: 25, missed: 4). From 6584 records after deduplication, 559 were screened by Rayyan (i/e: 367/192), followed by screening 541 records in ASReview (i/e: 456/85). Missed records were added, 6305 papers were suitable for text mining (out of scope).	Rayyan, ASReview	Recall: na/WLS: na
Viner, 2022 [80]	To review the association of school closures with mental health, health behaviors, and well-being in children and adolescents during COVID lockdown.	16,817/36	Screening	From 16,817 records, the authors screened 1500 to train a ML classifier to rank records by relevance. Records with relevance score above threshold were screened by two independent authors (title/abstract). A single reviewer also screened records with lower relevance (title only). Altogether, 151 records were reviewed in full text.	EPPI-reviewer 4	Recall: na/WLS: na No details were reported on the applied threshold, recall, or efficiency of the automated screening.

^a^Number of records after removing duplicates

^b^*WLS* Workload saved on screening (assuming that manual tasks were performed by a single reviewer unless tasks performed by two independent reviewers are explicitly reported in the manuscript)

## Discussion

We provided a comprehensive overview of SR automation studies across all stages of the SR workflow, featuring a detailed catalogue of 123 articles indexed in PubMed and published until November 2022. The number of papers and available tools has shown rapid growth over time. Automation tools were developed for all stages of the SR workflow, with majority of research (72%) focusing on the record screening phase. Most included articles (88%) were SSAMs with only 12% ASRs, suggesting that the uptake of SR automation tools in real practice is still in its infancy. The use of automated search, screening, full text selection, and data extraction was demonstrated in published ASRs, even in combination [126].

It has been demonstrated that an integrated automation workflow over multiple SR stages can lead to savings in reviewer effort and expedite the SR process [18, 74, 135]. While some integrated SR automation toolkits are available [18, 74, 135], most available tools can automate only a single SR stage, with potentially limited impact on the entire review process. Even when employing automation on multiple SR stages, the time savings compared to the total review process duration remained modest [135]. It is difficult to predict what are the effects of SR automation on the entire review. The performance of automation tools varies largely across review topics [32, 39, 54, 122, 130]. Achievable time savings depend on various factors, including the extent to which automation replaces human reviewers [139], the impact of automating one SR stage on the workload of subsequent review tasks [99], the baseline speed of the manual reviewer team [135, 147], the complexity of the research question [140, 143], the learning curve of reviewers [98], and the overall size of the review (i.e., the number of records and eligible articles). We note that some studies reported time savings based on actual measurements, while others relied on estimates. In general, the little detail was provided about the measurement methods of time savings. Moreover, the diverse automation strategies, datasets, and performance metrics complicate the assessment of the utility of available tools. Altogether, standardized reporting practices and evaluation metrics would be helpful to keep track of the progress in SR automation. The frequently incomplete reporting of automation performance in ASRs also calls for better reporting standards.

Workload savings via automated record screening may come at the cost of imperfect sensitivity, which has been shown to impact the results of meta-analyses [97]. The consequences of reduced sensitivity may vary between SRs and should be carefully considered on a case-by-case basis. However, automation can increase the sensitivity of SRs, when applied in addition to manual screening. In some ASRs, extending manual work with automated record screening increased the sensitivity of SRs with minimal extra effort [67, 103].

The citation analysis provided insights into the most impactful research articles concerning SR automation. While the introduction of an off-the self SR management tool was the most cited paper in this review [87], some highly cited papers indicated considerable interest about open-source tools [83, 134], multiple stages of automation including screening [83, 134], text mining [122], data extraction [138], and risk of bias assessment [145]. Solutions enabling extreme performance, such as completing a SR in 2 weeks [18] or the screening of 1 million records [129], were also frequently cited.

Compared to existing reviews in SR automation, our review has unique features. Although the SR automation toolbox, an online inventory of SR automation tools, provides a comprehensive collection of available solutions [152], our review also covered methods in development and published SRs using automation techniques. By combining the search syntaxes of four published reviews in the field, the coverage of our study was broader than reviews focusing on specific aspects of SR automation, including a review of text-mining for study identification (*N* = 44) [22], data extraction (*N* = 26) [28], retrieval of high-quality clinical studies (*N* = 10) [30], SR software packages including those with automation features [153, 154], reviews using AI-based automation (*N* = 12) [155], a living review of automated data extraction tools (*N* = 53) [156], or the syntheses of workload reduction via automated screening (*N* = 21 and *N* = 86) [27, 157]. Some reviews aimed for full coverage of SR automation. Van Dinter et al. [29] identified 41 studies, while a recent scoping review on the use of AI in biomedical literature analyses covered 273 research articles, although with broader focus including the assembly of evidence (*N* = 127), literature mining (*N* = 112), and quality analysis (*N* = 34) [158].

Automation or semi-automation of record screening was the most active area of research covered by several systematic reviews. A review of 44 studies reported WSS@95 values between 30% and 70% [22]. A meta-analysis of 15 studies reported WSS at maximal recall levels in a range of − 0.3% to 89.7%. Mean recall was 92.8% (95% CI 87.8–95.8%) in this sample [157]. A recent meta-analysis of 21 studies reported mean WSS@95 of 55% (95% CI 51–58%) [27]. Similar to our findings, the authors commented on diverse reporting practices, and the scarcity of direct comparative studies on automation tools [22, 27]. While considerable workload savings are achievable, consistent performance at high recall levels is still elusive, leaving human screening indispensable [157].

The low overlap between the search results of previous SRs on SR automation underscore the challenges associated with identifying relevant research in this field. These challenges arise due to the blurred boundaries between SR automation and more general approaches in medical information management. For example, the seminal article by Aphinyanaphongs from 2005 [159], which is considered by many authors as the inaugural paper for automated record screening, was excluded during our record screening due to the lack of specific reference to systematic reviews. Conversely, we excluded many papers on methods with potential applicability for systematic reviews, but without testing their performance in a systematic review context. Furthermore, some web-based SR tools with automation features were not captured by our search (e.g., Nested Knowledge) [160]. Standardized terminology, performance criteria, evaluation methods, and reporting of SR automation research papers would help the scientific community to keep track of the developments and make informed decisions about the adoption of SR automation tools. At the meeting point of medicine and computer science, the consolidation of terminology, definitions, and reporting standards seems to be a general challenge including digital health [161] or medical AI research [162].

The breadth and depth of our review, the coverage of both methodological development and the application of automation methods, and unique elements, such as citation analysis, are strengths of our review. However, our research has limitations. The search was restricted to PubMed, the main resource for biomedical literature. However, relevant papers indexed elsewhere may have been missed. The four SRs from which search syntaxes were combined were identified informally, so some relevant syntaxes may have been missed from our combined search syntax. Also, although uncertain items were discussed, some records may have been lost in the screening by single reviewers. Furthermore, some decisions about the eligibility of certain papers were challenging, and relied on personal judgements, despite the predefined inclusion and exclusion criteria. The same applies to our judgements during data extraction, when characterizing the sometimes abundant and complex methodological details of studies. However, the accidentally omitted records or methodological details would not alter the overall findings of our review. Furthermore, the citation analysis could not differentiate whether the citations referred to the general review management or review automation use case of some tools (e.g., Rayyan). While our review focused on SRs of biomedical literature, we assume that findings about the applied technologies and focus of research may be generalized to automated SRs in scientific fields outside medicine.

## Conclusions

While record screening is the most active area of research, automation tools are being developed for all stages of the SR workflow (i.e., search, record screening, full-text selection, data extraction, risk of bias assessment, evidence synthesis, assessment of evidence quality, and reporting) and have been shown to save reviewer effort or expedite the SR process. However, the real world adoption of SR automation techniques is still limited. The performance (i.e., sensitivity and specificity) of automation techniques varies largely between SRs, and it is difficult to predict their ultimate benefit in real world applications. Most tools are available for the automation of a single SR stage, while the potential time savings compared to the entire review process are modest even if multiple stages or the SR workflow are automated. Standardized terminology, reporting practices, and evaluation metrics would enhance the real-life adoption of SR automation practices. Given the increasing demand for evidence syntheses in medical research and medical decision-making, it is important that more researchers become familiar with the use of SR automation techniques, and experience accumulates over a greater evidence base. Until the benefits and risks of SR automation are better understood, automation tools could be used more often in parallel with manual reviews. Complementing manual reviews with automation techniques could facilitate the developments in the field, with potentially increasing the sensitivity or quality of published SRs with acceptable extra reviewer effort.

### Supplementary Information

Additional file 1. Characteristics of identified SRs on SR automation.

Additional file 2. Search strategy (Search date: Nov 12th, 2022).

Additional file 3. Excluded records in full-text selection.

Additional file 4. Details of included studies.

## Data Availability

All data used for this research is provided in the additional files.

## Abbreviations

AI: Artificial intelligence
ASR: Automated systematic literature review
BERT: Bidirectional encoder representations for transformers
BiLSTM: Bidirectional long-short-term memory network
BOW: Bag of words
CBOW: Continuous bag of words
CIPN: Chemotherapy-induced peripheral neuropathy
DERP: Drug effectiveness review project
EO: Earth observation
GIS: Geographic information system
GRADE: Grades of Recommendation, Assessment, Development, and Evaluation
GS: Google Scholar
GWAS: Genome-wide association studies
MeSH: Medical Subject Headings
ML: Machine learning
NA: Not available
PECO: Population, exposure, comparator, and outcome
PFAS: Polyfluoroalkyl substances
PICO: Patient, intervention, comparator, and outcome
RCT: Randomized clinical trial
RoB: Risk of bias
REDASA: Real-time data synthesis and analysis
SAQAT: Semi-automated quality assessment tool
SR: Systematic literature review
SSAM: Studies on SR automation methods
SVM: Support vector machine
TF-IDF: Term-frequency/inverse document frequency
UMLS: Unified Medical Language System
WLS: Workload saved
WSS@95: Workload saved over sampling at 95% recall
