# Supplementary material for: Automation of systematic reviews of biomedical literature: a scoping review of studies indexed in PubMed
Source: Syst Rev. 2024 Jul 8;13:174. doi: 10.1186/s13643-024-02592-3 (PMC11229257; doi:10.1186/s13643-024-02592-3)
Supplement: Supplementary file 1 — Additional file 1. Characteristics of identified SRs on SR automation. [file 13643_2024_2592_MOESM1_ESM.docx]

Additional file 1. Characteristics of identified SLRs on SLR automation

| **Author, year** | **Title** | **Focused  SLR stage** | **Period covered** | **No. of found studies** | **No. Of included studies** |
| --- | --- | --- | --- | --- | --- |
| Abdelkader, 2021 (30) | Machine Learning Approaches to Retrieve High-Quality, Clinically Relevant Evidence From the Biomedical Literature: Systematic Review | identifying relevant high quality studies | Until July 8, 2020 | 3632 | 10 |
| van Dinter, 2021 (29) | Automation of systematic literature reviews: A systematic literature review | Any stage | Jan 2000 – June, 2020 | 1291 | 41 |
| Jonnalagadda, 2015 (28) | Automating data extraction in systematic reviews: A systematic review | data extraction | Jan 1, 2000 – Jan 6, 2015 | 1225 | 26 |
| O’Mara-Eves, 2015 (22) | Using text mining for study identification in systematic reviews: a systematic review of current approaches | identifying relevant studies | Jan 2005 – Dec, 2013 | 1253 | 44 |
