# Supplementary material for: Automation of systematic reviews of biomedical literature: a scoping review of studies indexed in PubMed
Source: Syst Rev. 2024 Jul 8;13:174. doi: 10.1186/s13643-024-02592-3 (PMC11229257; doi:10.1186/s13643-024-02592-3)
Supplement: Supplementary file 2 — Additional file 2. Search strategy (Search date: Nov 12th, 2022). [file 13643_2024_2592_MOESM2_ESM.docx]

**Additional file 2. Search strategy (Search date: Nov 12th, 2022)**

| Author | Search | Syntax | Hits |
| --- | --- | --- | --- |
| Abdelkader [16] | #1 | ("neural networks" [Title/Abstract] or "machine learning" [Title/Abstract] or "natural language processing" [Title/Abstract] or "data mining" [Title/Abstract] or "support vector machine"[Title/Abstract] or "text categorization" [Title/Abstract] or "text classification" [Title/Abstract] or "text analysis" [Title/Abstract] or "literature mining" [Title/Abstract] or "text mining" [Title/Abstract]) AND ("Abstracting and Indexing" [Title/Abstract] or "information storage and retrieval" [Title/Abstract] or "article retrieval" [Title/Abstract] or "literature surveillance" [Title/Abstract] or "literature screening" [Title/Abstract] or "article screening" [Title/Abstract] or "evidence search" [Title/Abstract] or "evidence screening" [Title/Abstract] or "evidence review" [Title/Abstract] or "information retrieval" [Title/Abstract] or "literature survey" [Title/Abstract] or "document classification" [Title/Abstract] or "review efficiency" [Title/Abstract] or "citation screening" [Title/Abstract] or "literature databases" [Title/Abstract]) AND ("1900/01/01"[Date - Publication] : "2022/11/12"[Date - Publication]) | 811 |
| Jonnalagdda [17] | #2 | (“identification” [Title] OR “extraction” [Title] OR “extracting” [Title] OR “detection” [Title] OR “identifying” [Title] OR “summarization” [Title] OR “learning approach” [Title] OR “automatically” [Title] OR “summarization” [Title] OR “identify sections” [Title] OR “learning algorithms” [Title] OR “Interpreting” [Title] OR “Inferring” [Title] OR “Finding” [Title] OR “classification” [Title]) AND (“medical evidence”[Title] OR “PICO”[Title] OR “PECODR” [Title] OR “intervention arms” [Title] OR “experimental methods” [Title] OR “study design parameters” [Title] OR “Patient oriented Evidence” [Title] OR “eligibility criteria” [Title] OR “clinical trial characteristics” [Title] OR “evidence based medicine” [Title] OR “clinically important elements” [Title] OR “evidence based practice” [Title] “results from clinical trials” [Title] OR “statistical analyses” [Title] OR “research results” [Title] OR “clinical evidence” [Title] OR “Meta Analysis” [Title] OR “Clinical Research” [Title] OR “medical abstracts” [Title] OR “clinical trial literature” [Title] OR ”clinical trial characteristics” [Title] OR “clinical trial protocols” [Title] OR “clinical practice guidelines” [Title]) AND ("1900/01/01"[Date - Publication] : "2022/11/12"[Date - Publication]) | 2124 |
| O’Mara [18] | #3 | ("text mining"[Title/Abstract] OR "literature mining"[Title/Abstract] OR "machine learning"[Title/Abstract] OR "machine-learning"[Title/Abstract] OR "automation"[Title/Abstract] OR "semi-automation"[Title/Abstract] OR "semi-automated"[Title/Abstract] OR "automated"[Title/Abstract] OR "automating"[Title/Abstract] OR "text classification"[Title/Abstract] OR "text classifier"[Title/Abstract] OR "text categorization"[Title/Abstract] OR "text categorizer"[Title/Abstract] OR (classify*[Title/Abstract] AND text [Title/Abstract]) OR "support vector machine"[Title/Abstract] OR SVM[Title/Abstract] OR "Natural Language Processing"[Title/Abstract] OR "active learning"[Title/Abstract] OR "text clusters"[Title/Abstract] OR "text clustering"[Title/Abstract] OR "clustering tool"[Title/Abstract] OR "text analysis"[Title/Abstract] OR "textual analysis"[Title/Abstract] OR "data mining"[Title/Abstract] OR "term recognition"[Title/Abstract] OR "word frequency analysis"[Title/Abstract]) AND ("systematic review*"[Title/Abstract] OR "article retrieval" "document retrieval"[Title/Abstract] OR "citation retrieval"[Title/Abstract] OR "retrieval task"[Title/Abstract] OR (identify*[Title/Abstract] AND articles[Title/Abstract]) OR (identify*[Title/Abstract] AND citations[Title/Abstract]) OR (identify*[Title/Abstract] AND documents[Title/Abstract]) OR "citation screening"[Title/Abstract] AND "document screening"[Title/Abstract] OR "article screening"[Title/Abstract] OR "citation management"[Title/Abstract] OR "review management"[Title/Abstract] OR "evidence synthesis"[Title/Abstract] OR "research synthesis"[Title/Abstract] OR "evidence review"[Title/Abstract] OR "research review"[Title/Abstract] OR "comprehensive review"[Title/Abstract] OR "reference scanning"[Title/Abstract] OR "reference scanning"[Title/Abstract]) AND ("1900/01/01"[Date - Publication] : "2022/11/12"[Date - Publication]) | 532 |
| van Dinter [19] | #4 | (“Automation” OR “Automating” OR “Automated” OR “Automatic” OR “Automates” OR “Mining”) AND (“Systematic review” OR “Systematic Literature Review”) AND ("1900/01/01"[Date - Publication] : "2022/11/12"[Date - Publication]) | 2017 |
| This study | #5 | #1 OR #2 OR #3 OR #4 | 5321 |
