# Supplementary material for: Automation of systematic reviews of biomedical literature: a scoping review of studies indexed in PubMed
Source: Syst Rev. 2024 Jul 8;13:174. doi: 10.1186/s13643-024-02592-3 (PMC11229257; doi:10.1186/s13643-024-02592-3)
Supplement: Supplementary file 3 — Additional file 3. Excluded records in full-text selection. [file 13643_2024_2592_MOESM3_ESM.docx]

**Additional file 3. Excluded records in full-text selection**

| **Author, year** | **Title** | **PMID** | **Reason for exclusion** |
| --- | --- | --- | --- |
| Haynes, 1998 | Using informatics principles and tools to harness research evidence for patient care: evidence-based informatics | 10384550 | Full text not available |
| Rodrigues, 2000 | Information systems: the key to evidence-based health practice | 11143195 | Not primary study on SR automation method or automated SR |
| Costa-Bouzas, 2001 | HEpiMA: software for the identification of heterogeneity in meta-analysis | 11137192 | Not primary study on SR automation method or automated SR |
| Fuchs, 2001 | The human olfactory subgenome: from sequence to structure and evolution | 11214901 | Not primary study on SR automation method or automated SR |
| Liu, 2003 | Mining terminological knowledge in large biomedical corpora | 12603046 | Not primary study on SR automation method or automated SR |
| Kostoff, 2004 | Information content in Medline record fields | 15171980 | Not primary study on SR automation method or automated SR |
| Muller, 2004 | Textpresso: an ontology-based information retrieval and extraction system for biological literature | 15383839 | Not primary study on SR automation method or automated SR |
| Bernstam, 2005 | Using citation data to improve retrieval from MEDLINE | 16221938 | Not primary study on SR automation method or automated SR |
| Hamerlynck, 2005 | [From the Cochrane Library: brushing the teeth with an electric toothbrush with an oscillating rotating movement more effective against plaque and gingivitis than brushing with a conventional toothbrush] | 16358617 | Not primary study on SR automation method or automated SR |
| Lee, 2005 | A systematic review of user interface issues related to PDA-based decision support systems in health care | 16779308 | Not primary study on SR automation method or automated SR |
| Rubinstein, 2005 | MILANO--custom annotation of microarray results using automatic literature searches | 15661078 | Not primary study on SR automation method or automated SR |
| Aphinyanaphongs, 2006 | A comparison of citation metrics to machine learning filters for the identification of high quality MEDLINE documents | 16622165 | Not primary study on SR automation method or automated SR |
| Jung, 2006 | Identifying differentially expressed genes in meta-analysis via Bayesian model-based clustering | 16845907 | Full text not available |
| Lin, 2006 | "Bag of words" is not enough for strength of evidence classification | 16779318 | Not primary study on SR automation method or automated SR |
| Wren, 2006 | A scalable machine-learning approach to recognize chemical names within large text databases | 17118146 | Not primary study on SR automation method or automated SR |
| Hammamieh, 2007 | GeneCite: a stand-alone open source tool for high-throughput literature and pathway mining | 17594234 | Not primary study on SR automation method or automated SR |
| Hettne, 2007 | Applied information retrieval and multidisciplinary research: new mechanistic hypotheses in complex regional pain syndrome | 17480215 | Not primary study on SR automation method or automated SR |
| Shrank, 2007 | Effect of content and format of prescription drug labels on readability, understanding, and medication use: a systematic review | 17426075 | Not primary study on SR automation method or automated SR |
| Theodosiou, 2007 | Non-linear correlation of content and metadata information extracted from biomedical article datasets | 17643352 | Not primary study on SR automation method or automated SR |
| Torabinejad, 2007 | Outcomes of root canal treatment and restoration, implant-supported single crowns, fixed partial dentures, and extraction without replacement: a systematic review | 17936128 | Not primary study on SR automation method or automated SR |
| Azuaje, 2008 | Discovering relevance knowledge in data: a growing cell structures approach | 18252376 | Not primary study on SR automation method or automated SR |
| Meystre, 2008 | Extracting information from textual documents in the electronic health record: a review of recent research | 18660887 | Not primary study on SR automation method or automated SR |
| Chung, 2009 | Sentence retrieval for abstracts of randomized controlled trials | 19208256 | Not primary study on SR automation method or automated SR |
| El Emam, 2009 | The use of electronic data capture tools in clinical trials: Web-survey of 259 Canadian trials | 19275984 | Not primary study on SR automation method or automated SR |
| Kostoff, 2009 | Seminal nanotechnology literature: a review | 19908521 | Not primary study on SR automation method or automated SR |
| Krallinger, 2009 | Extraction of human kinase mutations from literature, databases and genotyping studies | 19758464 | Not primary study on SR automation method or automated SR |
| Ponomarenko, 2009 | [Identification of differentially expressed proteins using automatic meta-analysis of proteomics-related articles] | 19351029 | Full text not available |
| Spasic, 2009 | KiPar, a tool for systematic information retrieval regarding parameters for kinetic modelling of yeast metabolic pathways | 19336445 | Not primary study on SR automation method or automated SR |
| Torabinejad, 2009 | Outcomes of nonsurgical retreatment and endodontic surgery: a systematic review | 19567310 | Not primary study on SR automation method or automated SR |
| Yeniterzi, 2009 | EnzyMiner: automatic identification of protein level mutations and their impact on target enzymes from PubMed abstracts | 19758466 | Not primary study on SR automation method or automated SR |
| Castillo, 2010 | A knowledge-based taxonomy of critical factors for adopting electronic health record systems by physicians: a systematic literature review | 20950458 | Not primary study on SR automation method or automated SR |
| Chapman, 2010 | Semi-automating the manual literature search for systematic reviews increases efficiency | 20402801 | Not primary study on SR automation method or automated SR |
| Jahiruddin, 2010 | A concept-driven biomedical knowledge extraction and visualization framework for conceptualization of text corpora | 20870033 | Not primary study on SR automation method or automated SR |
| Matos, 2010 | Concept-based query expansion for retrieving gene related publications from MEDLINE | 20426836 | Not primary study on SR automation method or automated SR |
| Stanfill, 2010 | A systematic literature review of automated clinical coding and classification systems | 20962126 | Full text not available |
| Cohen, 2011 | Performance of support-vector-machine-based classification on 15 systematic review topics evaluated with the WSS@95 measure | 21169622 | Not primary study on SR automation method or automated SR |
| Dias, 2011 | Whole field tendencies in transcranial magnetic stimulation: A systematic review with data and text mining | 23051076 | Not primary study on SR automation method or automated SR |
| Lin, 2011 | Automating the process of critical appraisal and assessing the strength of evidence with information extraction technology | 21707873 | Not primary study on SR automation method or automated SR |
| Cohen, 2012 | Studying the potential impact of automated document classification on scheduling a systematic review update | 22515596 | Not primary study on SR automation method or automated SR |
| Karakulah, 2012 | A data-driven living review for pharmacogenomic decision support in cancer treatment | 22874279 | Not primary study on SR automation method or automated SR |
| Korhonen, 2012 | Text mining for literature review and knowledge discovery in cancer risk assessment and research | 22511921 | Not primary study on SR automation method or automated SR |
| Lefebvre, 2013 | Methodological developments in searching for studies for systematic reviews: past, present and future? | 24066664 | Not primary study on SR automation method or automated SR |
| Sgourakis, 2013 | Endoscopic and surgical resection of T1a/T1b esophageal neoplasms: a systematic review | 23539431 | Not primary study on SR automation method or automated SR |
| Stansfield, 2013 | 'Clustering' documents automatically to support scoping reviews of research: a case study | 26053843 | Full text not available |
| Whetzel, 2013 | NCBO Technology: Powering semantically aware applications | 23734708 | Not primary study on SR automation method or automated SR |
| Ahmed, 2014 | Structured Correspondence Topic Models for Mining Captioned Figures in Biological Literature | 25485170 | Not primary study on SR automation method or automated SR |
| Baxter, 2014 | Using logic model methods in systematic review synthesis: describing complex pathways in referral management interventions | 24885751 | Not primary study on SR automation method or automated SR |
| Boeckmann, 2014 | Is planned adaptation to heat reducing heat-related mortality and illness? A systematic review | 25349109 | Not primary study on SR automation method or automated SR |
| Hausner, 2014 | Development of search strategies for systematic reviews: validation showed the noninferiority of the objective approach | 25464826 | Not primary study on SR automation method or automated SR |
| Li, 2014 | Learning by doing-teaching systematic review methods in 8 weeks | 26052850 | Not primary study on SR automation method or automated SR |
| McBride, 2014 | Understanding human management of automation errors | 25383042 | Not primary study on SR automation method or automated SR |
| Mullins, 2014 | Reporting quality of search methods in systematic reviews of HIV behavioral interventions (2000-2010): are the searches clearly explained, systematic and reproducible? | 26052651 | Not primary study on SR automation method or automated SR |
| Petersen, 2014 | Increased workload for systematic review literature searches of diagnostic tests compared with treatments: challenges and opportunities | 25600450 | Not primary study on SR automation method or automated SR |
| Tsafnat, 2014 | Systematic review automation technologies | 25005128 | Not primary study on SR automation method or automated SR |
| Zhou, 2014 | Citations alone were enough to predict favorable conclusions in reviews of neuraminidase inhibitors | 25450452 | Not primary study on SR automation method or automated SR |
| Baer, 2015 | Can Natural Language Processing Improve the Efficiency of Vaccine Adverse Event Report Review? | 26394725 | Not primary study on SR automation method or automated SR |
| Bayliss, 2015 | Information retrieval for ecological syntheses | 26099482 | Not primary study on SR automation method or automated SR |
| Hartling, 2015 | EPC Methods: An Exploration of Methods and Context for the Production of Rapid Reviews | 25654160 | Not primary study on SR automation method or automated SR |
| Hersant, 2015 | Current indications of low-level laser therapy in plastic surgery: a review | 25954831 | Not primary study on SR automation method or automated SR |
| Jonnalagadda, 2015 | Automating data extraction in systematic reviews: a systematic review | 26073888 | Not primary study on SR automation method or automated SR |
| Kamalov, 2015 | Improving data retrieval quality: Evidence based medicine perspective | 26639684 | Full text not available |
| Kim, 2015 | State of the field: An informatics-based systematic review of the SOD1-G93A amyotrophic lateral sclerosis transgenic mouse model | 25998063 | Not primary study on SR automation method or automated SR |
| Lazarou, 2015 | Ultrasound-guided synovial biopsy: a systematic review according to the OMERACT filter and recommendations for minimal reporting standards in clinical studies | 26022188 | Not primary study on SR automation method or automated SR |
| Li, 2015 | [Diseases treated by moxibustion and fire needling in clinical practice based on data mining: a comparison study] | 25675571 | Not English |
| O'Mara-Eves, 2015 | Using text mining for study identification in systematic reviews: a systematic review of current approaches | 25588314 | Not primary study on SR automation method or automated SR |
| O'Mara-Eves, 2015 | Erratum to: Using text mining for study identification in systematic reviews: a systematic review of current approaches | 25927201 | Not primary study on SR automation method or automated SR |
| Taggar, 2015 | Accuracy of methods for diagnosing atrial fibrillation using 12-lead ECG: A systematic review and meta-analysis | 25705010 | Not primary study on SR automation method or automated SR |
| Torabinejad, 2015 | Survival of Intentionally Replanted Teeth and Implant-supported Single Crowns: A Systematic Review | 25742795 | Not primary study on SR automation method or automated SR |
| Zhao, 2015 | [Analysis on medication regularity of modern traditional Chinese medicines in treating melancholia based on data mining technology] | 26390670 | Not English |
| Hausner, 2016 | Prospective comparison of search strategies for systematic reviews: an objective approach yielded higher sensitivity than a conceptual one | 27256930 | Not primary study on SR automation method or automated SR |
| Kivlehan, 2016 | Cardiac Resuscitation RESEARCH REVIEW. Studies address the efficacy of continuous chest conpressions and automated cornmpression devices | 26946587 | Not primary study on SR automation method or automated SR |
| Lopprich, 2016 | Automated Classification of Selected Data Elements from Free-text Diagnostic Reports for Clinical Research | 27406024 | Full text not available |
| Paynter, 2016 | EPC Methods: An Exploration of the Use of Text-Mining Software in Systematic Reviews | 27195359 | Not primary study on SR automation method or automated SR |
| Rastegar-Mojarad, 2016 | BELTracker: evidence sentence retrieval for BEL statements | 27173525 | Not primary study on SR automation method or automated SR |
| Reed, 2016 | The HCV care continuum among people who use drugs: protocol for a systematic review and meta-analysis | 27401499 | Not primary study on SR automation method or automated SR |
| Robson, 2016 | Studies in using a universal exchange and inference language for evidence based medicine. Semi-automated learning and reasoning for PICO methodology, systematic review, and environmental epidemiology | 27846446 | Not primary study on SR automation method or automated SR |
| Schweigel, 2016 | Salivary and pellicle proteome: A datamining analysis | 27966577 | Not primary study on SR automation method or automated SR |
| Shemilt, 2016 | Use of cost-effectiveness analysis to compare the efficiency of study identification methods in systematic reviews | 27535658 | Not primary study on SR automation method or automated SR |
| Uttley, 2016 | Building the Evidence Base of Blood-Based Biomarkers for Early Detection of Cancer: A Rapid Systematic Mapping Review | 27426280 | Not primary study on SR automation method or automated SR |
| Xu, 2016 | Extracting genetic alteration information for personalized cancer therapy from ClinicalTrials.gov | 27013523 | Not primary study on SR automation method or automated SR |
| Afzal, 2017 | Smart Extraction and Analysis System for Clinical Research | 27782787 | Full text not available |
| Bashir, 2017 | A systematic review of the processes used to link clinical trial registrations to their published results | 28669351 | Not primary study on SR automation method or automated SR |
| Iwarsson, 2017 | Analysis of cell-free fetal DNA in maternal blood for detection of trisomy 21, 18 and 13 in a general pregnant population and in a high risk population - a systematic review and meta-analysis | 27779757 | Not primary study on SR automation method or automated SR |
| Kattadiyil, 2017 | Clinical complications and quality assessments with computer-engineered complete dentures: A systematic review | 28222878 | Not primary study on SR automation method or automated SR |
| Kavakiotis, 2017 | Machine Learning and Data Mining Methods in Diabetes Research | 28138367 | Not primary study on SR automation method or automated SR |
| Kim, 2017 | DisArticle: a web server for SVM-based discrimination of articles on traditional medicine | 28129750 | Not primary study on SR automation method or automated SR |
| Marshall, 2017 | Automating Biomedical Evidence Synthesis: RobotReviewer | 29093610 | Not primary study on SR automation method or automated SR |
| Michie, 2017 | The Human Behaviour-Change Project: harnessing the power of artificial intelligence and machine learning for evidence synthesis and interpretation | 29047393 | Not primary study on SR automation method or automated SR |
| Olofsson, 2017 | Can abstract screening workload be reduced using text mining? User experiences of the tool Rayyan | 28374510 | Not primary study on SR automation method or automated SR |
| Olorisade, 2017 | Reproducibility of studies on text mining for citation screening in systematic reviews: Evaluation and checklist | 28711679 | Not primary study on SR automation method or automated SR |
| Schmidt, 2017 | SEED: a tool for disseminating systematic review data into Wikipedia | 29041959 | Not primary study on SR automation method or automated SR |
| Shokraneh, 2017 | Increasing value and reducing waste in data extraction for systematic reviews: tracking data in data extraction forms | 28778216 | Not primary study on SR automation method or automated SR |
| Skipper, 2017 | The hearing ear is always found close to the speaking tongue: Review of the role of the motor system in speech perception | 27821280 | Not primary study on SR automation method or automated SR |
| Stansfield, 2017 | Text mining for search term development in systematic reviewing: A discussion of some methods and challenges | 28660680 | Not primary study on SR automation method or automated SR |
| Thomas, 2017 | Living systematic reviews: 2. Combining human and machine effort | 28912003 | Not primary study on SR automation method or automated SR |
| Vlietstra, 2017 | Automated extraction of potential migraine biomarkers using a semantic graph | 28579531 | Not primary study on SR automation method or automated SR |
| Adroher, 2018 | All metrics are equal, but some metrics are more equal than others: A systematic search and review on the use of the term 'metric' | 29509813 | Not primary study on SR automation method or automated SR |
| Alla, 2018 | Can automated content analysis be used to assess and improve the use of evidence in mental health policy? A systematic review | 30442191 | Not primary study on SR automation method or automated SR |
| Anderson, 2018 | Systematic reviews and tech mining: A methodological comparison with case study | 30129708 | Full text not available |
| Bashir, 2018 | Software engineering principles address current problems in the systematic review ecosystem | 30582972 | Not primary study on SR automation method or automated SR |
| Beller, 2018 | Making progress with the automation of systematic reviews: principles of the International Collaboration for the Automation of Systematic Reviews (ICASR) | 29778096 | Not primary study on SR automation method or automated SR |
| Chen, 2018 | A bibliometric analysis of natural language processing in medical research | 29589569 | Not primary study on SR automation method or automated SR |
| de Souza, 2018 | A survey on Barrett's esophagus analysis using machine learning | 29626734 | Not primary study on SR automation method or automated SR |
| Del Fiol, 2018 | A Deep Learning Method to Automatically Identify Reports of Scientifically Rigorous Clinical Research from the Biomedical Literature: Comparative Analytic Study | 29941415 | Not primary study on SR automation method or automated SR |
| Dotto, 2018 | HTT-DB: new features and updates | 29315358 | Not primary study on SR automation method or automated SR |
| Hirt, 2018 | [Publications German-speaking countries in high impact journals: development and validation of a search filter] | 30547713 | Not English |
| Kreiner, 2018 | Twister: A Tool for Reducing Screening Time in Systematic Literature Reviews | 30306896 | Full text not available |
| Lee, 2018 | Comprehensive review of Hepatitis B Virus-associated hepatocellular carcinoma research through text mining and big data analytics | 30105774 | Not primary study on SR automation method or automated SR |
| Martinez-Garcia, 2018 | A systematic approach to analyze the social determinants of cardiovascular disease | 29370200 | Not primary study on SR automation method or automated SR |
| Musy, 2018 | Trigger Tool-Based Automated Adverse Event Detection in Electronic Health Records: Systematic Review | 29848467 | Not primary study on SR automation method or automated SR |
| Norman, 2018 | Data Extraction and Synthesis in Systematic Reviews of Diagnostic Test Accuracy: A Corpus for Automating and Evaluating the Process | 30815124 | Not primary study on SR automation method or automated SR |
| O'Connor, 2018 | Moving toward the automation of the systematic review process: a summary of discussions at the second meeting of International Collaboration for the Automation of Systematic Reviews (ICASR) | 29316980 | Not primary study on SR automation method or automated SR |
| O'Connor, 2018 | The study design elements employed by researchers in preclinical animal experiments from two research domains and implications for automation of systematic reviews | 29953471 | Not primary study on SR automation method or automated SR |
| Park, 2018 | Evidence synthesis software | 29880698 | Not primary study on SR automation method or automated SR |
| Pham, 2018 | Improving the conduct of systematic reviews: a process mining perspective | 30297037 | Not primary study on SR automation method or automated SR |
| Quesada-Martinez, 2018 | Towards the semantic enrichment of Computer Interpretable Guidelines: a method for the identification of relevant ontological terms | 30815135 | Not primary study on SR automation method or automated SR |
| Reboussin, 2018 | Systematic Review for the 2017 ACC/AHA/AAPA/ABC/ACPM/AGS/APhA/ASH/ASPC/NMA/PCNA Guideline for the Prevention, Detection, Evaluation, and Management of High Blood Pressure in Adults: A Report of the American College of Cardiology/American Heart Association Task Force on Clinical Practice Guidelines | 29146534 | Not primary study on SR automation method or automated SR |
| Ruano, 2018 | Evaluating characteristics of PROSPERO records as predictors of eventual publication of non-Cochrane systematic reviews: a meta-epidemiological study protocol | 29523200 | Not primary study on SR automation method or automated SR |
| Scarton, 2018 | Expanding vocabularies for complementary and alternative medicine therapies | 30545491 | Not primary study on SR automation method or automated SR |
| Schwebel, 2018 | Using text message reminders in health care services: A narrative literature review | 30206523 | Not primary study on SR automation method or automated SR |
| Shen, 2018 | DLAD4U: deriving and prioritizing disease lists from PubMed literature | 30591010 | Not primary study on SR automation method or automated SR |
| Waffenschmidt, 2018 | Effective study selection using text mining or a single-screening approach: a study protocol | 30340633 | Not primary study on SR automation method or automated SR |
| Wang, 2018 | Effect of acupuncture on in vitro fertilization: An updated systematic review and data mining protocol | 29901590 | Not primary study on SR automation method or automated SR |
| Ye, 2018 | Algorithms used to identify ventricular arrhythmias and sudden cardiac death in retrospective studies: a systematic literature review | 29224509 | Not primary study on SR automation method or automated SR |
| Bashir, 2019 | The risk of conclusion change in systematic review updates can be estimated by learning from a database of published examples | 30849512 | Not primary study on SR automation method or automated SR |
| Braithwaite, 2019 | Towards sustainable healthcare system performance in the 21st century in high-income countries: a protocol for a systematic review of the grey literature | 30782754 | Not primary study on SR automation method or automated SR |
| Brassey, 2019 | Developing a fully automated evidence synthesis tool for identifying, assessing and collating the evidence | 31467247 | Not primary study on SR automation method or automated SR |
| Crisan, 2019 | Adjutant: an R-based tool to support topic discovery for systematic and literature reviews | 30875428 | Not primary study on SR automation method or automated SR |
| Datta, 2019 | A frame semantic overview of NLP-based information extraction for cancer-related EHR notes | 31589927 | Not primary study on SR automation method or automated SR |
| Galetsi, 2019 | Values, challenges and future directions of big data analytics in healthcare: A systematic review | 31585681 | Not primary study on SR automation method or automated SR |
| Gennaro, 2019 | A systematic review of psychotherapy research topics (2000-2016): a computer-assisted approach | 32913819 | Not primary study on SR automation method or automated SR |
| Han, 2019 | Topic modeling of major research themes in disease ecology of mammals | 31138950 | Not primary study on SR automation method or automated SR |
| Hassanzadeh, 2019 | Quantifying semantic similarity of clinical evidence in the biomedical literature to facilitate related evidence synthesis | 31676460 | Not primary study on SR automation method or automated SR |
| Hossain, 2019 | Real-time crash prediction models: State-of-the-art, design pathways and ubiquitous requirements | 30634160 | Not primary study on SR automation method or automated SR |
| Lau, 2019 | Editorial: Systematic review automation thematic series | 30857554 | Not primary study on SR automation method or automated SR |
| Lin, 2019 | Acupuncture for fecal incontinence: Protocol for a systematic review and data mining | 30762773 | Not primary study on SR automation method or automated SR |
| Loskot, 2019 | Comprehensive Review of Models and Methods for Inferences in Bio-Chemical Reaction Networks | 31258548 | Not primary study on SR automation method or automated SR |
| Marshall, 2019 | Toward systematic review automation: a practical guide to using machine learning tools in research synthesis | 31296265 | Not primary study on SR automation method or automated SR |
| Michelson, 2019 | The significant cost of systematic reviews and meta-analyses: A call for greater involvement of machine learning to assess the promise of clinical trials | 31497675 | Not primary study on SR automation method or automated SR |
| O'Connor, 2019 | A question of trust: can we build an evidence base to gain trust in systematic review automation technologies? | 31215463 | Not primary study on SR automation method or automated SR |
| O'Connor, 2019 | Still moving toward automation of the systematic review process: a summary of discussions at the third meeting of the International Collaboration for Automation of Systematic Reviews (ICASR) | 30786933 | Not primary study on SR automation method or automated SR |
| Park, 2019 | Capturing the trend of mHealth research using text mining | 31728383 | Not primary study on SR automation method or automated SR |
| Rashid, 2019 | Bioavailability Enhancement of Poorly Soluble Drugs: The Holy Grail in Pharma Industry | 30706801 | Full text not available |
| Ryo, 2019 | Machine learning with the hierarchy-of-hypotheses (HoH) approach discovers novel pattern in studies on biological invasions | 31219681 | Not primary study on SR automation method or automated SR |
| Schmidt, 2019 | Introducing RAPTOR: RevMan Parsing Tool for Reviewers | 31242929 | Not primary study on SR automation method or automated SR |
| Shokraneh, 2019 | Reducing waste and increasing value through embedded replicability and reproducibility in systematic review process and automation | 31026544 | Not primary study on SR automation method or automated SR |
| van Altena, 2019 | Usage of automation tools in systematic reviews | 30561081 | Not primary study on SR automation method or automated SR |
| van Royen, 2019 | Automated CT quantification methods for the assessment of interstitial lung disease in collagen vascular diseases: A systematic review | 30777211 | Not primary study on SR automation method or automated SR |
| Venske, 2019 | Meta-Analysis of the QTLome of Fusarium Head Blight Resistance in Bread Wheat: Refining the Current Puzzle | 31263469 | Not primary study on SR automation method or automated SR |
| Viguera-Guerra, 2019 | Evolution of international collaborative research efforts to develop non-Cochrane systematic reviews | 30811451 | Not primary study on SR automation method or automated SR |
| Wan, 2019 | A review on microelectrode recording selection of features for machine learning in deep brain stimulation surgery for Parkinson's disease | 30293864 | Not primary study on SR automation method or automated SR |
| Yeung, 2019 | Curcumin: Total-Scale Analysis of the Scientific Literature | 30970601 | Not primary study on SR automation method or automated SR |
| Zhao, 2019 | Is cell transplantation a reliable therapeutic strategy for spinal cord injury in clinical practice? A systematic review and meta-analysis from 22 clinical controlled trials | 30666481 | Not primary study on SR automation method or automated SR |
| Alfattni, 2020 | Extraction of temporal relations from clinical free text: A systematic review of current approaches | 32673788 | Not primary study on SR automation method or automated SR |
| Amezcua-Prieto, 2020 | Artificial intelligence and automation of systematic reviews in women's health | 32516150 | Not primary study on SR automation method or automated SR |
| Arevalo-Rodriguez, 2020 | Current methods for development of rapid reviews about diagnostic tests: an international survey | 32404051 | Not primary study on SR automation method or automated SR |
| Cabanac, 2020 | Scholarly literature mining with information retrieval and natural language processing: Preface | 33223580 | Not primary study on SR automation method or automated SR |
| De-Scheerder, 2020 | Evolution of Experimental Design and Research Techniques in HIV-1 Reservoir Studies: A Systematic Review | 32167504 | Not primary study on SR automation method or automated SR |
| Elmore, 2020 | Risk and Protective Factors in the COVID-19 Pandemic: A Rapid Evidence Map | 33330323 | Not primary study on SR automation method or automated SR |
| Feng, 2020 | Surgical hand rubbing versus surgical hand scrubbing: Systematic review and meta-analysis of efficacy | 32331847 | Not primary study on SR automation method or automated SR |
| Gilad, 2020 | Intraventricular Hemorrhage in Very Preterm Infants: A Comprehensive Review | 32751801 | Not primary study on SR automation method or automated SR |
| Guzik, 2020 | What Has Been Trending in the Research of Polyhydroxyalkanoates? A Systematic Review | 33014998 | Not primary study on SR automation method or automated SR |
| Hackl, 2020 | Trends in Clinical Information Systems Research in 2019 | 32823305 | Not primary study on SR automation method or automated SR |
| Hagan, 2020 | A systematic review and meta-analysis of attentional bias toward food in individuals with overweight and obesity | 32298701 | Not primary study on SR automation method or automated SR |
| Hu, 2020 | A bibliometric analysis and visualization of medical data mining research | 32481411 | Not primary study on SR automation method or automated SR |
| John, 2020 | The impact of the COVID-19 pandemic on self-harm and suicidal behaviour: update of living systematic review | 33604025 | Not primary study on SR automation method or automated SR |
| Kueper, 2020 | Artificial Intelligence and Primary Care Research: A Scoping Review | 32393561 | Not primary study on SR automation method or automated SR |
| Lee, 2020 | MMiDaS-AE: Multi-modal Missing Data aware Stacked Autoencoder for Biomedical Abstract Screening | 34308444 | Not primary study on SR automation method or automated SR |
| Marshall, 2020 | Trialstreamer: A living, automatically updated database of clinical trial reports | 32940710 | Not primary study on SR automation method or automated SR |
| Marshall, 2020 | Semi-Automated evidence synthesis in health psychology: current methods and future prospects | 31941434 | Not primary study on SR automation method or automated SR |
| Mersha, 2020 | Barriers and Facilitators of Adherence to Nicotine Replacement Therapy: A Systematic Review and Analysis Using the Capability, Opportunity, Motivation, and Behaviour (COM-B) Model | 33265956 | Not primary study on SR automation method or automated SR |
| Noel-Storr, 2020 | Citation screening using crowdsourcing and machine learning produced accurate results: Evaluation of Cochrane's modified Screen4Me service | 33007457 | Not primary study on SR automation method or automated SR |
| O'Connor, 2020 | A focus on cross-purpose tools, automated recognition of study design in multiple disciplines, and evaluation of automation tools: a summary of significant discussions at the fourth meeting of the International Collaboration for Automation of Systematic Reviews (ICASR) | 32366302 | Not primary study on SR automation method or automated SR |
| Penning de Vries, 2020 | Title, abstract, and keyword searching resulted in poor recovery of articles in systematic reviews of epidemiologic practice | 31982541 | Not primary study on SR automation method or automated SR |
| Pouliliou, 2020 | Current trends in cancer immunotherapy: a literature-mining analysis | 32556496 | Not primary study on SR automation method or automated SR |
| Qing, 2020 | [Analysis of acupoint selection of plaster therapy in treatment of stable chronic obstructive pulmonary disease based on data mining] | 33350194 | Not English |
| Sant'Anna, 2020 | Systematic review of descriptions of novel bacterial species: evaluation of the twenty-first century taxonomy through text mining | 32100698 | Not primary study on SR automation method or automated SR |
| Schmidt, 2020 | Data extraction methods for systematic review (semi)automation: A living review protocol | 32724560 | Not primary study on SR automation method or automated SR |
| Sellers, 2020 | A Systematic Review of Neuropsychiatric Symptoms and Functional Capacity in Huntington's Disease | 31466515 | Not primary study on SR automation method or automated SR |
| Vilmun, 2020 | Impact of adding breast density to breast cancer risk models: A systematic review | 32361308 | Not primary study on SR automation method or automated SR |
| Wang, 2020 | Error rates of human reviewers during abstract screening in systematic reviews | 31935267 | Not primary study on SR automation method or automated SR |
| Wang, 2020 | A Systematic Review on the Research Progress and Evolving Trends of Occupational Health and Safety Management: A Bibliometric Analysis of Mapping Knowledge Domains | 32300581 | Not primary study on SR automation method or automated SR |
| West, 2020 | An online Paper Authoring Tool (PAT) to improve reporting of, and synthesis of evidence from, trials in behavioral sciences | 32833486 | Not primary study on SR automation method or automated SR |
| Yan, 2020 | Is it always possible to complete a systematic review in 2 weeks? Further thoughts and considerations | 32619752 | Not primary study on SR automation method or automated SR |
| Abdelkader, 2021 | Machine Learning Approaches to Retrieve High-Quality, Clinically Relevant Evidence From the Biomedical Literature: Systematic Review | 34499041 | Not primary study on SR automation method or automated SR |
| Al-Yousif, 2021 | A systematic review of automated pre-processing, feature extraction and classification of cardiotocography | 33987454 | Not primary study on SR automation method or automated SR |
| Ancin-Murguzur, 2021 | causalizeR: a text mining algorithm to identify causal relationships in scientific literature | 34322328 | Not primary study on SR automation method or automated SR |
| Bahor, 2021 | Development and uptake of an online systematic review platform: the early years of the CAMARADES Systematic Review Facility (SyRF) | 35047698 | Not primary study on SR automation method or automated SR |
| Benita, 2021 | Human mobility behavior in COVID-19: A systematic literature review and bibliometric analysis | 35720981 | Not primary study on SR automation method or automated SR |
| Bordignon, 2021 | Dataset of search queries to map scientific publications to the UN sustainable development goals | 33537369 | Not primary study on SR automation method or automated SR |
| Bozada, 2021 | Sysrev: A FAIR Platform for Data Curation and Systematic Evidence Review | 34423285 | Not primary study on SR automation method or automated SR |
| Brscic, 2021 | Challenging suicide, burnout, and depression among veterinary practitioners and students: text mining and topics modelling analysis of the scientific literature | 34488757 | Not primary study on SR automation method or automated SR |
| Buchlak, 2021 | Machine learning applications to neuroimaging for glioma detection and classification: An artificial intelligence augmented systematic review | 34119265 | Not primary study on SR automation method or automated SR |
| Buchter, 2021 | Reporting of methods to prepare, pilot and perform data extraction in systematic reviews: analysis of a sample of 152 Cochrane and non-Cochrane reviews | 34742231 | Not primary study on SR automation method or automated SR |
| Carracedo, 2021 | Research lines on the impact of the COVID-19 pandemic on business. A text mining analysis | 34744212 | Not primary study on SR automation method or automated SR |
| Carrasco, 2021 | Patient Safety in Palliative and End-of-Life Care: A Text Mining Approach and Systematic Review of Definitions | 33267627 | Not primary study on SR automation method or automated SR |
| Casey, 2021 | A systematic review of natural language processing applied to radiology reports | 34082729 | Not primary study on SR automation method or automated SR |
| Castillo-Segura, 2021 | Text mining applications in psychiatry: a systematic literature review | 33581827 | Not primary study on SR automation method or automated SR |
| Chen, 2021 | Current status and quality of radiomic studies for predicting immunotherapy response and outcome in patients with non-small cell lung cancer: a systematic review and meta-analysis | 34402924 | Not primary study on SR automation method or automated SR |
| de Siqueira, 2021 | Artificial intelligence applied to support medical decisions for the automatic analysis of echocardiogram images: A systematic review | 34629153 | Not primary study on SR automation method or automated SR |
| Fang, 2021 | Recent Trends in Sedentary Time: A Systematic Literature Review | 34442106 | Not primary study on SR automation method or automated SR |
| Ferreira, 2021 | Automation of Article Selection Process in Systematic Reviews Through Artificial Neural Network Modeling and Machine Learning: Protocol for an Article Selection Model | 34128820 | Not primary study on SR automation method or automated SR |
| Fontenele, 2021 | Influence of metal artefact reduction tool on the detection of vertical root fractures involving teeth with intracanal materials in cone beam computed tomography images: A systematic review and meta-analysis | 34003491 | Not primary study on SR automation method or automated SR |
| Gates, 2021 | LOCATE: a prospective evaluation of the value of Leveraging Ongoing Citation Acquisition Techniques for living Evidence syntheses | 33875014 | Not primary study on SR automation method or automated SR |
| Griswold, 2021 | Personal protective equipment for reducing the risk of COVID-19 infection among health care workers involved in emergency trauma surgery during the pandemic: An umbrella review | 33433175 | Not primary study on SR automation method or automated SR |
| Griswold, 2021 | Personal protective equipment for reducing the risk of COVID-19 infection among healthcare workers involved in emergency trauma surgery during the pandemic: an umbrella review protocol | 33653763 | Not primary study on SR automation method or automated SR |
| Griswold, 2021 | Chest Computed Tomography for the Diagnosis of COVID-19 in Emergency Trauma Surgery Patients Who Require Urgent Care During the Pandemic: Protocol for an Umbrella Review | 33878019 | Not primary study on SR automation method or automated SR |
| Hamel, 2021 | Guidance for using artificial intelligence for title and abstract screening while conducting knowledge syntheses | 34930132 | Not primary study on SR automation method or automated SR |
| Helmick, 2021 | Applying text mining to identify relevant literature in food science: Cold denaturation as a case study | 34653257 | Full text not available |
| Huang, 2021 | Enrichment and detection method for the prognostic value of circulating tumor cells in ovarian cancer: A meta-analysis | 33674144 | Not primary study on SR automation method or automated SR |
| Hudon, 2021 | Use of Automated Thematic Annotations for Small Data Sets in a Psychotherapeutic Context: Systematic Review of Machine Learning Algorithms | 34677133 | Not primary study on SR automation method or automated SR |
| Khalil, 2021 | Tools to support the automation of systematic reviews: a scoping review | 34896236 | Not primary study on SR automation method or automated SR |
| Li, 2021 | The Efficacy of Cell-Assisted Lipotransfer Versus Conventional Lipotransfer in Breast Augmentation: A Systematic Review and Meta-Analysis | 33452543 | Not primary study on SR automation method or automated SR |
| Liu, 2021 | The effectiveness of herbal acupoint application for functional diarrhea: Protocol for a meta-analysis and data mining | 34964730 | Not primary study on SR automation method or automated SR |
| Liu, 2021 | The effectiveness of acupoint application of traditional Chinese medicine in treating primary dysmenorrhea: A protocol for meta-analysis and data mining | 34128904 | Not primary study on SR automation method or automated SR |
| Maniu, 2021 | Inflammatory Biomarkers in Febrile Seizure: A Comprehensive Bibliometric, Review and Visualization Analysis | 34439695 | Not primary study on SR automation method or automated SR |
| Mather, 2021 | Dysregulation of immune response in otitis media | 34404500 | Not primary study on SR automation method or automated SR |
| Menon, 2021 | Risk factors associated with quad bike crashes: a protocol for systematic review of observational studies | 33820787 | Not primary study on SR automation method or automated SR |
| Mikles, 2021 | Health information technology to support cancer survivorship care planning: A systematic review | 34333588 | Not primary study on SR automation method or automated SR |
| Mora, 2021 | Not everything is as it seems: Digital technology affordance, pandemic control, and the mediating role of sociomaterial arrangements | 36570778 | Not primary study on SR automation method or automated SR |
| Nama, 2021 | Successful incorporation of single reviewer assessments during systematic review screening: development and validation of sensitivity and work-saved of an algorithm that considers exclusion criteria and count | 33820560 | Not primary study on SR automation method or automated SR |
| Pocock, 2021 | Communication of poor prognosis between secondary and primary care: protocol for a systematic review with narrative synthesis | 34949630 | Not primary study on SR automation method or automated SR |
| Redsell, 2021 | Barriers and enablers to caregivers' responsive feeding behaviour: A systematic review to inform childhood obesity prevention | 33779040 | Not primary study on SR automation method or automated SR |
| Reece, 2021 | Delayed or failure to follow-up abnormal breast cancer screening mammograms in primary care: a systematic review | 33827476 | Not primary study on SR automation method or automated SR |
| Riaz, 2021 | Adjuvant Tyrosine Kinase Inhibitors in Renal Cell Carcinoma: A Concluded Living Systematic Review and Meta-Analysis | 34043431 | Not primary study on SR automation method or automated SR |
| Sabates, 2021 | CogTale: an online platform for the evaluation, synthesis, and dissemination of evidence from cognitive interventions studies | 34429154 | Not primary study on SR automation method or automated SR |
| Saiz, 2021 | Artificial Intelligence Clinical Evidence Engine for Automatic Identification, Prioritization, and Extraction of Relevant Clinical Oncology Research | 33439724 | Not primary study on SR automation method or automated SR |
| Schmidt, 2021 | Data extraction methods for systematic review (semi)automation: A living systematic review | 34408850 | Not primary study on SR automation method or automated SR |
| Scott, 2021 | Systematic review automation tools improve efficiency, but lack of knowledge impedes their adoption: a survey | 34242757 | Not primary study on SR automation method or automated SR |
| Seu, 2021 | Comparing Load-Sharing Miniplate and Load-Bearing Plate Fixation in Atrophic Edentulous Mandibular Fractures: A Systematic Review and Meta-Analysis | 34705386 | Not primary study on SR automation method or automated SR |
| Synnot, 2021 | A New Approach to Evidence Synthesis in Traumatic Brain Injury: A Living Systematic Review | 26414062 | Not primary study on SR automation method or automated SR |
| Taylor, 2021 | Extracting data from diagnostic test accuracy studies for meta-analysis | 33402330 | Full text not available |
| Turki, 2021 | Enhancing Knowledge Graph Extraction and Validation From Scholarly Publications Using Bibliographic Metadata | 34124535 | Not primary study on SR automation method or automated SR |
| Wang, 2021 | A systematic review of automatic text summarization for biomedical literature and EHRs | 34338801 | Not primary study on SR automation method or automated SR |
| Yang, 2021 | Tanshinone for polycystic ovary syndrome: A protocol of systematic review and meta-analysis | 33546053 | Not primary study on SR automation method or automated SR |
| Zorko, 2021 | Pediatric Chronic Critical Illness: Protocol for a Scoping Review | 34596576 | Not primary study on SR automation method or automated SR |
| Abba, 2022 | One Hundred Years of Hypertension Research: Topic Modeling Study | 35583933 | Not primary study on SR automation method or automated SR |
| Adam, 2022 | Semi-automated Tools for Systematic Searches | 34550582 | Full text not available |
| Adam, 2022 | A novel tool that allows interactive screening of PubMed citations showed promise for the semi-automation of identification of Biomedical Literature | 35738306 | Not primary study on SR automation method or automated SR |
| Alwakeel, 2022 | Functional and Technical Aspects of Self-management mHealth Apps: Systematic App Search and Literature Review | 35612887 | Not primary study on SR automation method or automated SR |
| Assef, 2022 | A review of clustering techniques for waste management | 35118205 | Not primary study on SR automation method or automated SR |
| Bevacqua, 2022 | The Potential of MicroRNAs as Non-Invasive Prostate Cancer Biomarkers: A Systematic Literature Review Based on a Machine Learning Approach | 36358836 | Not primary study on SR automation method or automated SR |
| Blaizot, 2022 | Using artificial intelligence methods for systematic review in health sciences: A systematic review | 35174972 | Not primary study on SR automation method or automated SR |
| Borissov, 2022 | Reducing systematic review burden using Deduklick: a novel, automated, reliable, and explainable deduplication algorithm to foster medical research | 35978441 | Not primary study on SR automation method or automated SR |
| Buchlak, 2022 | Natural Language Processing Applications in the Clinical Neurosciences: A Machine Learning Augmented Systematic Review | 34862552 | Full text not available |
| Cowie, 2022 | Web-Based Software Tools for Systematic Literature Review in Medicine: Systematic Search and Feature Analysis | 35499859 | Not primary study on SR automation method or automated SR |
| Das, 2022 | Structure-Activity Relationship Insight of Naturally Occurring Bioactive Molecules and Their Derivatives Against Non-Small Cell Lung Cancer: A Comprehensive Review | 35579166 | Not primary study on SR automation method or automated SR |
| El Idrissi, 2022 | Exploration of the core protein network under endometriosis symptomatology using a computational approach | 36120440 | Not primary study on SR automation method or automated SR |
| Fathabadipour, 2022 | The neural effects of oxytocin administration in autism spectrum disorders studied by fMRI: A systematic review | 35933858 | Not primary study on SR automation method or automated SR |
| Feng, 2022 | Automated medical literature screening using artificial intelligence: a systematic review and meta-analysis | 35641139 | Not primary study on SR automation method or automated SR |
| Fernandez-Perez, 2022 | Use of autobiographical stimuli as a mood manipulation procedure: Systematic mapping review | 35759458 | Not primary study on SR automation method or automated SR |
| Gao, 2022 | A scoping review of publicly available language tasks in clinical natural language processing | 35923088 | Not primary study on SR automation method or automated SR |
| Guan, 2022 | Discovering trends and hotspots of biosafety and biosecurity research via machine learning | 35596953 | Not primary study on SR automation method or automated SR |
| Guimaraes, 2022 | Deduplicating records in systematic reviews: there are free, accurate automated ways to do so | 36241035 | Not primary study on SR automation method or automated SR |
| Gyamfi, 2022 | Intrusion Detection in Internet of Things Systems: A Review on Design Approaches Leveraging Multi-Access Edge Computing, Machine Learning, and Datasets | 35632153 | Not primary study on SR automation method or automated SR |
| Kebede, 2022 | In-depth evaluation of machine learning methods for semi-automating article screening in a systematic review of mechanistic literature | 35798691 | Not primary study on SR automation method or automated SR |
| Knipe, 2022 | Suicide and self-harm in low- and middle- income countries during the COVID-19 pandemic: A systematic review | 36962383 | Not primary study on SR automation method or automated SR |
| Kubiak, 2022 | Possible Applications of Edge Computing in the Manufacturing Industry-Systematic Literature Review | 35408059 | Not primary study on SR automation method or automated SR |
| Kumar, 2022 | What changed in the cyber-security after COVID-19? | 35813991 | Not primary study on SR automation method or automated SR |
| Lam, 2022 | Examining patterns of traditional Chinese medicine use in pediatric oncology: A systematic review, meta-analysis and data-mining study | 35750623 | Not primary study on SR automation method or automated SR |
| Lopez-Ubeda, 2022 | Natural Language Processing in Pathology: Current Trends and Future Insights | 35985480 | Not primary study on SR automation method or automated SR |
| Lu, 2022 | Assessment of Adherence to Reporting Guidelines by Commonly Used Clinical Prediction Models From a Single Vendor: A Systematic Review | 35984654 | Not primary study on SR automation method or automated SR |
| Marshall, 2022 | In a pilot study, automated real-time systematic review updates were feasible, accurate, and work-saving | 36150548 | Not primary study on SR automation method or automated SR |
| May, 2022 | Translational framework for implementation evaluation and research: Protocol for a qualitative systematic review of studies informed by Normalization Process Theory (NPT) [version 1 | 35935672 | Not primary study on SR automation method or automated SR |
| McParland, 2022 | A mixed-methods systematic review of nurse-led interventions for people with multimorbidity | 36065516 | Not primary study on SR automation method or automated SR |
| Minshall, 2022 | Towards the Adoption of Novel Visualizations in Public Health | 35773826 | Not primary study on SR automation method or automated SR |
| Morales, 2022 | Mental Pain Surrounding Suicidal Behaviour: A Review of What Has Been Described and Clinical Recommendations for Help | 35153847 | Not primary study on SR automation method or automated SR |
| Naskath, 2022 | A Study on Different Deep Learning Algorithms Used in Deep Neural Nets: MLP SOM and DBN | 36276226 | Not primary study on SR automation method or automated SR |
| Nieblas, 2022 | Impact and future of telemedicine amidst the COVID-19 pandemic: a systematic review of the state-of-the-art in Latin America | 35894315 | Not primary study on SR automation method or automated SR |
| Puertas, 2022 | Environmental policies for the treatment of waste generated by COVID-19: Text mining review | 35282720 | Full text not available |
| Rezaeenour, 2022 | Systematic review of content analysis algorithms based on deep neural networks | 36313481 | Not primary study on SR automation method or automated SR |
| Salholz-Hillel, 2022 | Results publications are inadequately linked to trial registrations: An automated pipeline and evaluation of German university medical centers | 35362331 | Not primary study on SR automation method or automated SR |
| Shah, 2022 | Twitter Research Synthesis for Health Promotion: A Bibliometric Analysis | 35223603 | Not primary study on SR automation method or automated SR |
| Shahini, 2022 | Effects of levels of automation and non-driving related tasks on driver performance and workload: A review of literature and meta-analysis | 35724471 | Not primary study on SR automation method or automated SR |
| Shemilt, 2022 | Machine learning reduced workload for the Cochrane COVID-19 Study Register: development and evaluation of the Cochrane COVID-19 Study Classifier | 35065679 | Not primary study on SR automation method or automated SR |
| Smalheiser, 2022 | A web-based tool for automatically linking clinical trials to their publications | 35020887 | Not primary study on SR automation method or automated SR |
| Tercero-Hidalgo, 2022 | Artificial intelligence in COVID-19 evidence syntheses was underutilized, but impactful: a methodological study | 35513213 | Not primary study on SR automation method or automated SR |
| Tozzi, 2022 | Gaps and Opportunities of Artificial Intelligence Applications for Pediatric Oncology in European Research: A Systematic Review of Reviews and a Bibliometric Analysis | 35712463 | Not primary study on SR automation method or automated SR |
| Urru, 2022 | A topic trend analysis on COVID-19 literature | 36325437 | Not primary study on SR automation method or automated SR |
| Wang, 2022 | Predicting the targets of IRF8 and NFATc1 during osteoclast differentiation using the machine learning method framework cTAP | 34991467 | Not primary study on SR automation method or automated SR |
| Yang, 2022 | [Artificial intelligence-based literature data warehouse for vaccine safety] | 35345302 | Not English |
| Yiyi, 2022 | Spleen Stiffness on Magnetic Resonance Elastography for the Detection of Portal Hypertension: A Systematic Review and Meta-Analysis | 36743372 | Not primary study on SR automation method or automated SR |
| You, 2022 | The Microultrasound-Guided Prostate Biopsy in Detection of Prostate Cancer: A Systematic Review and Meta-Analysis | 34569293 | Full text not available |
| Yurasakpong, 2022 | The decreasing prevalence of the thyroid ima artery: A systematic review and machine learning assisted meta-analysis | 34265384 | Not primary study on SR automation method or automated SR |
| Zhang, 2022 | Automation of literature screening using machine learning in medical evidence synthesis: a diagnostic test accuracy systematic review protocol | 35031074 | Not primary study on SR automation method or automated SR |
| Zhang, 2022 | Bone regeneration materials and their application over 20 years: A bibliometric study and systematic review | 36277397 | Not primary study on SR automation method or automated SR |
| Zhang, 2022 | Research hotspots and frontiers in agricultural multispectral technology: Bibliometrics and scientometrics analysis of the Web of Science | 36035687 | Not primary study on SR automation method or automated SR |
| Zhang, 2022 | Information Extraction from the Text Data on Traditional Chinese Medicine: A Review on Tasks, Challenges, and Methods from 2010 to 2021 | 35600940 | Not primary study on SR automation method or automated SR |
