# Supplementary material for: Automation of systematic reviews of biomedical literature: a scoping review of studies indexed in PubMed
Source: Syst Rev. 2024 Jul 8;13:174. doi: 10.1186/s13643-024-02592-3 (PMC11229257; doi:10.1186/s13643-024-02592-3)
Supplement: Supplementary file 4 — Additional file 4. Details of included studies. [file 13643_2024_2592_MOESM4_ESM.docx]

**Additional file 4. Details of included studies**

A list of abbreviations is provided at the end of the table.

| Cohen, 2006 (32) | PMID | 16357352 |
| --- | --- | --- |
|  | Title | Reducing workload in systematic review preparation using automated citation classification |
|  | Automation stage, aim | Record screening (automated classification); test potential workload saving with automated screening |
|  | Input | Title, abstract, MeSH terms |
|  | Text representation | BOW (binary) |
|  | Model | Voting perceptron classifier |
|  | Corpus | Oregon EPC DERP (TREC 2004 Genomics Track) [https://dmice.ohsu.edu/cohenaa/systematic-drug-class-review-data.html (http://medir.ohsu.edu/∼cohenaa)] |
|  | Automation tool | - |
|  | Comment | - |
|  | Key result | WSS@95 ranged from 0.31%-70.5% among 15 SRs |
|  | Google Scholar citations | 320 |
| Cohen, 2008 (119) | PMID | 18998798 |
|  | Title | Optimizing feature representation for automated systematic review work prioritization |
|  | Automation stage, aim | Record screening (priority ranking) |
|  | Input | Title, abstract |
|  | Text representation | BOW, semantic annotation (UMLS concepts to MeSH) |
|  | Model | SVM |
|  | Corpus | - |
|  | Automation tool | MetaMap, Apache Lucene |
|  | Comment | Compare feature sets and cross-topic vs topic specific learning |
|  | Key result | topic specific learning with BOW of uni- and bigrams and MeSH terms had best performance, with mean AUC: 0.866 across 15 SRs |
|  | Google Scholar citations | 89 |
| Yang, 2008 (107) | PMID | 18999194 |
|  | Title | SYRIAC: The systematic review information automated collection system a data warehouse for facilitating automated biomedical text classification |
|  | Automation stage, aim | Record screening (review update); a standardised database for training data of SR updates |
|  | Input | EndNote records |
|  | Text representation | Annotated records with SR topic, PMID and reviewer decisions |
|  | Model | - |
|  | Corpus | EPC DERP, SYRIAC |
|  | Automation tool | SYRIAC |
|  | Comment | - |
|  | Key result | PMID recovery rate: 82.3% Proportion of correct PMIDs: 99.6% |
|  | Google Scholar citations | 23 |
| Yu, 2008 (52) | PMID | 18430222 |
|  | Title | GAPscreener: an automatic tool for screening human genetic association literature in PubMed using the support vector machine technique |
|  | Automation stage, aim | Record screening (automated classification); screening of human gene association studies |
|  | Input | Title, abstract |
|  | Text representation | BOW, keywords relevant to gene association studies |
|  | Model | SVM |
|  | Corpus | National Office of Public Health Genomics, HuGE Navigator |
|  | Automation tool | GAP Screener [http://www.hugenavigator.net/HuGENavigator/HNDescription/opensource_GAP.htm] |
|  | Comment | Two keyword weighting schemes |
|  | Key result | 2-yway keyword weighting, performance over 5 weeks vs manually annotated gold standard week 1: P:0.345@R:0.946 week 2 P:0.311@R:0.992 week 3 P:0.291@R:0.967 week 4 P:0.323@R:0.977 week 5 P:0.336@R:0.993 Mean WLS over 4 weeks: 87.5%   Screening preterm birth studies: with 90.2% WLS, from 55 relevant studies GAP Screener identified correctly 96.4% (missed 2), human reviewers identified 92.7% (missed 6) |
|  | Google Scholar citations | 63 |
| Cohen, 2009 (120) | PMID | 19567792 |
|  | Title | Cross-topic learning for work prioritization in systematic review creation and update |
|  | Automation stage, aim | Record screening (priority ranking); combine cross-topic learning (records of other SRs) and topic learning (records of the same SR) in screening |
|  | Input | Title, abstract, MeSH terms |
|  | Text representation | BOW |
|  | Model | SVM |
|  | Corpus | Oregon EPC DERP, SYRIAC [0] |
|  | Automation tool | - |
|  | Comment | - |
|  | Key result | A hybrid system of cross-topic and topic specific training improved mean AUC by 20% across 24 SRs.  Mean AUC hybrid training: 0.80, mean AUC topic-specific training: 0.67 |
|  | Google Scholar citations | 95 |
| Bekhuis, 2010 (53) | PMID | 20841667 |
|  | Title | Towards automating the initial screening phase of a systematic review |
|  | Automation stage, aim | Record screening (automated classification); optimise screener performance (feature set, classifiers) for a large SR including observational studies |
|  | Input | Title, abstract, metadata |
|  | Text representation | TF-IDF |
|  | Model | Evolutionary SVM |
|  | Corpus | - |
|  | Automation tool | RapidMiner [http://wvtool.sourceforge.net] |
|  | Comment | Various training set sizes |
|  | Key result | P:0.263 - 0.370@R:0.769 |
|  | Google Scholar citations | 50 |
| Cohen, 2010 (121) | PMID | 21346953 |
|  | Title | A Prospective Evaluation of an Automated Classification System to Support Evidence-based Medicine and Systematic Review |
|  | Automation stage, aim | Record screening (priority ranking); prospective evaluation of a screener tool simulating real-world use |
|  | Input | Title, abstract, MeSH terms |
|  | Text representation | BOW |
|  | Model | SVM |
|  | Corpus | Oregon EPC DERP, SYRIAC |
|  | Automation tool | - |
|  | Comment | - |
|  | Key result | AUC: 0.752-0.994 among 15 SRs |
|  | Google Scholar citations | 38 |
| Kiritchenko, 2010 (138) | PMID | 20920176 |
|  | Title | ExaCT: automatic extraction of clinical trial characteristics from journal publications |
|  | Automation stage, aim | Data extraction: introduce and evaluate exact, a tool to extract 21 study characteristics from full text RCT reports |
|  | Input | Full-text |
|  | Text representation | Bag of terms by sentence, sentence classification by information content, rule-based fragment extraction using regular expressions |
|  | Model | SVM |
|  | Corpus | - |
|  | Automation tool | ExaCT [http://exactdemo.iit.nrc.ca] |
|  | Comment | - |
|  | Key result | Identification of relevant sentences: P:0.80@R:0.88 extraction of data pieces: P:0.93@R:0.91, proportion of extraction with partially correct solutions: 94%. |
|  | Google Scholar citations | 160 |
| Matwin, 2010 (54) | PMID | 20595313 |
|  | Title | A new algorithm for reducing the workload of experts in performing systematic reviews |
|  | Automation stage, aim | Record screening (automated classification); improve screening performance vs the baseline machine learning method |
|  | Input | Title, abstract, MeSH terms |
|  | Text representation | BOW, feature weight engineering |
|  | Model | Factorised complement naive Bayes (FCNB) |
|  | Corpus | Oregon EPC DERP |
|  | Automation tool | - |
|  | Comment | Various hyperparameters for the classifier and text feature weights |
|  | Key result | On 15 SRs WSS@95: 33.5% (range: 8.5%-62.2%) |
|  | Google Scholar citations | 114 |
| Wallace, 2010 (83) | PMID | 20102628 |
|  | Title | Semi-automated screening of biomedical citations for systematic reviews |
|  | Automation stage, aim | Record screening (active learning); active learning by addressing class imbalance |
|  | Input | Title, abstract, keywords |
|  | Text representation | TF-IDF, UMLS concepts (4 feature spaces per document) |
|  | Model | SVM ensemble (1 SVM per feature space) |
|  | Corpus | EPPI Centre datasets Proton Beam COPD Micronutrients |
|  | Automation tool | Curious snake [Python], MetaMap [http://www.nlm.nih.gov/research/umls/mmtx.html https://www.ncbi.nlm.nih.gov/pmc/articles/PMC2824679/bin/1471-2105-11-55-S1.ZIP] |
|  | Comment | Aggressive undersampling, Patient Active Learning |
|  | Key result | in 3 SRs WLS: ∼40-50%@R:1.00 |
|  | Google Scholar citations | 323 |
| Frunza, 2011 (55) | PMID | 21084178 |
|  | Title | Exploiting the systematic review protocol for classification of medical abstracts |
|  | Automation stage, aim | Record screening (automated classification); screening performance of a global classifier (single i/e decision) or multiple-question classifier following the SR protocol questions |
|  | Input | Title, abstract |
|  | Text representation | BOW, semantic annotation (UMLS concepts) |
|  | Model | Complement naive Bayes |
|  | Corpus | McMaster University Evidence-Based Practice Center (EPC) |
|  | Automation tool | MetaMap, TrialStat SRS |
|  | Comment | Compare various text representations and sampling ratios |
|  | Key result | The proposed workflow: human (single reviewer) and machine screening, BOW + UMLS, two protocol questions. P:0.17@R:0.927 |
|  | Google Scholar citations | 55 |
| Oertelt-Prigione, 2011 (33) | PMID | 21252506 |
|  | Title | Stroke and myocardial infarction: a comparative systematic evaluation of gender-specific analysis, funding and authorship patterns in cardiovascular research |
|  | Automation stage, aim | Search: apply text-mining to aid PubMed search |
|  | Input | - |
|  | Text representation | - |
|  | Model | - |
|  | Corpus | - |
|  | Automation tool | Apache Lucene |
|  | Comment | - |
|  | Key result | na |
|  | Google Scholar citations | 17 |
| Dalal, 2012 (117) | PMID | 23057094 |
|  | Title | A Pilot Study Using Machine Learning and Domain Knowledge To Facilitate Comparative Effectiveness Review Updating |
|  | Automation stage, aim | Record screening (review update); automate screening for SR updates |
|  | Input | Title, MeSH terms |
|  | Text representation | Rule-based extraction of 121 predefined binary keyword variables |
|  | Model | Gradient Boosting Machine (GBM), GLMNet |
|  | Corpus | - |
|  | Automation tool | [R, Python] |
|  | Comment | - |
|  | Key result | SR on osteoporosis medications: WLS:55.4%@R:0.99; SR on antipsychotics: WLS: 63.4%@R:1.00 |
|  | Google Scholar citations | 32 |
| Hempel, 2012 (82) | PMID | 23101052 |
|  | Title | Machine Learning Methods in Systematic Reviews: Identifying Quality Improvement Intervention Evaluations |
|  | Automation stage, aim | Record screening (automated classification); compare automated vs manual screening in an SR on quality improvement |
|  | Input | Title, abstract, MeSH terms |
|  | Text representation | BOW |
|  | Model | LASSO |
|  | Corpus | - |
|  | Automation tool | - |
|  | Comment | - |
|  | Key result | P:0.321@R:0.901, WLS: 36.1% |
|  | Google Scholar citations | 18 |
| Wallace, 2012 (108) | PMID | 22481134 |
|  | Title | Toward modernizing the systematic review pipeline in genetics: efficient updating via data mining |
|  | Automation stage, aim | Record screening (review update); evaluate automated classification in the update of genetic association studies |
|  | Input | Title, abstract, MeSH terms |
|  | Text representation | BOW |
|  | Model | SVM ensemble with bagging |
|  | Corpus | PDGene AlzGene SzGene Tufts CEA Registry [http://www.pdgene.org http://www.alzqene.org http://www.szgene.org httos://research.tufts-nemc. org/cear4/] |
|  | Automation tool | [Python] |
|  | Comment | Random undersampling, error analysis |
|  | Key result | Performance for 1-year update PDGene P:0.172@R:1.00 AlzGene P:0.117@R:1.00 SzGene P:0.25@R:1.00 CA Registry P:0.234@R:0.987 |
|  | Google Scholar citations | 79 |
| Huang, 2013 (56) | PMID | 23899909 |
|  | Title | PICO element detection in medical text without metadata: are first sentences enough? |
|  | Automation stage, aim | Record screening (automated classification); to detect PICO elements in the first sentences of sections in structured RCT abstracts |
|  | Input | Structured abstracts |
|  | Text representation | BOW |
|  | Model | Naive Bayes |
|  | Corpus | Medline [ http://kimiko.biome.tk/2013_PICO/] |
|  | Automation tool | - |
|  | Comment | Classifiers trained on various feature word counts |
|  | Key result | The first sentence of abstract sections does not contain enough information to accurately detect PICO elements |
|  | Google Scholar citations | 53 |
| Bekhuis, 2014 (57) | PMID | 24475099 |
|  | Title | Feature engineering and a proposed decision-support system for systematic reviewers of medical evidence |
|  | Automation stage, aim | Record screening (automated classification); optimise screener performance by feature set selection and human error analysis |
|  | Input | Title, abstract, metadata |
|  | Text representation | TF-IDF |
|  | Model | Complement naive Bayes |
|  | Corpus | - |
|  | Automation tool | RapidMiner, EDDA, IndexFinder[Java] |
|  | Comment | 5 feature sets compared: alphabetic, alphanumeric, Mesh/Emtree indexing terms, PICO concepts represented via UMLS, topics |
|  | Key result | P: 0.138-0.724, R:0.597 - 0.968 |
|  | Google Scholar citations | 44 |
| Miwa, 2014 (84) | PMID | 24954015 |
|  | Title | Reducing systematic review workload through certainty-based screening |
|  | Automation stage, aim | Record screening (active learning); compare certainty vs uncertainty-based active learning strategies in screening |
|  | Input | Title, abstract, MeSH terms |
|  | Text representation | BOW, feature weight engineering, topic modelling (LDA) |
|  | Model | SVM |
|  | Corpus | EPPI Center datasets (Clinical: Proton Beam, Micronutrients, COPD; Social:  Cooking Skills, Sanitation, Tobacco, Packaging, Youth, Development) |
|  | Automation tool | - |
|  | Comment | Various scenarios of active learning (certainty/uncertainty/random), feature weighting (covariate shift), sampling weights (for data imbalance), ensemble classifiers |
|  | Key result | Best performance of certainty-based active learning with weighting to correct data imbalance. Results are provided graphically. |
|  | Google Scholar citations | 173 |
| Mytton, 2014 (136) | PMID | 23640123 |
|  | Title | Facilitators and barriers to engagement in parenting programs: a qualitative systematic review |
|  | Automation stage, aim | Record screening (); use automatic term recognition to screen relevant records in a SR of qualitative studies |
|  | Input | Title, abstract |
|  | Text representation | - |
|  | Model | - |
|  | Corpus | - |
|  | Automation tool | EPPI-Reviewer 4 |
|  | Comment | - |
|  | Key result | na |
|  | Google Scholar citations | 220 |
| O'Mara-Eves, 2014 (34) | PMID | 26054025 |
|  | Title | Techniques for identifying cross-disciplinary and 'hard-to-detect' evidence for systematic review |
|  | Automation stage, aim | Search: text mining in known relevant systematic reviews for search term keywords to facilitate the retrieval of hard to detect evidence |
|  | Input | Full-text of relevant reviews |
|  | Text representation | - |
|  | Model | - |
|  | Corpus | - |
|  | Automation tool | TerMine [http://www.nactem.ac.uk/software/termine/] |
|  | Comment | - |
|  | Key result | In addition to 226 relevant papers identified through traditional search, 95 studies were identified using the extended search (+40.3%) |
|  | Google Scholar citations | 29 |
| Shao, 2014 (58) | PMID | 25461812 |
|  | Title | Aggregator: a machine learning approach to identifying MEDLINE articles that derive from the same underlying clinical trial |
|  | Automation stage, aim | Record screening (automated classification); tag PubMed articles from the same clinical trial |
|  | Input | Medline records with metadata |
|  | Text representation | 12 features engineered (rank in related articles, shared author names, affiliation similarity, shared e-mail, publication type, support type, e-mail, country, grant number, substance names, all-capitalised words in title, all-capitalised words in abstract or CN field, NCT numbers) |
|  | Model | Logistic regression |
|  | Corpus | - |
|  | Automation tool | Aggregator |
|  | Comment | Different PubMed queries tested |
|  | Key result | Evaluation in 42 PubMed Queries.  Split: proportion of reports from the same trial, not identified as such Purity: proportion of reports wrongly associated to the same trial Clinical condition, all studies: Split: 0.105 Purity: 0.74 Clinical condition, studies with NCT: Split:0.107 Purity: 0.72 Clinical condition + intervention, all studies: Split:0.0 Purity: 0.86 Clinical condition + intervention, studies with NCT: Split: 0.0078 Purity:0.79 |
|  | Google Scholar citations | 15 |
| Shemilt, 2014 (129) | PMID | 26054024 |
|  | Title | Pinpointing needles in giant haystacks: use of text mining to reduce impractical screening workload in extremely large scoping reviews |
|  | Automation stage, aim | Record screening (active learning, review update); screening via text-mining in extremely large scoping reviews |
|  | Input | Title, abstract |
|  | Text representation | Automated term recognition, automated classification, reviewer terms |
|  | Model | SVM |
|  | Corpus | - |
|  | Automation tool | EPPI Reviewer, TerMine [http://www.nactem.ac.uk/software/termine/] |
|  | Comment | - |
|  | Key result | In two large reviews Choice architecture (n=430 000): WLS: 90.1%, ratio of automated vs baseline inclusion rate: 10.1 Economic environment (n=378 000): WLS: 88.0%, ratio of automated vs baseline inclusion rate: 8.3 |
|  | Google Scholar citations | 156 |
| Thompson, 2014 (35) | PMID | 26052649 |
|  | Title | A systematic method for search term selection in systematic reviews |
|  | Automation stage, aim | Search; text mining for search terms for a SR |
|  | Input | Title, abstract, full-text |
|  | Text representation | - |
|  | Model | - |
|  | Corpus | SciVerse Hub |
|  | Automation tool | Leximancer |
|  | Comment | - |
|  | Key result | The process of generating search keywords for a published review is demonstrated |
|  | Google Scholar citations | 22 |
| Blake, 2015 (142) | PMID | 26003938 |
|  | Title | Automatic endpoint detection to support the systematic review process |
|  | Automation stage, aim | Data extraction; identify comparative claim sentences and their entities |
|  | Input | Full-text |
|  | Text representation | Semantic analysis: comparative sentences and compared entities using UMLS and Stanford Parser |
|  | Model | SVM, GLM |
|  | Corpus | TREC collection |
|  | Automation tool | Oracle Data Miner |
|  | Comment | - |
|  | Key result | In a SR application accuracy was 95% for object, 83% for endpoint and 79% for agent recognition. |
|  | Google Scholar citations | 22 |
| Bui, 2015 (36) | PMID | 26363352 |
|  | Title | Automatically finding relevant citations for clinical guideline development |
|  | Automation stage, aim | Search: retrieve citations that support cardiovascular guideline recommendations |
|  | Input | Title, abstract |
|  | Text representation | Semantic annotation (UMLS concepts to MeSH terms) |
|  | Model | Rule-based scoring using MeSH major terms, study design and Scimago Journal Rank |
|  | Corpus | American College of Cardiology (ACC) Guidelines |
|  | Automation tool | MetaMap [0] |
|  | Comment | - |
|  | Key result | P:0.004@R:80.2 in retrieving records that support a guideline recommendation |
|  | Google Scholar citations | 19 |
| Cohen, 2015 (59) | PMID | 25656516 |
|  | Title | Automated confidence ranked classification of randomized controlled trial articles: an aid to evidence-based medicine |
|  | Automation stage, aim | Record screening (automated classification); confidence score-based prediction if an article is RCT |
|  | Input | Bibliographic record |
|  | Text representation | BOW and MeSH terms |
|  | Model | SVM |
|  | Corpus | PubMed |
|  | Automation tool | RCT Tagger [ http://arrowsmith.psych.uic.edu/cgi-bin/arrowsmith_uic/RCT_Tagger.cgi] |
|  | Comment | Compare various feature sets and models |
|  | Key result | AUC: 0.973, 3% RCTs are missed by Medline indexing |
|  | Google Scholar citations | 56 |
| Dunn, 2015 (85) | PMID | 26262175 |
|  | Title | Identifying Clinical Study Types from PubMed Metadata: The Active (Machine) Learning Approach |
|  | Automation stage, aim | Record screening (active learning); classify article types via active learning |
|  | Input | Title, abstract, MeSH terms, publication type |
|  | Text representation | BOW |
|  | Model | SVM |
|  | Corpus | - |
|  | Automation tool | - |
|  | Comment | - |
|  | Key result | Clinical trials: P:0.96@R:0.93 RCTs: P:0.99@R:0.98 Reviews and opinion papers: P:0.97@R:0.97 |
|  | Google Scholar citations | 2 |
| Ji, 2015 (130) | PMID | 26323593 |
|  | Title | Using MEDLINE Elemental Similarity to Assist in the Article Screening Process for Systematic Reviews |
|  | Automation stage, aim | Record screening (similarity for Medline elements); screening using a network representation of articles based on Medline elements |
|  | Input | Title, abstract, publication type, MeSH terms, author |
|  | Text representation | Alphabetic features |
|  | Model | Cosine similarity |
|  | Corpus | Oregon EPC DERP |
|  | Automation tool | - |
|  | Comment | - |
|  | Key result | In 15 SRS including title, abstract, publication type: WSS@95: 37.06% (range: 8.98%-74.29%) |
|  | Google Scholar citations | 18 |
| Llewellyn, 2015 (23) | PMID | 26716874 |
|  | Title | The Use of Bayesian Networks to Assess the Quality of Evidence from Research Synthesis: 2. Inter-Rater Reliability and Comparison with Standard GRADE Assessment |
|  | Automation stage, aim | Assessment of evidence quality; evaluate the performance of SAQAT, a semi-automated tool to assess evidence quality. |
|  | Input | Full-text |
|  | Text representation | - |
|  | Model | Bayesian Network |
|  | Corpus | Database of Systematic Reviews of Effects (DARE) |
|  | Automation tool | SAQAT |
|  | Comment | - |
|  | Key result | Agreement on GRADE rating SAQAT vs two independent reviewers: Cohen's kappa = 0.79 (Substantial) SAQAT vs GRADE rating: Cohen's kappa = 0.35 (Fair) |
|  | Google Scholar citations | 9 |
| Mo, 2015 (60) | PMID | 26612232 |
|  | Title | Supporting systematic reviews using LDA-based document representations |
|  | Automation stage, aim | Record screening (automated classification); topic distribution-based text representation for automated classification in screening |
|  | Input | Title, abstract |
|  | Text representation | TF-IDF, topic modelling (LDA), automatic term recognition |
|  | Model | SVM |
|  | Corpus | EPPI-centre datasets (Proton Beam,  COPD, Tobacco Packaging, Cooking Skill, Youth Development) |
|  | Automation tool | TerMine [0] |
|  | Comment | While BOW models had best classification performance except recall. Topic feature representations outperformed BOW in recall.  P:0.032-0.418@R:0.606-0.986 for topic feature representations. |
|  | Key result | - |
|  | Google Scholar citations | 76 |
| Mwachui, 2015 (37) | PMID | 26379035 |
|  | Title | Environmental and Behavioural Determinants of Leptospirosis Transmission: A Systematic Review |
|  | Automation stage, aim | Search: update a review on human leptospirosis using an optimised search filter based on text-mining |
|  | Input | - |
|  | Text representation | - |
|  | Model | Markov-chain model |
|  | Corpus | Leptospirosis Burden Epidemiology Reference Group (LERG) of the WHO |
|  | Automation tool | [R] [https://www.ncbi.nlm.nih.gov/pmc/articles/PMC4574979/bin/pntd.0003843.s003.pdf] |
|  | Comment | - |
|  | Key result | 13 papers / 229 abstracts (5.6%) included vs 53/12025 (0.44%) in the original review |
|  | Google Scholar citations | 304 |
| Stewart, 2015 (24) | PMID | 25837450 |
|  | Title | The use of Bayesian networks to assess the quality of evidence from research synthesis: 1 |
|  | Automation stage, aim | Assessment of evidence quality; automate grade assessment of evidence |
|  | Input | - |
|  | Text representation | - |
|  | Model | Bayesian Network, expert-driven probability weights |
|  | Corpus | Cochrane |
|  | Automation tool | SAQAT |
|  | Comment | - |
|  | Key result | Preliminary assessment: agreement with Cochrane reviews, Cohen's kappa: 0.69 |
|  | Google Scholar citations | 15 |
| Almeida, 2016 (61) | PMID | 28113721 |
|  | Title | Data Sampling and Supervised Learning for HIV Literature Screening |
|  | Automation stage, aim | Record screening (automated classification); compare various classifier, feature and sampling ratio settings for better screening performance. |
|  | Input | Title, abstract |
|  | Text representation | BOW and MeSH terms |
|  | Model | Logistic Model Trees Classifier |
|  | Corpus | SHARE [http://www.hivevidence.ca ] |
|  | Automation tool | - |
|  | Comment | Various sampling ratio settings |
|  | Key result | P:0.467@R:0.900 |
|  | Google Scholar citations | 22 |
| Bui, 2016 (51) | PMID | 27989816 |
|  | Title | Extractive text summarization system to aid data extraction from full text in systematic review development |
|  | Automation stage, aim | Data extraction: produce full-text summaries to aid data extraction |
|  | Input | Full-text |
|  | Text representation | Map full-text to IMRAD structure, semantic annotation (UMLS concepts) and data extraction template synonyms, prioritise sentences |
|  | Model | SVM |
|  | Corpus | Cochrane Library |
|  | Automation tool | Apache PDFBox, MetaMap |
|  | Comment | Various feature representation sets |
|  | Key result | P:0.59@R:0.912 in identifying relevant sentences for the data-extraction template vs human-written title and abstract |
|  | Google Scholar citations | 56 |
| Hashimoto, 2016 (86) | PMID | 27293211 |
|  | Title | Topic detection using paragraph vectors to support active learning in systematic reviews |
|  | Automation stage, aim | Record screening (active learning); active screening via clustering of records using a novel paragraph vector based topic model vs conventional (LDA) topic model |
|  | Input | Title, abstract |
|  | Text representation | Paragraph to vector, topic modelling (LDA), topic distribution |
|  | Model | SVM |
|  | Corpus | EPPI Center datasets (Proton Beam, COPD, Cooking Skills, Tobacco Packaging, Youth Development) |
|  | Automation tool | - |
|  | Comment | Various text representation and topic modelling scenarios compared |
|  | Key result | Proposed model / baseline on COPD dataset:  Burden:17%@R:0.91 / Burden:17%@R:0.87 Burden:20%@R:0.96 / Burden:20%@R:0.89 WSS@95 improvements vs baseline: 1-15% |
|  | Google Scholar citations | 116 |
| Howard, 2016 (122) | PMID | 27216467 |
|  | Title | SWIFT-Review: a text-mining workbench for systematic review |
|  | Automation stage, aim | Record screening (priority ranking); introduce and test swift-review performance in screening |
|  | Input | Title, abstract, MeSH terms |
|  | Text representation | BOW, TF-IDF, concept features |
|  | Model | Log-linear model fitted with limited memory Broyden-Fletcher-Goldfarb-Shanno (LBGFS) algorithm (i.e., maximum entropy model) |
|  | Corpus | National Institutes of Environmental Health Sciences/National Toxicology Program (NIEHS) CAMARADES (Collaborative Approach to Meta Analysis and Review of Animal Experimental Studies) Oregon EPC DERP [https://www.ncbi.nlm.nih.gov/pmc/articles/PMC4877757/bin/13643_2016_263_MOESM1_ESM.xlsx https://www.ncbi.nlm.nih.gov/pmc/articles/PMC4877757/bin/13643_2016_263_MOESM2_ESM.xlsx na] |
|  | Automation tool | SWIFT-Review  [http://swift.sciome.com/] |
|  | Comment | - |
|  | Key result | In 20 SRs mean WSS@95: 0.540, range (0.137-0.870) |
|  | Google Scholar citations | 140 |
| Li, 2016 (123) | PMID | 29071308 |
|  | Title | A Text-Mining Framework for Supporting Systematic Reviews |
|  | Automation stage, aim | Record screening (priority ranking); relevance ranking for screening based on keyword relevance, index term relevance and topic relevance |
|  | Input | Keyword list, search strategy, abstract, MeSH terms |
|  | Text representation | Lucene score (TF-IDF-based relevance) for keywords, MeSH terms and topic modelling (LDA) |
|  | Model | - |
|  | Corpus | - |
|  | Automation tool | - |
|  | Comment | - |
|  | Key result | 3 case studies:  Mass Media Intervention (300/3303 records screened) P:0.023@R:1.00 WLS: 91.8% Rectal cancer study (600/4075 records screened) P:0.017@R:1.00, WLS: 85.7% Flu vaccine study: (400/811 records screened) P:0.12@R:0.98, WLS:49.3% |
|  | Google Scholar citations | 23 |
| Marshall, 2016 (145) | PMID | 26104742 |
|  | Title | RobotReviewer: evaluation of a system for automatically assessing bias in clinical trials |
|  | Automation stage, aim | Risk of bias assessment; introduce and evaluate RobotReviewer, a system to automate risk of bias assessment following the Cochrane Risk of Bias tool |
|  | Input | Full-text |
|  | Text representation | Interacted document and sentence-level features |
|  | Model | SVM, distant supervision |
|  | Corpus | Cochrane Database of Systematic Reviews (CDSR) |
|  | Automation tool | RobotReviewer [https://www.github.com/ijmarshall ] |
|  | Comment | Various feature sets |
|  | Key result | Agreement in overall risk of bias assessment for RCTs included in at least 2 Cochrane reviews RobotReviewer w humans: 71% Two Cochrane reviews: 78% |
|  | Google Scholar citations | 217 |
| Millard, 2016 (146) | PMID | 26659355 |
|  | Title | Machine learning to assist risk-of-bias assessments in systematic reviews |
|  | Automation stage, aim | Risk of bias assessment; 0 |
|  | Input | Full-text |
|  | Text representation | Alphabetic features, sentence tagging, document tagging |
|  | Model | Logistic regression |
|  | Corpus | Cochrane Database of Systematic Reviews |
|  | Automation tool | Systematic Review Assistant (prototype) [http://www.datamining.org.uk/sysreview.html] |
|  | Comment | - |
|  | Key result | Find relevant sentences: AUC>0.98, rank articles by RoB: AUC>0.72, risk of bias assessment with greater certainty than human reviewers: 33% |
|  | Google Scholar citations | 65 |
| Ouzzani, 2016 (87) | PMID | 27919275 |
|  | Title | Rayyan-a web and mobile app for systematic reviews |
|  | Automation stage, aim | Record screening (active learning); introduce and test Rayyan’s usability and screening performance |
|  | Input | Title, abstract |
|  | Text representation | BOW |
|  | Model | SVM |
|  | Corpus | Oregon EPC DERP |
|  | Automation tool | Rayyan [http://rayyan.qcri.org] |
|  | Comment | - |
|  | Key result | On 15 SRs mean WSS@95: 49% Users reported on average ∼40% time saving |
|  | Google Scholar citations | 9015 |
| Sampson, 2016 (38) | PMID | 26976054 |
|  | Title | Complementary approaches to searching MEDLINE may be sufficient for updating systematic reviews |
|  | Automation stage, aim | Search: test the performance of three Medline search strategies to update SRs: PubMed clinical query, PubMed related articles, search via SVM |
|  | Input | Medline records |
|  | Text representation | - |
|  | Model | SVM |
|  | Corpus | Cochrane, AHRQ |
|  | Automation tool | - |
|  | Comment | - |
|  | Key result | 297 new studies were identified in traditional Cochrane (n=20) and AHRQ (n=277) review updates. Performance of the new search strategies:  Clinical Query: R:0.91 Clinical Query + SVM: R:0.89 Clinical Query + SVM + Related Articles: R:0.997 In 277 AHRQ papers:  Clinical Query: P:0.11 Clinical Query + SVM: P:0.11 Clinical Query + SVM + Related Articles:P:0.08 |
|  | Google Scholar citations | 25 |
| Trypsteen, 2016 (137) | PMID | 27482456 |
|  | Title | Diagnostic utility of droplet digital PCR for HIV reservoir quantification |
|  | Automation stage, aim | Full text selection; full-text selection via keyword search |
|  | Input | Full-text |
|  | Text representation | Full text |
|  | Model | - |
|  | Corpus | - |
|  | Automation tool | Linux Bash [https://www.ncbi.nlm.nih.gov/pmc/articles/PMC4967968/] |
|  | Comment | - |
|  | Key result | Title/abstract screening was omitted, WLS:100%, sensitivity was not evaluated |
|  | Google Scholar citations | 40 |
| Ji, 2017 (88) | PMID | 28302519 |
|  | Title | Using ontology-based semantic similarity to facilitate the article screening process for systematic reviews |
|  | Automation stage, aim | Record screening (active learning); screening using medical ontology-based semantic network representation of articles |
|  | Input | Title, abstract |
|  | Text representation | Semantic annotation (UMLS concepts to SNOMED CT and MeSH), semantic network of articles after concept optimisation and concept expansion |
|  | Model | Cosine similarity |
|  | Corpus | Oregon EPC DERP |
|  | Automation tool | MetaMap |
|  | Comment | - |
|  | Key result | In 15 SRs WSS@95: 40.98%, range5.77%-81.55% mean P:0.132@R:0.941, range(P: 0.607-0.296-@ R: 0.750-0.995) |
|  | Google Scholar citations | 47 |
| Karystianis, 2017 (62) | PMID | 28455150 |
|  | Title | Evaluation of a rule-based method for epidemiological document classification towards the automation of systematic reviews |
|  | Automation stage, aim | Record screening (automated classification); screening using rule-based extraction of targeted information from epidemiological study abstracts |
|  | Input | Abstract |
|  | Text representation | Rule-based search for information elements related to study design, population, exposure, outcome, confounders and country |
|  | Model | Rule-based matching |
|  | Corpus | National Toxicology Program HAWC (Health Assessment Workplace Collaborative) [https://hawcproject.org] |
|  | Automation tool | GATE (General Architecture for Text Mining) |
|  | Comment | - |
|  | Key result | In 35 abstracts of the test dataset, mean performance across all information elements: P:0.94@R:0.87, range by information element (P:0.54-0.97, R:0.70-0.98) |
|  | Google Scholar citations | 23 |
| Kontonatsios, 2017 (89) | PMID | 28648605 |
|  | Title | A semi-supervised approach using label propagation to support citation screening |
|  | Automation stage, aim | Record screening (active learning); extending the training set in active learning using label propagation based on the similarity of citations |
|  | Input | Title, abstract |
|  | Text representation | BOW, spectral embedded feature space (lower dimensional representation of BOW) |
|  | Model | SVM |
|  | Corpus | EPPI Center datasets (Proton Beam, COPD, Cooking Skills, Sanitation, Tobacco Packaging, Youth Development) |
|  | Automation tool | General Architecture for Text Engineering (GATE) |
|  | Comment | Various active learning strategies (certainty-based, uncertainty-based) and feature representations: baseline, BOW, spectral) |
|  | Key result | Best performance: uncertainty based active learning with spectral embedding representation. Utility gain (linear combination of yield and screening burden) of 1.7%-9.6% over baseline method. |
|  | Google Scholar citations | 43 |
| Lewis, 2017 (124) | PMID | 28978562 |
|  | Title | Developing the WCRF International/University of Bristol Methodology for Identifying and Carrying Out Systematic Reviews of Mechanisms of Exposure-Cancer Associations |
|  | Automation stage, aim | Record screening (priority ranking); prioritise the quantity of evidence about cancer exposure-associations from PubMed records |
|  | Input | Bibliographic record |
|  | Text representation | - |
|  | Model | - |
|  | Corpus | - |
|  | Automation tool | TeMMPo [ https://www.temmpo.org.uk/] |
|  | Comment | - |
|  | Key result | - |
|  | Google Scholar citations | 24 |
| Rathbone, 2017 (39) | PMID | 29178925 |
|  | Title | Expediting citation screening using PICO-based title-only screening for identifying studies in scoping searches and rapid reviews |
|  | Automation stage, aim | Search; screening by searching PICO synonyms in titles |
|  | Input | Title |
|  | Text representation | Keyword search |
|  | Model | - |
|  | Corpus | - |
|  | Automation tool | EndNote |
|  | Comment | - |
|  | Key result | In 10 SRs WLS: 11%-78%, recall of included studies: 100% in 9/10 SRs, 67% in one SR |
|  | Google Scholar citations | 33 |
| Gates, 2018 (90) | PMID | 29530097 |
|  | Title | Technology-assisted title and abstract screening for systematic reviews: a retrospective evaluation of the Abstrackr machine learning tool |
|  | Automation stage, aim | Record screening (active learning); evaluate the performance of Absrackr |
|  | Input | Title, abstract |
|  | Text representation | - |
|  | Model | - |
|  | Corpus | - |
|  | Automation tool | Abstrackr [http://abstrackr.cebm.brown.edu/] |
|  | Comment | - |
|  | Key result | Four SRs: P:0.148-0.647@R:0.788-0.936, PM:0.001, WLS:9.5%-88.4% |
|  | Google Scholar citations | 81 |
| Lanera, 2018 (40) | PMID | 29981872 |
|  | Title | Extending PubMed searches to ClinicalTrials.gov through a machine learning approach for systematic reviews |
|  | Automation stage, aim | Search: using i/e PubMed records of a SR, retrieve eligible studies from clinicaltrials.gov via text mining |
|  | Input | PubMed title, abstract ClinicalTrials.gov records |
|  | Text representation | TF-IDF, filtered for most frequently occurring terms |
|  | Model | SVM, record filtering |
|  | Corpus | International Clinical Trials Registry Platform (ICTRP) ClinicalTrials.gov [https://www.sciencedirect.com/science/article/pii/S0895435618300854#appsec1 http://clinicaltrials.gov/] |
|  | Automation tool | [R] [https://github.com/UBESP- DCTV/costumer] |
|  | Comment | - |
|  | Key result | On 14 SRs predicted number of potentially eligible RCTs from ClinicalTrials.gov. P:0.0018-0.0470@R:1.0-0.875, AUC:0.991-0.999 |
|  | Google Scholar citations | 11 |
| Langlois, 2018 (63) | PMID | 30103261 |
|  | Title | Discriminating between empirical studies and nonempirical works using automated text classification |
|  | Automation stage, aim | Record screening (automated classification); classify empirical vs non-empirical studies |
|  | Input | Title, abstract, full-text |
|  | Text representation | TF-IDF, semantic annotation (UMLS concepts), numbers, math signs: 8000 features selected for maximum information gain |
|  | Model | Decision-tree and bagging |
|  | Corpus | - |
|  | Automation tool | Automated Text Classification of Empirical Records (ATCER) [https://atcer.iro.umontreal.ca] |
|  | Comment | Various ML algorithms, feature sets and abstract / full text ratios compared |
|  | Key result | Best model: P:0.896@R:0.894, accuracy: 90.7% |
|  | Google Scholar citations | 22 |
| Przybyla, 2018 (91) | PMID | 29956486 |
|  | Title | Prioritising references for systematic reviews with RobotAnalyst: A user study |
|  | Automation stage, aim | Record screening (active learning); introduce and test RobotAnalyst for screening |
|  | Input | Title, abstract |
|  | Text representation | Term indexing, topic modelling (LDA), TF-IDF, keyword clustering |
|  | Model | SVM |
|  | Corpus | National Institute for Health and Care Excellence (NICE). Cochrane Switzerland group, at the Institute of Social and Preventive Medicine (IUMSP) |
|  | Automation tool | RobotAnalyst [http://nactem.ac.uk/robotanalyst/] |
|  | Comment | - |
|  | Key result | In 22 SRs mean WSS@95: 42.97% (range 6.89%-70.74%) |
|  | Google Scholar citations | 82 |
| Surian, 2018 (109) | PMID | 29410356 |
|  | Title | A shared latent space matrix factorisation method for recommending new trial evidence for systematic review updates |
|  | Automation stage, aim | Record screening (review update); screen relevant clinical trials registrations for a SR update |
|  | Input | ClinicalTrials.gov records |
|  | Text representation | TF-IDF, topic model (LDA), dimension reduction (PCA) |
|  | Model | Matrix factorisation, document similarity |
|  | Corpus | - |
|  | Automation tool | - |
|  | Comment | - |
|  | Key result | Best performance in type-2 diabetes trials: matrix factorisation, median rank of relevant trial records: 59. Best performance in general Cochrane SRs: TF-IDF and document similarity, with median rank of relevant trial records: 67. |
|  | Google Scholar citations | 17 |
| Tsafnat, 2018 (64) | PMID | 29695296 |
|  | Title | Automated screening of research studies for systematic reviews using study characteristics |
|  | Automation stage, aim | Record screening (automated classification); screening by rule and dictionary-based identification of PECO elements in records |
|  | Input | Title, abstract |
|  | Text representation | - |
|  | Model | - |
|  | Corpus | - |
|  | Automation tool | General Architecture for Text Engineering (GATE) [http://gate.ac.uk] |
|  | Comment | Different PECO sets, error analysis |
|  | Key result | In 3 SRs on environmental observational studies Best performance: finding E/O, P: 0.14@R: 098, WLS: 93.7% |
|  | Google Scholar citations | 33 |
| Xiong, 2018 (110) | PMID | 30018571 |
|  | Title | A Machine Learning Aided Systematic Review and Meta-Analysis of the Relative Risk of Atrial Fibrillation in Patients With Diabetes Mellitus |
|  | Automation stage, aim | Record screening (review update); manual vs automated screening based on cluster analysis |
|  | Input | Title, abstract |
|  | Text representation | BOW |
|  | Model | K-means clustering, maximum entropy classification |
|  | Corpus | - |
|  | Automation tool | [R] |
|  | Comment | - |
|  | Key result | WLS: 87%, all relevant full-text papers were retrieved |
|  | Google Scholar citations | 93 |
| Bannach-Brown, 2019 (92) | PMID | 30646959 |
|  | Title | Machine learning algorithms for systematic review: reducing workload in a preclinical review of animal studies and reducing human screening error |
|  | Automation stage, aim | Record screening (active learning); select screener and optimise performance after reclassification of human/machine discrepancies (human error analysis) |
|  | Input | Title, abstract |
|  | Text representation | Trigram BOW |
|  | Model | SVM |
|  | Corpus | - |
|  | Automation tool | SyRF, [R] [https://syrf.org.uk] |
|  | Comment | Human error analysis |
|  | Key result | P:0.559&R:0.987 |
|  | Google Scholar citations | 17 |
| Brockmeier, 2019 (93) | PMID | 31805934 |
|  | Title | Improving reference prioritisation with PICO recognition |
|  | Automation stage, aim | Record screening (active learning); improve screener performance by extending the feature set with words tagged for PICO contexts |
|  | Input | Title, abstract |
|  | Text representation | BOW (GENIA) + PICO-based named entity recognition, topic modelling (LDA) |
|  | Model | PICO recognition: BiLSTM classification: logistic regression model with L2-regularisation |
|  | Corpus | EBM-NLP DERP CAMARADES [https://aclanthology.org/P18-1019/  https://dmice.ohsu.edu/cohenaa/systematic-drug-class-review-data.html  www.camarades.info. http://www.dcn.ed.ac.uk/camarades/default.htm] |
|  | Automation tool | - |
|  | Comment | Model compared with other SR automation tools (RobotAnalyst, SWIFT-Review) and text representations (BOW w/wo PICO to BioBERT) |
|  | Key result | mean WSS@95 was 40.2%, 3.3% improvement vs baseline on 15 DERP reviews |
|  | Google Scholar citations | 37 |
| Cawley, 2019 (65) | PMID | 31711016 |
|  | Title | Novel text analytics approach to identify relevant literature for human health risk assessments: A pilot study with health effects of in utero exposures |
|  | Automation stage, aim | Record screening (automated classification); filter human studies from a large database of candidate references |
|  | Input | Title, abstract |
|  | Text representation | - |
|  | Model | Voting of 6 parallel algorithms: k-Means and Nonnegative Matrix Factorisation with 3-3 cluster sizes |
|  | Corpus | US Environmental Protection Agency (EPA) HERO |
|  | Automation tool | DoCTER |
|  | Comment | - |
|  | Key result | Two pilot reviews: WLS: 0.77@R:0.83; WLS:0.79@R:0.77 |
|  | Google Scholar citations | 12 |
| Cleo, 2019 (131) | PMID | 31221212 |
|  | Title | Usability and acceptability of four systematic review automation software packages: a mixed method design |
|  | Automation stage, aim | Record screening (evaluate software); compare the usability and acceptability of four SR automation tools |
|  | Input | - |
|  | Text representation | - |
|  | Model | - |
|  | Corpus | - |
|  | Automation tool | Covidence, SRA-Helper for EndNote, Rayyan and RobotAnalyst [www.covidence.org https://github.com/CREBP/EndNoteHelper https://rayyan.qcri.org www.nactem.ac.uk/robotanalyst] |
|  | Comment | - |
|  | Key result | SRA-Helper for EndNote was favoured by participants |
|  | Google Scholar citations | 52 |
| Cramond, 2019 (144) | PMID | 30809592 |
|  | Title | The development and evaluation of an online application to assist in the extraction of data from graphs for use in systematic reviews |
|  | Automation stage, aim | Data extraction: integrate plot digitizer to a SR software and evaluate its performance |
|  | Input | - |
|  | Text representation | - |
|  | Model | - |
|  | Corpus | - |
|  | Automation tool | Graph2Data (modified WebPlotDigitizer) [ https://github.com/EPPI-Centre/Graph2Data] |
|  | Comment | - |
|  | Key result | Compared to standard method: time saved on data extraction per graph: 352 sec, 29% more accurately extracted data points (70% vs 41%) |
|  | Google Scholar citations | 36 |
| Currie, 2019 (111) | PMID | 31107871 |
|  | Title | Animal models of chemotherapy-induced peripheral neuropathy: A machine-assisted systematic review and meta-analysis |
|  | Automation stage, aim | Record screening (review update); automated review update |
|  | Input | - |
|  | Text representation | - |
|  | Model | - |
|  | Corpus | - |
|  | Automation tool | SyRF (retrieved from reference) [https://syrf.org.uk (retrieved from reference)] |
|  | Comment | - |
|  | Key result | On a random sample of 10% from screened publications P:0.56@R:0.97 |
|  | Google Scholar citations | 15 |
| Gates, 2019 (94) | PMID | 31727150 |
|  | Title | Performance and usability of machine learning for screening in systematic reviews: a comparative evaluation of three tools |
|  | Automation stage, aim | Record screening (active learning); compare Abstrackr, DistillerSR, RobotAnalyst in screening |
|  | Input | Title, abstract, MeSH terms |
|  | Text representation | - |
|  | Model | - |
|  | Corpus | - |
|  | Automation tool | Abstrackr, DistillerSR, RobotAnalyst [http://abstrackr.cebm.brown.edu http://www.evidencepartners.com http://www.nactem.ac.uk/robotanalyst/] |
|  | Comment | - |
|  | Key result | Median values over 3 SRs:  Abstrackr: WLS:40%@PM:0.01, time saved:61 hours Distiller SR: WLS:49%@PM:0.02, time saved:92 hours RobotAnalyst: WLS:35%@PM:0.02, time saved:64 hours |
|  | Google Scholar citations | 55 |
| Lam, 2019 (125) | PMID | 30622070 |
|  | Title | Low-calorie sweeteners and health outcomes: A demonstration of rapid evidence mapping (rEM) |
|  | Automation stage, aim | Record screening (priority ranking), Data extraction; introduce the rapid evidence mapping process (automated workflow from screening to semi-automated PECO extraction), comparison with a benchmark traditional evidence-mapping study |
|  | Input | Title, abstract, full-text |
|  | Text representation | - |
|  | Model | - |
|  | Corpus | - |
|  | Automation tool | SWIFT-Active Screener, SWIFT-Review [https://www.sciome.com/swift-activescreener/] |
|  | Comment | - |
|  | Key result | Screening: WLS 46.6%@R:0.95 Identified papers: 115 full text papers (38.7%) overlapped with Wang et. al, the benchmark evidence mapping study with the same research goal, using Abstrackr for record screening. |
|  | Google Scholar citations | 15 |
| Lanera, 2019 (66) | PMID | 31810495 |
|  | Title | Screening PubMed abstracts: is class imbalance always a challenge to machine learning? |
|  | Automation stage, aim | Record screening (automated classification); explore sampling techniques to correct class imbalance in automated classification |
|  | Input | Title, abstract |
|  | Text representation | TF-IDF |
|  | Model | SVM |
|  | Corpus | International Clinical Trials Registry Platform (ICTRP) |
|  | Automation tool | - |
|  | Comment | Random over-and undersampling |
|  | Key result | AUC: 0.98-1.00 |
|  | Google Scholar citations | 15 |
| Norman, 2019 (112) | PMID | 31697361 |
|  | Title | Evaluation of an automatic article selection method for timelier updates of the Comet Core Outcome Set database |
|  | Automation stage, aim | Record screening (review update); rank records for regular updating a SR (database) of studies reporting the development of core outcomes |
|  | Input | Title, abstract |
|  | Text representation | BOW |
|  | Model | Logistic regression with L2 regularisation |
|  | Corpus | Core Outcome Measures in Effectiveness Trials (COMET) [ http://www.comet-initiative.org/studies/search] |
|  | Automation tool | - |
|  | Comment | Various weights to adjust data imbalance |
|  | Key result | WLS:75%@PM:0.02 |
|  | Google Scholar citations | 12 |
| Norman, 2019 (95) | PMID | 31661028 |
|  | Title | Measuring the impact of screening automation on meta-analyses of diagnostic test accuracy |
|  | Automation stage, aim | Record screening (active learning); 0 |
|  | Input | Title, abstract |
|  | Text representation | BOW |
|  | Model | Logistic regression |
|  | Corpus | Limsi-Cochrane dataset CLEF dataset [http://doi.org/10.5281/zenodo.1303259 https://github.com/CLEF-TAR/tar] |
|  | Automation tool | Evaluate the impact of automated screening on the results of meta-analyses in diagnostic accuracy studies [sensitivity analysis of meta-analyses based on the number of included studies] |
|  | Comment | - |
|  | Key result | Sensitivity and specificity can be estimated with ±2% accuracy if 40% of studies are missed from the meta-analysis.  Active screening with estimated recall of 95% resulted in ±0.1% accuracy.  At ∼70% WLS, estimation error was ±1.3%. |
|  | Google Scholar citations | 15 |
| Odintsova, 2019 (67) | PMID | 31464998 |
|  | Title | Genomics of human aggression: current state of genome-wide studies and an automated systematic review tool |
|  | Automation stage, aim | Record screening (automated classification); extend a SR by automated screening of a broad set of records after training on manual screening results |
|  | Input | Title, abstract |
|  | Text representation | - |
|  | Model | - |
|  | Corpus | - |
|  | Automation tool | ASR (Automated Systematic Review) [https://github.com/Rensvandeschoot/automated-systematic-review] |
|  | Comment | - |
|  | Key result | 39.1% saving on screening time (23.5 hours) |
|  | Google Scholar citations | 39 |
| Olorisade, 2019 (68) | PMID | 31075531 |
|  | Title | The use of bibliography enriched features for automatic citation screening |
|  | Automation stage, aim | Record screening (automated classification); add reference lists to title, abstract and mesh terms to improve classification performance in screening |
|  | Input | Title, abstract, MeSH terms, citation list |
|  | Text representation | BOW (binary, TF, TF-IDF), most relevant features from BOW, word2vec |
|  | Model | SVM |
|  | Corpus | Software engineering: Hall, Wahono, Radjenovic, Kitchenham Medical:  Oregon EPC DERP (TREC 2004) [https://doi.org/10.5281/zenodo.837298 https://doi.org/10.5281/zenodo.83729] |
|  | Automation tool | - |
|  | Comment | - |
|  | Key result | Adding citations is likely to improve or sustain the performance or record classification.  For the best model, WSS:1%-70%@R:0.83-1.0 |
|  | Google Scholar citations | 12 |
| Pradhan, 2019 (143) | PMID | 30257185 |
|  | Title | Automatic extraction of quantitative data from ClinicalTrials.gov to conduct meta-analyses |
|  | Automation stage, aim | Data extraction; examine the replicability of meta-analyses via extracting data from clinicaltrials.gov using exact |
|  | Input | XML of ClinicalTrials.gov results page |
|  | Text representation | - |
|  | Model | - |
|  | Corpus | ClinicalTrials.gov [https://clinicaltrials.gov] |
|  | Automation tool | EXACT [Python] [http://bio-nlp.org/EXACT] |
|  | Comment | - |
|  | Key result | 60% time reduction vs manual data extraction (4 vs 10 hours, 6 hours of time saved on 15 articles) 100% match of extracted elements with data posted on ClinicalTrials.gov 86.6% match with data elements in published SRs 21 / 28 (75%) meta-analyses in 3 SRs (15 RCTs) could be reproduced. |
|  | Google Scholar citations | 21 |
| Schmitz, 2019 (41) | PMID | 30995361 |
|  | Title | Potential Technologies Review: A hybrid information retrieval framework to accelerate demand-pull innovation in biomedical engineering |
|  | Automation stage, aim | Search, Record screening (priority ranking and query optimisation); search query optimisation and screening by priority ranking to enhance information retrieval for potential technologies reviews |
|  | Input | Title, abstract |
|  | Text representation | BOW, query-document vector space model with similarity weights |
|  | Model | - |
|  | Corpus | Oregon EPC DERP (5 SRs) NIEHS (1 SR) Innovative Laboratory Diagnostics (ILD) |
|  | Automation tool | [Python], Apache Solr |
|  | Comment | - |
|  | Key result | WSS@95:91%, overall test recall 76% vs manual screening |
|  | Google Scholar citations | 0 |
| Soboczenski, 2019 (147) | PMID | 31068178 |
|  | Title | Machine learning to help researchers evaluate biases in clinical trials: a prospective, randomized user study |
|  | Automation stage, aim | Risk of bias assessment; evaluate RobotReviewer performance and reviewer experience in semi-automated risk of bias assessment through a web interface |
|  | Input | Full-text |
|  | Text representation | - |
|  | Model | - |
|  | Corpus | - |
|  | Automation tool | RobotReviewer [https://github.com/ijmarshall/robotreviewer] |
|  | Comment | - |
|  | Key result | Time saving per article with RobotReviewer: 25% (mean time 755s vs 824s) Overall agreement of RobotReviewer with final risk of bias assessments: P:0.87, R:0.90, with positive user experience |
|  | Google Scholar citations | 33 |
| Westgate, 2019 (134) | PMID | 31355546 |
|  | Title | revtools: An R package to support article screening for evidence synthesis |
|  | Automation stage, aim | Record screening (convenience tool); introduce an r package as convenience tool for multiple screening tasks from deduplication, NLP text pre-processing and topic modelling and task management |
|  | Input | Bibliographic record |
|  | Text representation | - |
|  | Model | - |
|  | Corpus | - |
|  | Automation tool | revtools [R] [https://cran.rproject.org/package=revtools https:// revtools.net] |
|  | Comment | - |
|  | Key result | syntax is demonstrated over a SR case study |
|  | Google Scholar citations | 130 |
| Alharbi, 2020 (42) | PMID | 33067630 |
|  | Title | Refining Boolean queries to identify relevant studies for systematic review updates |
|  | Automation stage, aim | Search; improve performance of Boolean query expressions |
|  | Input | - |
|  | Text representation | - |
|  | Model | - |
|  | Corpus | - |
|  | Automation tool | [Python] |
|  | Comment | Boolean query transformation and selection |
|  | Key result | 10.3% increase in recall and ~50% decrease in screening workload vs baseline. Mean recall:0.669. |
|  | Google Scholar citations | 5 |
| Ambalavanan, 2020 (69) | PMID | 33059047 |
|  | Title | Using the contextual language model BERT for multi-criteria classification of scientific articles |
|  | Automation stage, aim | Record screening (automated classification); compare the screening performance of various BERT-based ensemble model structures |
|  | Input | Title, abstract |
|  | Text representation | SciBERT |
|  | Model | Feed Forward Neural Network (Individual Task Learner) |
|  | Corpus | Clinical Hedges [0] |
|  | Automation tool | - |
|  | Comment | Various sampling ratio and text length settings |
|  | Key result | P:0.295 @ R:0.985 |
|  | Google Scholar citations | 31 |
| Armijo-Olivo, 2020 (148) | PMID | 32065732 |
|  | Title | Comparing machine and human reviewers to evaluate the risk of bias in randomized controlled trials |
|  | Automation stage, aim | Risk of bias assessment; compare RobotReviewer with humans in risk of bias assessment |
|  | Input | Full-text |
|  | Text representation | - |
|  | Model | - |
|  | Corpus | Cochrane Database of Systematic Reviews |
|  | Automation tool | RobotReviewer [https://robot-reviewer.vortext. systems/] |
|  | Comment | - |
|  | Key result | 97% agreement with humans in classifying low / high or uncertain RoB 70.4% agreement w humans in classifying low/uncertain/high RoB |
|  | Google Scholar citations | 10 |
| Brunskill, 2020 (43) | PMID | 32069194 |
|  | Title | A Microsoft Excel Approach to Reduce Errors and Increase Efficiency in Systematic Searching |
|  | Automation stage, aim | Search: create search syntaxes more efficiently using Microsoft excel |
|  | Input | - |
|  | Text representation | - |
|  | Model | - |
|  | Corpus | - |
|  | Automation tool | [Microsoft Excel] |
|  | Comment | - |
|  | Key result | na |
|  | Google Scholar citations | 4 |
| Callaghan, 2020 (96) | PMID | 33248464 |
|  | Title | Statistical stopping criteria for automated screening in systematic reviews |
|  | Automation stage, aim | Record screening (active learning); propose a method for reliable active learning stopping criteria with predefined confidence, and test on real-life data |
|  | Input | Abstract |
|  | Text representation | BOW |
|  | Model | SVM |
|  | Corpus | TREC 2004 Genomics Track Proton Beam COPD FASTREAD |
|  | Automation tool | [Python] [https://github.com/mcallaghan/rapid-screening] |
|  | Comment | - |
|  | Key result | On average 17% workload saved @95% recall with 95% confidence |
|  | Google Scholar citations | 24 |
| Clark, 2020 (18) | PMID | 32004673 |
|  | Title | A full systematic review was completed in 2 weeks using automation tools: a case study |
|  | Automation stage, aim | Search, Record screening (), Full text selection, risk of bias assessment, Reporting; automate multiple stages of an SR completed in 2 weeks. document and evaluate the process. |
|  | Input | - |
|  | Text representation | - |
|  | Model | - |
|  | Corpus | - |
|  | Automation tool | Systematic Review Accelerator (WordFreq , Search Refinery, Polyglot Search, Deduplicator, SRA Helper, SARA, RevMan Replicant), RobotSearch, RobotReviewer, EndNote [http://sr-accelerator. com/#/help/wordfreq  http://sr-accelerator.com/#/polyglot http://sr-accelerator.com/#/help/dedupe http://sr- accelerator.com/#/sra-helper https://robotsearch.vortext.systems/ https://endnote.com/ http://sr- accelerator.com/#/  https://robotreviewer.vortext.systems/  http://sr-accelerator.com/#/replicant] |
|  | Comment | - |
|  | Key result | Time to submission: 9 working days (66 working hours), 71 working hours until published paper |
|  | Google Scholar citations | 175 |
| Dhrangadhariya, 2020 (70) | PMID | 32570395 |
|  | Title | Machine Learning Assisted Citation Screening for Systematic Reviews |
|  | Automation stage, aim | Record screening (automated classification); citation screening for a SR with narrow research question |
|  | Input | Title, abstract |
|  | Text representation | 300-dimensional vectors using word2vec, FastText |
|  | Model | Logistic Regression (LR), Support Vector Machines (SVM), k-nearest neighbour (KNN), Decision Trees-CART (DT), Random Forest (RF), and CNNs |
|  | Corpus | - |
|  | Automation tool | Gensim [https://github.com/travisbrady/word2phrase https://radimrehurek.com/gensim/models/word2vec.html https://radimrehurek.com/gensim/models/fasttext.html] |
|  | Comment | Random oversampling to correct class imbalance |
|  | Key result | The authors did not select the best algorithm LR+word2vec: P:0.898@R:0.855 SVM+FastText: P:0.891@R:0.882 KNN+word2vec: P:0.886@R:0.830 DT+FastText: P:0.835@R:0.820 RF+word2vec: P:0.869@R:0.892 CNN+word2vec: P:0.903@R:0.748 |
|  | Google Scholar citations | 5 |
| Gates, 2020 (97) | PMID | 33243276 |
|  | Title | Decoding semi-automated title-abstract screening: findings from a convenience sample of reviews |
|  | Automation stage, aim | Record screening (active learning); evaluate the performance of automated screening, its effects on SR conclusions and SR-based determinants of the variability of performance |
|  | Input | Title, abstract |
|  | Text representation | - |
|  | Model | - |
|  | Corpus | - |
|  | Automation tool | Abstrackr [http://abstrackr.cebm.brown.edu] |
|  | Comment | Two strategies: single reviewer + automation and dual reviewer + automation |
|  | Key result | On 16 SRS PM:0.0-0.14, median WLS: 26 hours Due to the omission of one study, the results of 1 out of 8 meta-analyses would change in a SR The trial design and research question influenced recall. |
|  | Google Scholar citations | 10 |
| Giummarra, 2020 (98) | PMID | 31451565 |
|  | Title | Evaluation of text mining to reduce screening workload for injury-focused systematic reviews |
|  | Automation stage, aim | Record screening (active learning), Full text selection; compare single-reviewer manual screening and full text selection vs single-reviewer automation, and overall performance of combined results (manual + automated) vs two manual reviewers |
|  | Input | Title, abstract, keywords, full-text |
|  | Text representation | Keyword search in full-text |
|  | Model | - |
|  | Corpus | - |
|  | Automation tool | Abstrackr, Wordstat, QDA Miner |
|  | Comment | - |
|  | Key result | Single reviewer automation vs manual work,  citation screening via Abstrackr: P:0.295@R:0.881, PM:0.036, WLS: 82.9%, time saved: 73.3% (44.7 hours); full-text selection via text-mining: 77.7% time saved (30.5 hours) Combined results (single manual + single automated) vs two manual reviewers: time saving on screening: 36.6%, time saving on full-text selection: 38.7% |
|  | Google Scholar citations | 21 |
| Hamel, 2020 (99) | PMID | 33059590 |
|  | Title | An evaluation of DistillerSR's machine learning-based prioritization tool for title/abstract screening - impact on reviewer-relevant outcomes |
|  | Automation stage, aim | Record screening (active learning); evaluate the performance of DistillerSR in screening |
|  | Input | - |
|  | Text representation | - |
|  | Model | - |
|  | Corpus | - |
|  | Automation tool | DistillerSR |
|  | Comment | - |
|  | Key result | On 10 SRs WLS: 30%-72.5%@R:0.95, PM: 0.0%, time saved: 11.3-198.0 hours (median: 29.8 hours). Median time saved per SR when accounting for avoided full-text screening: 36 hours. |
|  | Google Scholar citations | 41 |
| Li, 2020 (100) | PMID | 32585932 |
|  | Title | Semi-Supervised Text Classification Framework: An Overview of Dengue Landscape Factors and Satellite Earth Observation |
|  | Automation stage, aim | Record screening (active learning); deep active learning: record screening via text scoring and bidirectional long-short term memory network (BiLSTM) |
|  | Input | Title, abstract |
|  | Text representation | Text scoring by keyword search with predefined importance weights, word2vec with CBOW |
|  | Model | BiLSTM |
|  | Corpus | - |
|  | Automation tool | - |
|  | Comment | - |
|  | Key result | P:0.77, R or PM were not evaluated. WLS: 87.5% |
|  | Google Scholar citations | 11 |
| Noel-Storr, 2020 (44) | PMID | 32798713 |
|  | Title | Cochrane Centralised Search Service showed high sensitivity identifying randomized controlled trials: A retrospective analysis |
|  | Automation stage, aim | Search; evaluate the search process for inclusion in Cochrane central |
|  | Input | Bibliographic record, trial registry record |
|  | Text representation | - |
|  | Model | - |
|  | Corpus | Cochrane CENTRAL |
|  | Automation tool | Cochrane CENTRAL |
|  | Comment | - |
|  | Key result | RCTs in Cochrane CENTRAL vs 650 RCTs included in Cochrane reviews in 2017-2018 recall: 97.5% |
|  | Google Scholar citations | 38 |
| Reddy, 2020 (113) | PMID | 33076975 |
|  | Title | Comparison of a traditional systematic review approach with review-of-reviews and semi-automation as strategies to update the evidence |
|  | Automation stage, aim | Record screening (review update); compare 3 screening approaches for review updates: manual screening, review of reviews, automated screening |
|  | Input | - |
|  | Text representation | - |
|  | Model | - |
|  | Corpus | - |
|  | Automation tool | RobotAnalyst, Abstrackr |
|  | Comment | - |
|  | Key result | Performance vs manual review updating (recall measured on included full-text papers) Review of reviews: WLS:96%@R:0.56 RobotAnalyst (trained on 30% of citations): WLS:1%@R:1.00, WLS: 7%@R:0.97, WLS: 26%@R:0.74 RobotAnalyst (trained on all i/e citations): WLS:1%@R:1.00, WLS: 7%@R:0.93, WLS: 59%@R:0.69 Abstrackr: WLS:1%@R:0.98, WLS:11%@R:0.84, WLS:45%@R:0.80 |
|  | Google Scholar citations | 14 |
| Thiabaud, 2020 (45) | PMID | 32795992 |
|  | Title | Social, Behavioral, and Cultural factors of HIV in Malawi: Semi-Automated Systematic Review |
|  | Automation stage, aim | Search, Record screening (topic modelling); automated database search and full-text retrieval. record screening based on topic-modelling from full-text. |
|  | Input | Full-text, title, abstract |
|  | Text representation | TF-IDF, topic model (LDA) |
|  | Model | - |
|  | Corpus | - |
|  | Automation tool | [Python] [https://gitlab.com/Pezam/asr_freeze_hivmalawijmir] |
|  | Comment | - |
|  | Key result | reducing screening task of 16942 records to 519 took 5 days |
|  | Google Scholar citations | 9 |
| Tsou, 2020 (132) | PMID | 32241297 |
|  | Title | Machine learning for screening prioritization in systematic reviews: comparative performance of Abstrackr and EPPI-Reviewer |
|  | Automation stage, aim | Record screening (active learning, review update); 0 |
|  | Input | - |
|  | Text representation | - |
|  | Model | - |
|  | Corpus | - |
|  | Automation tool | Abstrackr, EPPI-Reviewer [performance in active screening vs review updating compared] |
|  | Comment | - |
|  | Key result | Active screening 9 SRs @R:1.00 Abstrackr: WLS: 0.7%-48.5% EPPI-Reviewer: WLS: 1.2%-60.1% Review update 9 SRs @R:1.00 Abstrackr: WLS: 0.0%-60.2% EPPI Reviewer: WLS: 2.9%-60.9% Better performance in greater reviews with >2500 citations |
|  | Google Scholar citations | 42 |
| Weisser, 2020 (46) | PMID | 32195145 |
|  | Title | A clustering approach for topic filtering within systematic literature reviews |
|  | Automation stage, aim | Search: explore document clustering to help screening |
|  | Input | Bibliographic record |
|  | Text representation | TF-IDF, latent semantic analysis (LSA) |
|  | Model | K--means clustering |
|  | Corpus | - |
|  | Automation tool | - |
|  | Comment | - |
|  | Key result | process is shown but screening performance is not evaluated |
|  | Google Scholar citations | 33 |
| Yamada, 2020 (101) | PMID | 33262102 |
|  | Title | Deep Neural Network for Reducing the Screening Workload in Systematic Reviews for Clinical Guidelines: Algorithm Validation Study |
|  | Automation stage, aim | Record screening (active learning); vector representation and cosine similarity in active learning |
|  | Input | Abstract |
|  | Text representation | 1000 dimensional vector combining word2vec and doc2vec representation of abstracts |
|  | Model | - |
|  | Corpus | SRs RCTs of medications with reproducible search in guidelines of the  American Diabetes Association  American College of Cardiology American Heart Association American Stroke Association |
|  | Automation tool | Concept Encoder, Gensim [https://www.fronteo.com/en/products/conceptencoder/] |
|  | Comment | - |
|  | Key result | For included studies in 8 SRs of RCTs of medications mean WSS@100: 0.953 mean WSS@95: 0.904 |
|  | Google Scholar citations | 10 |
| Aum, 2021 (71) | PMID | 34717768 |
|  | Title | srBERT: automatic article classification model for systematic review using BERT |
|  | Automation stage, aim | Record screening (automated classification); compare the screening performance of various BERT models with general ml models |
|  | Input | Title, abstract |
|  | Text representation | SrBERT |
|  | Model | SrBERT |
|  | Corpus | - |
|  | Automation tool | srBERT, Google Cloud Platform |
|  | Comment | Various sampling ratio, training vocabulary, and hyperparameter settings |
|  | Key result | P:0.689@R:0.911 |
|  | Google Scholar citations | 11 |
| Chai, 2021 (102) | PMID | 33795003 |
|  | Title | Research Screener: a machine learning tool to semi-automate abstract screening for systematic reviews |
|  | Automation stage, aim | Record screening (active learning); evaluate research screener |
|  | Input | Bibliographic record |
|  | Text representation | Paragraph embedding |
|  | Model | Deep learning |
|  | Corpus | - |
|  | Automation tool | Research Screener [https://researchscreener.com] |
|  | Comment | - |
|  | Key result | WS:0.68-0.96@R:1.0 for SRs, WS:0.6-0.62@R:1.0 for scoping reviews |
|  | Google Scholar citations | 36 |
| Clark, 2021 (135) | PMID | 34057072 |
|  | Title | The Impact of Systematic Review Automation Tools on Methodological Quality and Time Taken to Complete Systematic Review Tasks: Case Study |
|  | Automation stage, aim | Record screening (), Full text selection, risk of bias assessment; automate multiple stages of the SR process and compare vs manual review. |
|  | Input | - |
|  | Text representation | - |
|  | Model | - |
|  | Corpus | - |
|  | Automation tool | Systematic Review Accelerator (Polyglot Search, Deduplicator, SRA Helper), RobotReviewer |
|  | Comment | - |
|  | Key result | 30 working hours saved with automation vs manual work, with similar methodological quality |
|  | Google Scholar citations | 11 |
| Gaskins, 2021 (103) | PMID | 31875459 |
|  | Title | Factors influencing implementation of aerobic exercise after stroke: a systematic review |
|  | Automation stage, aim | Record screening (active learning); increase review credibility by increasing recall via post-protocol automated screening |
|  | Input | - |
|  | Text representation | - |
|  | Model | - |
|  | Corpus | - |
|  | Automation tool | Rayyan |
|  | Comment | - |
|  | Key result | 4 (+6.8%) eligible full text identified by 3% extra screening workload |
|  | Google Scholar citations | 16 |
| Gates, 2021 (139) | PMID | 34403225 |
|  | Title | Creating Efficiencies in the Extraction of Data From Randomized Trials: A Prospective Evaluation of a Machine Learning and Text Mining Tool |
|  | Automation stage, aim | Data extraction; prospectively compare automated data extraction (via exact) vs human work |
|  | Input | Full-text |
|  | Text representation | - |
|  | Model | - |
|  | Corpus | - |
|  | Automation tool | ExaCT [https://exact.cluster.gctools.nrc.ca/ExactDemo/intro.php ] |
|  | Comment | - |
|  | Key result | In 75 RCTs reporting of data elements were identified with median 91% accuracy, with 17% time saved per reviewer (3.7 hours) and 44.6% time saved (14.4 hours) if one reviewer was replaced by the automation tool |
|  | Google Scholar citations | 6 |
| Paynter, 2021 (47) | PMID | 33755394 |
|  | Title | A Prospective Comparison of Evidence Synthesis Search Strategies Developed With and Without Text-Mining Tools |
|  | Automation stage, aim | Search; compare usual practice vs text-mining in the construction of search syntaxes |
|  | Input | - |
|  | Text representation | - |
|  | Model | - |
|  | Corpus | - |
|  | Automation tool | 6 text mining tools (AntConc, PubReMiner, MeSH on Demand, Yale MeSH Analyzer, Carrot2, VOSviewer) |
|  | Comment | - |
|  | Key result | In seven SRs Usual practice: R:0.92, NNR: 83  Text mining: R: 0.85, NNR: 90 Similar recall with lower screening burden for text mining in complex reviews. |
|  | Google Scholar citations | 13 |
| Pham, 2021 (104) | PMID | 34039433 |
|  | Title | Text mining to support abstract screening for knowledge syntheses: a semi-automated workflow |
|  | Automation stage, aim | Record screening (active learning); 0 |
|  | Input | Title, abstract |
|  | Text representation | 3 vectors: 1) PoS tagging, semantic annotation (UMLS concepts), feature dimension reduction (SVD), 2) topic modelling, 3) word embedding, distance matrix based on cosine similarity |
|  | Model | Random Forest |
|  | Corpus | SR: insulin for type 1 diabetes; scoping review: knowledge synthesis methods [https://knowledgetranslation.net/text-mining-to-support-abstract-screening-for-knowledge-syntheses-a-workflow-approach/] |
|  | Automation tool | [R], RysannMD, GloVe [https://www.ncbi.nlm.nih.gov/pmc/articles/PMC8152711/bin/13643_2021_1700_MOESM1_ESM.docx] |
|  | Comment | SMOTE to mitigate data imbalance |
|  | Key result | SR: P:0.71@R:0.88, WLS: 63%, PM: 0.00 Scoping review: P: 0.72@R:0.89, WLS: 55%, PM: 0.013 |
|  | Google Scholar citations | 17 |
| Qin, 2021 (118) | PMID | 33485929 |
|  | Title | Natural language processing was effective in assisting rapid title and abstract screening when updating systematic reviews |
|  | Automation stage, aim | Record screening (review update); review update via an ensemble of BERT-based classifiers and GBM |
|  | Input | Title, abstract |
|  | Text representation | Four BERT models: BERT, Bio-BERT, Sci-BERT, BlueBERT |
|  | Model | LightGBM |
|  | Corpus | - |
|  | Automation tool | [Python] |
|  | Comment | - |
|  | Key result | 2-year update: WLS: 57.4%@R:0.97, PM:0.00 3-year update: WLS: 59.4%@R:0.93, PM: 0.0037 4-year update:  WLS:59.4%@R:0.95, PM: 0.0028 |
|  | Google Scholar citations | 36 |
| Stansfield, 2021 (114) | PMID | 34747151 |
|  | Title | Applying machine classifiers to update searches: Analysis from two case studies |
|  | Automation stage, aim | Record screening (review update); reduce screening workload when updating public health SRs |
|  | Input | Title, abstract |
|  | Text representation | A) BOW B) na |
|  | Model | A) logistic regression B) na |
|  | Corpus | Trials Register of Promoting Health Interventions (TRoPHI) Database of Promoting Health Effectiveness Reviews (DoPHER) [https://eppi.ioe.ac.uk/webdatabases4/Intro.aspx?ID=12 https://eppi.ioe.ac.uk/webdatabases4/Intro.aspx?ID=9] |
|  | Automation tool | EPPI-Reviewer 4 [https://eppi.ioe.ac.uk/cms/Default.aspx?tabid=3753] |
|  | Comment | - |
|  | Key result | A) TRoPHI reviews: P:0.081-0.123@R:0.992-0.997, WLS: 43.3-50.9% B) EPPI-Reviewer: WLS:61%@R:0.98 |
|  | Google Scholar citations | 6 |
| Surian, 2021 (115) | PMID | 34922458 |
|  | Title | The automation of relevant trial registration screening for systematic review updates: an evaluation study on a large dataset of ClinicalTrials.gov registrations |
|  | Automation stage, aim | Record screening (review update); screen relevant clinical trials registrations for a SR update |
|  | Input | ClinicalTrials.gov records |
|  | Text representation | BOW, TF-IDF, topic model and Doc2vec |
|  | Model | Document similarity |
|  | Corpus | - |
|  | Automation tool | - |
|  | Comment | - |
|  | Key result | Best performance: TF-IDF and document similarity, 99 trial records to screen to find all relevant updates for an updated SR |
|  | Google Scholar citations | 2 |
| Thomas, 2021 (72) | PMID | 33171275 |
|  | Title | Machine learning reduced workload with minimal risk of missing studies: development and evaluation of a randomized controlled trial classifier for Cochrane Reviews |
|  | Automation stage, aim | Record screening (automated classification); tag RCTs in Cochrane central |
|  | Input | Title, abstract |
|  | Text representation | BOW |
|  | Model | SVM ensemble |
|  | Corpus | Cochrane Crowd Cochrane CENTRAL Clinical Hedges [http://crowd.cochrane.org/DownloadData.php https://www.cochranelibrary.com/central] |
|  | Automation tool | Cochrane RCT Classifier [https://github.com/alan-turing-institute/DSSG19-Cochrane/blob/dev/analyses/partner_baseline/create_model.py https://github.com/ijmarshall/robotsearch] |
|  | Comment | Error analysis |
|  | Key result | On the Clinical Hedges Dataset (n=49 025) P:0.08@R:0.99, WLS: 58% |
|  | Google Scholar citations | 61 |
| Vaghela, 2021 (48) | PMID | 33835932 |
|  | Title | Using a Secure, Continually Updating, Web Source Processing Pipeline to Support the Real-Time Data Synthesis and Analysis of Scientific Literature: Development and Validation Study |
|  | Automation stage, aim | Search, Data extraction; a prioritise documents for manual curation and annotation for a covid-related living literature database |
|  | Input | Full-text from web crawler |
|  | Text representation | Amazon Kendra index for COVID-related natural language queries |
|  | Model | - |
|  | Corpus | REDASA [https://github.com/PanSurg/redasa-sample-data] |
|  | Automation tool | REDASA COVID dataset, [Amazon] |
|  | Comment | - |
|  | Key result | On 1424 manually curated articles: peer reviewed literature: 70.9% (1009/1424) relevant to COVID-related query: 50.5% (719/1424) quality: high (13.6%, 193/1424), medium (51.0%, 726/1424) Within peer reviewed papers:  SR: 9.4% (98/1009) RCT: 0.3% (3/1009) |
|  | Google Scholar citations | 2 |
| van Altena, 2021 (73) | PMID | 34390193 |
|  | Title | Training sample selection: Impact on screening automation in diagnostic test accuracy reviews |
|  | Automation stage, aim | Record screening (automated classification); explore screening performance on various training set sizes selected at random or based on document similarity in cross-review learning |
|  | Input | Title, abstract, with ICD codes added |
|  | Text representation | TF-IDF, cosine similarity |
|  | Model | Random Forest |
|  | Corpus | 2017 CLEF eHealth Lab ‘Technologically Assisted Reviews in Empirical Medicine Overview’ |
|  | Automation tool | - |
|  | Comment | - |
|  | Key result | From 50 DTA SRs screening performance of one review if including in the training set 49 other DTA reviews: WSS@95:49% 10 similar DTA reviews: WSS@95:43% 10 random DTA reviews: WSS@95:39% 1 similar DTA review: WSS@95: 36% 1 random DTA review: WSS@95: 25% |
|  | Google Scholar citations | 3 |
| van den Bulk, 2021 (116) | PMID | 35024621 |
|  | Title | Automatic classification of literature in systematic reviews on food safety using machine learning |
|  | Automation stage, aim | Record screening (review update); compare machine learning models, their ensembles and resampling techniques in reducing screening workload in food safety SRs and SR updates |
|  | Input | Title, abstract |
|  | Text representation | None for LSTM and BERT TF-IDF for other models |
|  | Model | SVM + Naive Bayes ensemble |
|  | Corpus | - |
|  | Automation tool | [Python] [https://github.com/WFSRBigData/systematic-review-classification] |
|  | Comment | 8 models and their ensembles compared including BERT, LSTM, AdaBoost, Gradient Boosting, Logistic Regression, Naive Bayes, SVM, Random Forest Sampling ratio adjusted by SMOTE and synthetic oversampling |
|  | Key result | Best model: SVM + Naive Bayes ensemble with 0.25 probability threshold Test set: P:0.65@R:0.95 |
|  | Google Scholar citations | 10 |
| Ajiji, 2022 (74) | PMID | 35501476 |
|  | Title | Feasibility study and evaluation of expert opinion on the semi-automated meta-analysis and the conventional meta-analysis |
|  | Automation stage, aim | Record screening (automated classification), Data extraction, Evidence synthesis, Reporting; automate multiple steps of the entire SR workflow. compare via manual SR by expert opinion. |
|  | Input | Bibliographic record |
|  | Text representation | Named Entity Recognition |
|  | Model | Naive Bayes |
|  | Corpus | - |
|  | Automation tool | MetaPreg [http://metapreg.org/] |
|  | Comment | - |
|  | Key result | 10.7 working days saved vs manual review, with similar confidence in results by expert evaluation |
|  | Google Scholar citations | 5 |
| Carey, 2022 (105) | PMID | 35654270 |
|  | Title | A text-mining tool generated title-abstract screening workload savings: performance evaluation versus single-human screening |
|  | Automation stage, aim | Record screening (active learning); compare Abstrackr performance with single human screener using Covidence |
|  | Input | Bibliographic record |
|  | Text representation | - |
|  | Model | - |
|  | Corpus | - |
|  | Automation tool | Abstrackr |
|  | Comment | - |
|  | Key result | P:0.15@R:0.91, WLS: 67% (5.4 days) vs single human reviewer at the default stopping criterion. No relevant full-text papers were missed. |
|  | Google Scholar citations | 3 |
| Carlson, 2022 (126) | PMID | 35580034 |
|  | Title | Systematic Evidence Map for Over One Hundred and Fifty Per- and Polyfluoroalkyl Substances (PFAS) |
|  | Automation stage, aim | Record screening (priority ranking), Full text selection; screening of human health-related studies in a systematic evidence map study of per-and polyfluoroalkyl substances (pfas) |
|  | Input | Title, abstract |
|  | Text representation | Bibliographic records |
|  | Model | - |
|  | Corpus | - |
|  | Automation tool | SWIFT Review, Distiller SR |
|  | Comment | - |
|  | Key result | SWIFT-Reviewer:  WLS:59%@R:0.96 Overall WLS for the entire project including SWIFT Reviewer and Distiller SR: 84.9% |
|  | Google Scholar citations | 15 |
| De Menezes, 2022 (141) | PMID | 35073506 |
|  | Title | Examining the Intersection between Gender, Community Health Workers, and Vector Control Policies: A Text Mining Literature Review |
|  | Automation stage, aim | Data extraction; extract locations for geographic distribution analysis of topics |
|  | Input | Title, abstract |
|  | Text representation | - |
|  | Model | - |
|  | Corpus | - |
|  | Automation tool | newsmap [R] |
|  | Comment | - |
|  | Key result | na |
|  | Google Scholar citations | 0 |
| Halamoda-Kenzaoui, 2022 (127) | PMID | 34050552 |
|  | Title | Toxic effects of nanomaterials for health applications: How automation can support a systematic review of the literature? |
|  | Automation stage, aim | Record screening (priority ranking), Full text selection, risk of bias assessment, Data extraction; screen records, select full-text and extract predefined parameters from papers meeting minimum information criteria related to nanomaterial toxicity |
|  | Input | Title, abstract, full-text |
|  | Text representation | CoNLL-U, keyword search |
|  | Model | - |
|  | Corpus | - |
|  | Automation tool | SwiftReview, segmentR [R], Poppler [https://github.com/ec-jrc/jrc_f2_refine/tree/master/segmenteR https://github.com/ec-jrc/jrc_f2_refine https://www.sciome.com/swift-review/ https://poppler.freedesktop.org/] |
|  | Comment | - |
|  | Key result | The study demonstrated the potential of using a sequence of automation systems in a SR |
|  | Google Scholar citations | 3 |
| Ishankulov, 2022 (75) | PMID | 35673014 |
|  | Title | The Classification of Scientific Abstracts Using Text Statistical Features |
|  | Automation stage, aim | Record screening (automated classification); evaluate the usefulness of simple text statistical features in screening |
|  | Input | Title, abstract |
|  | Text representation | Features from text statistics based on characters, words, keywords, 3 dimensional word2vec embedding |
|  | Model | Random Forest |
|  | Corpus | - |
|  | Automation tool | - |
|  | Comment | Various feature set combinations |
|  | Key result | AUC:0.777 for best scenario including keywords, word2vec as features |
|  | Google Scholar citations | 1 |
| Jackson, 2022 (49) | PMID | 35420700 |
|  | Title | Mindfulness for smoking cessation |
|  | Automation stage, aim | Search; extending the manual search for a SR using the human behaviour change project automated search strategy |
|  | Input | - |
|  | Text representation | - |
|  | Model | - |
|  | Corpus | - |
|  | Automation tool | Microsoft Academic (search strategy of the Human Behaviour Change Project) |
|  | Comment | - |
|  | Key result | Automated search: 112 additional records over manual screening, (+3.1%). The number of included papers that were identified by automation only was not reported. |
|  | Google Scholar citations | 526 |
| Jardim, 2022 (149) | PMID | 35676632 |
|  | Title | Automating risk of bias assessment in systematic reviews: a real-time mixed methods comparison of human researchers to a machine learning system |
|  | Automation stage, aim | Risk of bias assessment; evaluate two RobotReviewer platforms in risk of bias assessment |
|  | Input | Full-text |
|  | Text representation | - |
|  | Model | - |
|  | Corpus | - |
|  | Automation tool | RobotReviewer, EPPI Reviewer [http://www.robotreviewer.net] |
|  | Comment | - |
|  | Key result | Agreement: RobotReviewer vs human assessments: 83%, human vs human assessments: 81% (RR:1.02). Despite similar performance to humans, the acceptability of RobotReviewer was low |
|  | Google Scholar citations | 8 |
| Li, 2022 (76) | PMID | 35771807 |
|  | Title | Automating document classification with distant supervision to increase the efficiency of systematic reviews: A case study on identifying studies with HIV impacts on female sex workers |
|  | Automation stage, aim | Record screening (automated classification); refined text representation models for automated classification |
|  | Input | Title, abstract |
|  | Text representation | TF-IDF, keyword search, MeSH keyword-based clusters, mix of MeSH keyword clusters and most relevant tokens |
|  | Model | Random Forest |
|  | Corpus | HIV in female sex workers [https://github.com/lebao0215/TextDataAbstracts] |
|  | Automation tool | [R] |
|  | Comment | Various machine learning algorithms and feature representations |
|  | Key result | WSS@95: 37% |
|  | Google Scholar citations | 1 |
| Muller, 2022 (128) | PMID | 34919321 |
|  | Title | Machine learning in systematic reviews: Comparing automated text clustering with Lingo3G and human researcher categorization in a rapid review |
|  | Automation stage, aim | Record screening (priority ranking); automated screening was used in a study focusing on document clustering |
|  | Input | - |
|  | Text representation | - |
|  | Model | - |
|  | Corpus | - |
|  | Automation tool | EPPI Reviewer |
|  | Comment | - |
|  | Key result | na |
|  | Google Scholar citations | 14 |
| O'Keefe, 2022 (50) | PMID | 35841125 |
|  | Title | Investigation of text-mining methodologies to aid the construction of search strategies in systematic reviews of diagnostic test accuracy-a case study |
|  | Automation stage, aim | Search: compare text-mining tools to extract keywords to improve search syntaxes |
|  | Input | Bibliographic record, full-text, citations |
|  | Text representation | - |
|  | Model | - |
|  | Corpus | - |
|  | Automation tool | 16 text mining tools (Anne O'Tate, BiblioShiny, Carrot2, CitNetExplorer, EndNote, Keyword‐Analyzer, Lingo3G, Lingo4G, MeSH on Demand, PubReMiner, TerMine, Text Analyzer, Tm for R, VOSviewer, Voyant,Yale MeSH Analyser) |
|  | Comment | User experiences were also surveyed |
|  | Key result | In addition to 76 included papers, 11 (+14.5%) were identified by the extended search using 40 additional keywords by all included tools. The text miners differed in user experience and performance. Text Analyser, Yale MeSH Analyzer and PubReminer retrieved most additional papers. |
|  | Google Scholar citations | 1 |
| Proescholdt, 2022 (77) | PMID | 35854734 |
|  | Title | Testing a filtering strategy for systematic reviews: evaluating work savings and recall |
|  | Automation stage, aim | Record screening (automated classification); evaluate the performance of publication type filtering in screening |
|  | Input | Title, abstract, MeSH terms |
|  | Text representation | - |
|  | Model | - |
|  | Corpus | Oregon EPC DERP [https://www.ohsu.edu/evidence-based-practice-center/derp-reports https://databank.illinois.edu/datasets/IDB-9257002] |
|  | Automation tool | Multi-Tagger, web RCT Tagger |
|  | Comment | - |
|  | Key result | in 10 SRs mean WLS: 33.6%@R:0.983 |
|  | Google Scholar citations | 0 |
| Sanchez-Graillet, 2022 (151) | PMID | 35659056 |
|  | Title | Synthesizing evidence from clinical trials with dynamic interactive argument trees |
|  | Automation stage, aim | Evidence synthesis: automated evidence summaries for clinical trials organised as superiority claims |
|  | Input | Full-text |
|  | Text representation | Resource Description Framework (RDF) files using the C-TrO Ontology |
|  | Model | - |
|  | Corpus | - |
|  | Automation tool | DIAeT [https://scdemo.techfak.uni-bielefeld.de/ratio-argviz/] |
|  | Comment | System usability survey |
|  | Key result | na |
|  | Google Scholar citations | 1 |
| Schneider, 2022 (78) | PMID | 35571360 |
|  | Title | Evaluation of publication type tagging as a strategy to screen randomized controlled trial articles in preparing systematic reviews |
|  | Automation stage, aim | Record screening (automated classification); evaluate the performance of RCT tagger |
|  | Input | Cochrane reviews in XML format |
|  | Text representation | Extract PMIDs |
|  | Model | - |
|  | Corpus | Cochrane Review Groups [https://github.com/infoqualitylab/Tagger_Evaluation] |
|  | Automation tool | RCT Tagger [http://arrowsmith.psych.uic.edu/cgi-bin/arrowsmith_uic/RCT_Tagger.cgi] |
|  | Comment | - |
|  | Key result | 6693 articles retrieved from Cochrane reviews with RCT-only inclusion criteria Error analysis of low-scoring articles with RCT probability score <0.01 R:0.986, if using RCT Tagger, on average, 0.05 RCT would be missed per Cochrane review |
|  | Google Scholar citations | 2 |
| Tsubota, 2022 (79) | PMID | 36038066 |
|  | Title | Improvement of intervention information detection for automated clinical literature screening during systematic review |
|  | Automation stage, aim | Record screening (automated classification); tag intervention in PubMed abstracts with information obtained from clinicaltrials.gov |
|  | Input | PubMed records with NCT ID, ClinicalTrials.gov records |
|  | Text representation | BIO tagging of interventions, SciBERT to identify methods or results sections in abstracts, PubMedBERT for named entity recognition (NER) of interventions |
|  | Model | PubMed Bert |
|  | Corpus | EBM-NLP ClinicalTrials.gov [https://ebm-nlp.herokuapp.com https://www.clinicaltrials.gov/api/gui] |
|  | Automation tool | [Python] [https://doi.org/10.17632/ccfnn3jb2x.1] |
|  | Comment | Various training data compared: manual labelling, automatic labelling and mix of the two, also adaptive weighting was applied to negative sentences (with no information on intervention) |
|  | Key result | Best model: weighted manual (EBM-NLP) + automatically labelled training data P:0.714@R:0.861, F1: 0.780 |
|  | Google Scholar citations | 0 |
| Valizadeh, 2022 (106) | PMID | 35655155 |
|  | Title | Abstract screening using the automated tool Rayyan: results of effectiveness in three diagnostic test accuracy systematic reviews |
|  | Automation stage, aim | Record screening (active learning); evaluate Rayyan in the screening of diagnostic test accuracy SRs |
|  | Input | Title, abstract |
|  | Text representation | BOW |
|  | Model | SVM |
|  | Corpus | - |
|  | Automation tool | Rayyan [http://rayyan.qcri.org] |
|  | Comment | - |
|  | Key result | 3 SRs on diagnostic test accuracy After screening 20% of records, at 1.5 stars exclusion threshold  for records selected for full-text screening P:0.22@R:0.99 for records included in the final review At 1.5 stars threshold P:0.38@R:0.99 |
|  | Google Scholar citations | 3 |
| van Lissa, 2022 (133) | PMID | 34036150 |
|  | Title | Mapping Phenomena Relevant to Adolescent Emotion Regulation: A Text-Mining Systematic Review |
|  | Automation stage, aim | Record screening (na); screen abstracts for a text-mining SR |
|  | Input | Bibliographic record |
|  | Text representation | - |
|  | Model | Naive Bayes |
|  | Corpus | - |
|  | Automation tool | Rayyan, ASReview [https://github.com/cjvanlissa/veni_sysrev] |
|  | Comment | Synthesis via text-mining (out of scope) |
|  | Key result | na |
|  | Google Scholar citations | 6 |
| Viner, 2022 (80) | PMID | 35040870 |
|  | Title | School Closures During Social Lockdown and Mental Health, Health Behaviours, and Well-being Among Children and Adolescents During the First COVID-19 Wave: A Systematic Review |
|  | Automation stage, aim | Record screening (active learning, review update); screening in a covid-related SR |
|  | Input | - |
|  | Text representation | - |
|  | Model | - |
|  | Corpus | - |
|  | Automation tool | EPPI-Reviewer 4 |
|  | Comment | - |
|  | Key result | From 16817 records the authors screened 1500 to train EPPI-Reviewer 4 |
|  | Google Scholar citations | 267 |
| Walker, 2022 (140) | PMID | 34920276 |
|  | Title | Evaluation of a semi-automated data extraction tool for public health literature-based reviews: Dextr |
|  | Automation stage, aim | Data extraction; identify, extract, and connect typical data entities of environmental health experimental animal studies. |
|  | Input | Full-text |
|  | Text representation | Embedding: GloVe, ELMo, encoder: BiLSTM and CRF |
|  | Model | DNN |
|  | Corpus | National Institute for Standards and Technology, Text Analysis Conference, Systematic Review Information Extraction track (NIST TAC SRIE) |
|  | Automation tool | Dextr [https://www.niehs.nih.gov/research/atniehs/labs/iha/dextr/index.cfm] |
|  | Comment | - |
|  | Key result | Performance vs a manually annotated gold standard: respiratory endpoints associated with exposure to biocides in 51 experimental animal studies. Manual reviewers in pairs: P:0.925@R:0.918, mean time per study: 971s Semi-automated review (reviewer + Dextr): P:0.932@R:0.838, mean time per study: 517s |
|  | Google Scholar citations | 7 |
| Wang, 2022 (81) | PMID | 36180888 |
|  | Title | PICO entity extraction for preclinical animal literature |
|  | Automation stage, aim | Record screening (automated classification), Data extraction; BERT-based PICO extraction from preclinical study abstracts automatically screened |
|  | Input | Title, abstract |
|  | Text representation | Sentence-level BERT classification (contains/not contains PICO) followed by entity-level classification of PICO elements (BERT vs BiLSTM-CRF) |
|  | Model | - |
|  | Corpus | - |
|  | Automation tool | [Python] [ https://osf.io/2dqcg] |
|  | Comment | BERT, Bio BERT, PubMed BERT-abs, PubMed BERT-full |
|  | Key result | PubMedBERT trained on abstracts achieved highest performance.  PICO sentence: P:085@R:0.885 PICO entity (overall): P:0.68@R:0.74 |
|  | Google Scholar citations | 2 |
| Wang, 2022 (150) | PMID | 34709718 |
|  | Title | Risk of bias assessment in preclinical literature using natural language processing |
|  | Automation stage, aim | Risk of bias assessment; assign a binary risk of bias label to five risk of bias domains of preclinical animal studies |
|  | Input | Full-text |
|  | Text representation | BOW, word2vec, doc2vec, BioBERT, Sentence BERT, DistilBERT |
|  | Model | - |
|  | Corpus | - |
|  | Automation tool | pre-rob [Python] [https://github.com/qianyingw/pre-rob] |
|  | Comment | Compared models for BOW, dov2vec: SVM, LR, RF, for word2vec: CNN, recurrent network (RNN) with attention, hierarchical attention network (HAN), BERT with document chunk pooling or sentence extraction |
|  | Key result | Models with best performance by domain on the test set:  Random allocation: RNN + Attention F1:0.820 Blinded assessment: RNN + Attention F1:0.816 Conflict of interest: CNN F1: 0.827 Animal welfare regulations: BERT SE F1: 0.915 Animal exclusions CNN: 0.466 |
|  | Google Scholar citations | 6 |

**Abbreviations**

@R: at a specified recall level

AHRQ: Agency for Healthcare Research and Quality

AUC: area under curve

BiLSTM: bidirectional long short-term memory network

BERT: Bidirectional Encoder Representations from Transformers

BOW: bag of words

CAMARADES: Collaborative Approach to Meta Analysis and Review of Animal Experimental Studies

CBOW: continuous bag of words

CN field: corporate author field (PubMed)

CNN: convolutional neural network

COPD: chronic obstructive pulmonary disease

CoNLL-U: (Conference on Natural Language Learning) a text representation format for natural language processing

DERP: Drug Effectiveness Review Project

DNN: deep neural network

DT: decision tree

DTA: diagnostic test accuracy

ELMo: embeddings from language models

EPC: Evidence-based Practice Center

EPPI Centre: Evidence for Policy and Practice Information and Coordinating Centre

GBM: gradient boosting machine

GLM: generalised linear model

GloVE: Global Vectors for Word Representation

GRADE: Grading of Recommendations, Assessment, Development, and Evaluations

IMRAD: introduction, methods, results and discussion

KNN: K nearest neighbours

LASSO: least absolute shrinkage and selection operator

LDA: latent Dirichlet allocation

LR: logistic regression

LSTM: long short-term memory network

MeSH: Medical Subject Headings

NCT number: national clinical trial number

NLP: natural language processing

P: precision

PECO: population, exposure, comparator, outcome

PICO: patient, intervention, comparator, outcome

PM: proportion (of full-text studies) missed

PMID: PubMed identifier

PoS: part of speech

PCA: principal component analysis

R: recall

RF: random forest

RCT: randomised clinical trial

RNN: recurrent neural network

SR: systematic literature review

SMOTE: Synthetic Minority Oversampling TEchnique

SVM: support vector machine

UMLS: Unified Medical Language System

TF-IDF: term frequency – inverse document frequency

TREC: Text Retrieval Conference

WSS@95: workload saved over sampling at 95% recall

WLS: workload saved

WHO: World Health Organisation
